# GLP-1 RAs and SGLT-2 Inhibitors for Insulin Resistance in Nonalcoholic Fatty Liver Disease: Systematic Review and Network Meta-Analysis

**DOI:** 10.3389/fendo.2022.923606

**Published:** 2022-07-13

**Authors:** Hongle Yan, Chunyi Huang, Xuejun Shen, Jufang Li, Shuyi Zhou, Weiping Li

**Affiliations:** ^1^ Department of Endocrinology, The First Affiliated Hospital of Shantou University Medical College, Shantou, China; ^2^ Department of Clinical Medicine, Shantou University Medical College, Shantou, China

**Keywords:** GLP-1 RAs, SGLT-2 inhibitors, nonalcoholic fatty liver disease, insulin resistance, network meta-analysis

## Abstract

**Objective:**

Glucagon-like peptide-1 receptor agonists (GLP-1 RAs) and sodium-glucose cotransporter-2 (SGLT-2) inhibitors reduce glycaemia and weight and improve insulin resistance (IR) *via* different mechanisms. We aim to evaluate and compare the ability of GLP-1 RAs and SGLT-2 inhibitors to ameliorate the IR of nonalcoholic fatty liver disease (NAFLD) patients.

**Data Synthesis:**

Three electronic databases (Medline, Embase, PubMed) were searched from inception until March 2021. We selected randomized controlled trials comparing GLP-1 RAs and SGLT-2 inhibitors with control in adult NAFLD patients with or without T2DM. Network meta-analyses were performed using fixed and random effect models, and the mean difference (MD) with corresponding 95% confidence intervals (CI) were determined. The within-study risk of bias was assessed with the Cochrane collaborative risk assessment tool RoB.

**Results:**

25 studies with 1595 patients were included in this network meta-analysis. Among them, there were 448 patients, in 6 studies, who were not comorbid with T2DM. Following a mean treatment duration of 28.86 weeks, compared with the control group, GLP-1 RAs decreased the HOMA-IR (MD [95%CI]; -1.573[-2.523 to -0.495]), visceral fat (-0.637[-0.992 to -0.284]), weight (-2.394[-4.625 to -0.164]), fasting blood sugar (-0.662[-1.377 to -0.021]) and triglyceride (- 0.610[-1.056 to -0.188]). On the basis of existing studies, SGLT-2 inhibitors showed no statistically significant improvement in the above indicators. Compared with SGLT-2 inhibitors, GLP-1 RAs decreased visceral fat (-0.560[-0.961 to -0.131]) and triglyceride (-0.607[-1.095 to -0.117]) significantly.

**Conclusions:**

GLP-1 RAs effectively improve IR in NAFLD, whereas SGLT-2 inhibitors show no apparent effect.

**Systematic Review Registration:**

PROSPERO https://www.crd.york.ac.uk/PROSPERO/, CRD42021251704

## 1 Introduction

Nonalcoholic fatty liver disease (NAFLD) is a chronic metabolic liver disease characterized by increased lipid accumulation in hepatocytes but is not caused by clear causes related to alcohol consumption. NAFLD is often associated with central obesity, insulin resistance (IR) and in general with some symptoms of metabolic syndrome (1, 2).The global prevalence rate of NAFLD is 25%, and it is one of the most common chronic liver diseases in the world (3). Its clinical features are liver triglyceride (TG) accumulation and IR. TG in the liver is synthesized from fatty acyl-CoA. The concentration of fatty acyl-CoA is determined by the balance between the formation of fatty acids (circulating free fatty acids, *de novo* lipogenesis, TG decomposition) and utilization (lipid synthesis, β-oxidation) (4, 5). When IR occurs, the lipolysis of white lipids increases, and the synthesis of lipids decreases (6). At the same time, with the decrease in glucose utilization by skeletal muscle, more fatty acyl-CoA produced by glucose metabolism turns to *de novo* lipogenesis (7), which increases the accumulation of liver TG and even transforms into lipotoxic substances such as long-chain fatty acids, ceramides, and diacylglycerols, resulting in inflammation, endoplasmic reticulum stress, liver fibrosis and hepatocyte apoptosis (5). In short, IR increases the accumulation of lipids in the liver, leading to NAFLD occurrence and development.

For IR, GLP-1 RAs and SGLT-2 inhibitors show satisfactory efficacy in patients with type 2 diabetes mellitus (T2DM), has and have been recommended by experts from many associations (8–10). GLP-1 RAs can reduce oxidative stress (11, 12), inflammation (13), and endoplasmic reticulum stress (14), improve β-cell function (14, 15) and enhance insulin sensitivity (16–18). SGLT-2 inhibitors act on the sodium-glucose cotransporter in renal tubules to inhibit the reabsorption of glucose in renal tubules, reduce blood glucose and alleviate the effects of hyperglycemia on β-cells and IR (19–24). There are a considerable number of studies that have already compared GLP-1 RAs and SGLT-2 inhibitors in T2DM patients on variety outcomes, such as in the PIONEER-2 and SUSTAIN-8 trials, which found that similitude is superior to empagliflozin and canagliflozin in reducing HbA1c and body weight at week 52, respectively (2, 25).

At present, there is no recognized drug treatment for NAFLD (26–29), but as a metabolic disease, GLP-1 RAs and SGLT-2 inhibitors should have significant effects on IR and seem to be appropriate choices. Several studies have used GLP-1 RAs and SGLT-2 inhibitors in the treatment of NAFLD, but no study have directly compared their effects. Therefore, we conducted this systematic review and network meta-analysis to comprehensively evaluate and compare the abilities of GLP-1 RAs and SGLT-2 inhibitors to ameliorate IR in patients with NAFLD.

## 2 Methods

### 2.1 Agreement to Register

We registered the protocol for this system review at PROSPERO (CRD42021251704).

### 2.2 Search Strategy

The study team co-designed a literature search strategy to search for randomized controlled trials (RCTs) published up to March 01, 2021, in Embase, Medline, and PubMed with language limited to English (**Appendix 1**). In addition, we screened references in the included articles to look for other potential studies.

### 2.3 Study Selection

Two reviewers, working independently, screened citations and evaluated the full text of eligible studies. A third reviewer resolved disagreements by consensus.

#### 2.3.1 Eligibility Criteria

Inclusion criteria were defined using the ‘Patients, interventions, comparators, outcomes, study designs, timeframe’ (PICOST) framework, as follows:

##### 2.3.1.1 Patients

NAFLD Patients with or without T2DM, age ≥ 18.

##### 2.3.1.2 Interventions

Antidiabetic drugs, including GLP-1 RAs, SGLT-2 inhibitors, thiazolidinediones (TZDs), dipeptidyl peptidase (DPP-4), sulfonylureas (SUs), and metformin.

##### 2.3.1.3 Comparators

Control group including Placebo, standard care or another antidiabetic mentioned in interventions. All treatments should be given alone and not in combination with any other antidiabetic drugs mentioned in interventions.

##### 2.3.1.4 Outcomes

The main results of this review are based on IR-related indicators that show the degree of IR (direct indicators of IR) or influence IR (indirect indicators of IR): 1) the direct indicator of IR was the homoeostasis model assessment of insulin resistance (HOMA-IR) index; 2) the indirect indicators were adipose tissue, such as subcutaneous fat (SAT), visceral fat (VAT), weight and body mass index (BMI), and adipokines, including leptin and adiponectin. Secondary outcomes were IR-related laboratory measurements, including: 1) glycolipid metabolism, such as fasting blood sugar (FBS), total cholesterol (TC), TG, high-density lipoprotein cholesterol (HDL), low-density lipoprotein cholesterol (LDL); 2) systolic blood pressure (SBP) and diastolic blood pressure (DBP); and 3) liver enzymes aspartate aminotransferase (AST) and alanine transaminase (ALT).

##### 2.3.1.5 Study Design

RCTs reporting the mean and standard deviation of outcome indicators after interventions.

##### 2.3.1.6 Timeframe

The duration of treatment should be longer than two months.

#### 2.3.2 Other Limitation

First, the language of the publications was limited to English. Second, for studies whose results were reported in multiple publications, we excluded publications presenting duplicate data and included the publications reporting the most complete data from any study. Third, studies under the risk of low-quality (retracted, terminated and impact factor less than 1 point) were excluded. Finally, studies were excluded if the data could not be extracted.

### 2.4 Data Extraction

For each eligible study, two reviewers independently extracted the following: study characteristics (study registration number, year of publication, country or countries, funding, duration), population (setting, sample size, patient demographics, whether subjects had coexisting T2DM), intervention description (drug class, name, dose, presence or absence of lifestyle intervention, and specific type of lifestyle intervention) and results. For outcome indicators, the mean and standard deviation after intervention of each study were extracted. Reviewers resolved disagreements by discussion or, if necessary, consultation with a third reviewer.

### 2.5 Risk of Bias Assessment

The risk of bias was assessed by two reviewers independently using the Cochrane collaborative risk assessment tool RoB (30). The tool is used to determine the risk of bias in randomized trials, including seven dimensions of sources, six types of bias risk: selection bias (random sequence generation and allocation concealment), performance bias (blinding of participants and personnel), detection bias (blinding of outcome assessment), attrition bias (incomplete outcome data), reporting bias (selective reporting) and other bias (funding sources, etc.) (30). Each risk of bias evaluation dimension had three classifications: low, unclear, or high.

If the random sequence was generated correctly and hidden, the risk of selection bias was considered to be low. The risk of performance bias was deemed to be low if participants were blinded as well as those administering the treatment. If the outcome evaluator was blinded, or the outcome indicators were not influenced by evaluator subjectivity, the risk of detection bias was considered to be low. The risk of attrition bias was considered to be low if there was no missing data, or the number and cause of missing data were similar between groups, the missing data was not sufficient to affect the effect size of treatment, and the missing values are handled properly. The risk of reporting bias was required to determine whether an outcome was selectively reported by comparison of protocols and research reports.

### 2.6 Statistical Analysis

A network meta-analysis was conducted within a Bayesian framework to assess the relative effects of GLP-1 RAs and SGLT-2 inhibitors. ADDIS1.16.6 and R-3.6.2 software were used for data analysis, STATA.16 software was used to draw the network evidence graph, and risk of bias graphs were drawn by RevMan 5.3 software.

Because the outcome index was continuous variables, the mean difference (MD) and associated 95% confidence interval (95% CI) was used as the index for effect size of treatment. In this study, a network meta-analysis was conducted within a Bayesian framework to compare six hypoglycemic agents, especially to assess the relative effectiveness of GLP-1 RAs and SGLT-2 inhibitors for NAFLD.

All outcomes were analyzed by using the consistency model and the inconsistency model, the overall heterogeneity was compared based on the differences in deviance information criteria and I^2^. If the difference of deviance information criteria between the two models was ≥ 5, the inconsistency model was used. Both a fixed effect (FE) model and a random effect (RE) model were run for each result, and a more appropriate model based on the deviance information criteria, mean posterior residences, and I^2^ was chosen.

The Markov Chain Monte Carlo method was used to estimate the posterior densities of all unknown parameters in each model. Four Markov chains were initially set for simulation with 50,000 iterations, and the first 10,000 anneals were used for eliminating the effects of the initial values. The potential scale reduction factor (PSRF) was calculated to diagnose the degree of the model’s convergence. A PSRF ≥ 1.2 would indicate that the current simulation times were insufficient to achieve good convergence and more iterations were needed, a PSRF < 1.2 would indicate that convergence has been achieved, and a PSRF value close to 1 would indicate the model achieved good convergence.

The included studies were tested for consistency and inconsistency. We used node splitting approaches to assess the agreement between direct and indirect estimates in every closed loop of evidence, and a *P* > 0.05 was considered to indicate good consistency, whereas a *P≤* 0.05 was considered to indicate inconsistency. If there was evidence of material inconsistency, the specific reasons were identified by reviewing the corresponding study with further analysis.

The rank probability of each treatment was estimated by the surface under the cumulative sorting curve (SUCRA) (31). SUCRA is a percentage interpreted as the probability of a treatment that is the most effective without uncertainty on the outcome, which is equal to 1 or 0 when the treatment is certain to be the best or the worst, respectively.

## 3 Results

### 3.1 Description of the Included Studies

**Figure 1:**
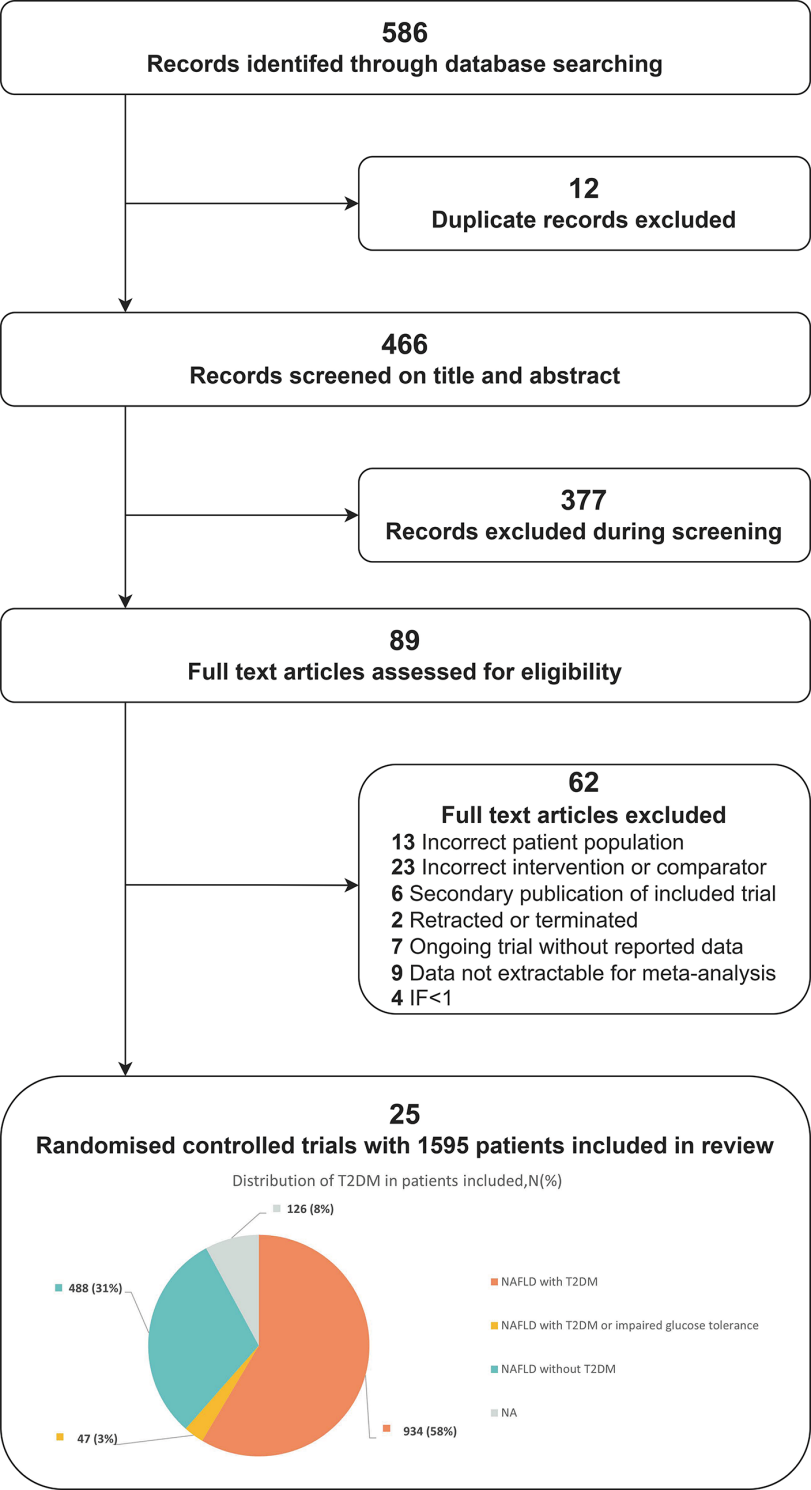
Flow diagram for the study selection.

**Figure 2:**
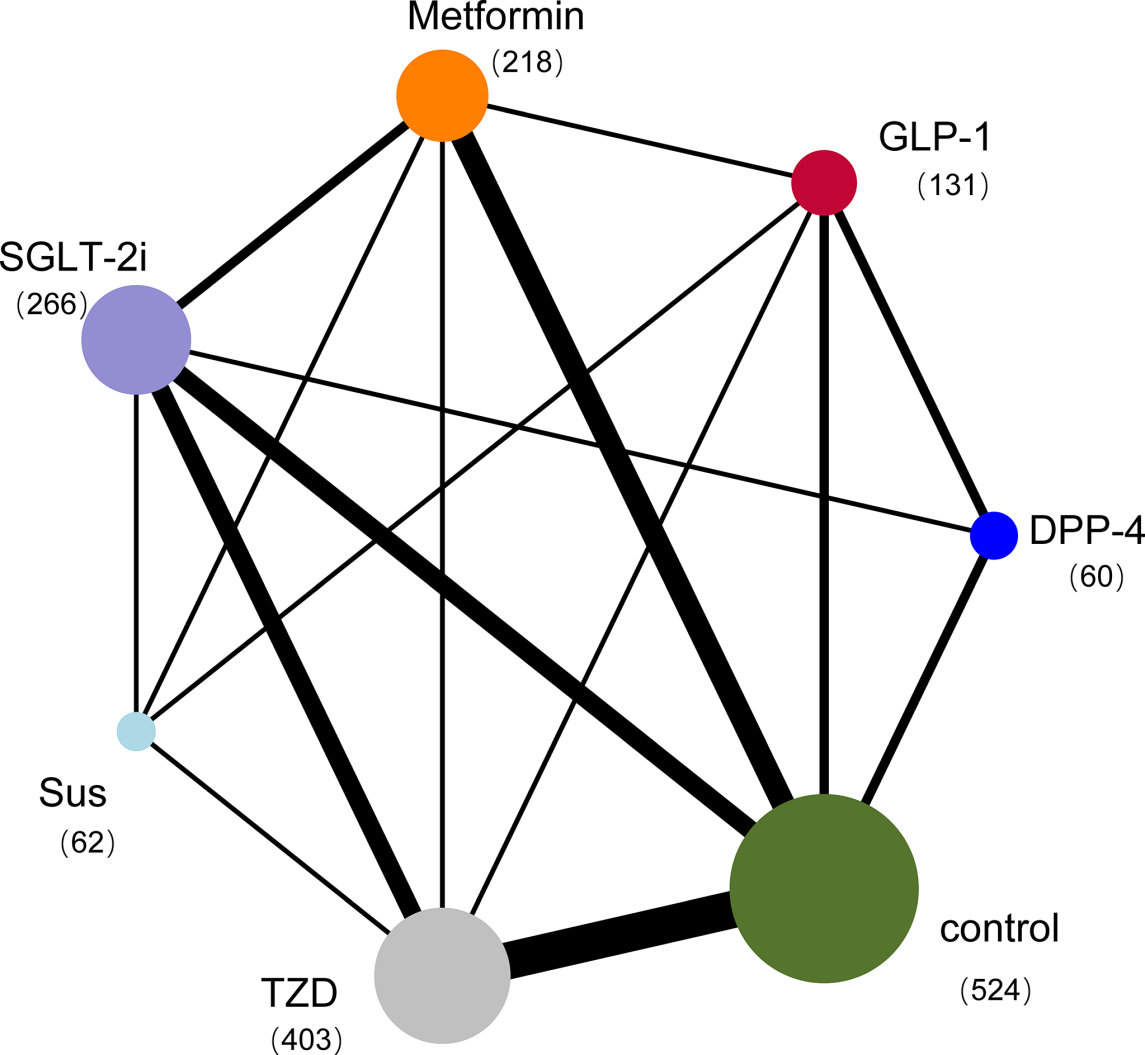
Network plot of trials evaluating glucose-lowering drugs for NAFLD. The network shows the number of participants assigned to each glucose-lowering class, and the size of each circle is proportional to the number of participants randomly assigned to treatment (sample size per drug in parentheses). The thickness of the line is proportional to the number of trials between the corresponding drugs. Compared with placebo, the most commonly compared drugs were TZDs. DPP-4=Dipeptidyl peptidase-4 inhibitors; GLP-1 RAs=Glucagon-like peptide-1 receptor agonists; SGLT-2 inhibitors=Sodium-glucose cotransporter-2 inhibitors; SUs=Sulfonylureas; TZDs=Thiazolidinediones.

The electronic search yielded 586 unique records. Screening and full-text article analysis identified 25 trials with 1595 patients (**Figure 1**) (**Appendix 2**) comparing the effects of 6 glucose-lowering drugs (GLP-1 RAs, DPP-4, SGLT-2 inhibitors, TZDs, SUs, and metformin) with placebo or standard care on IR in patients with NAFLD. The median trial mean age was 52 years, the median baseline FBS was 7.66mmol/L and the mean treatment duration was 28.86 weeks. **Figure 2** shows the treatment comparison network from the included studies. The sample sizes ranged from 12 to 162. Of the 25 studies, 13 studies indicated active lifestyle interventions, 1 showed no lifestyle intervention, and the other 11 studies did not specify whether or not they had a lifestyle intervention. In addition, 6 studies had patients with NAFLD alone without T2DM, 16 studies had patients with NAFLD and T2DM, 1 study had T2DM or impaired glucose tolerance and 2 studies did not report whether or not their patients had comorbid T2DM (**Figure 1**).

### 3.2 Risk of Bias

**Figure 3:**
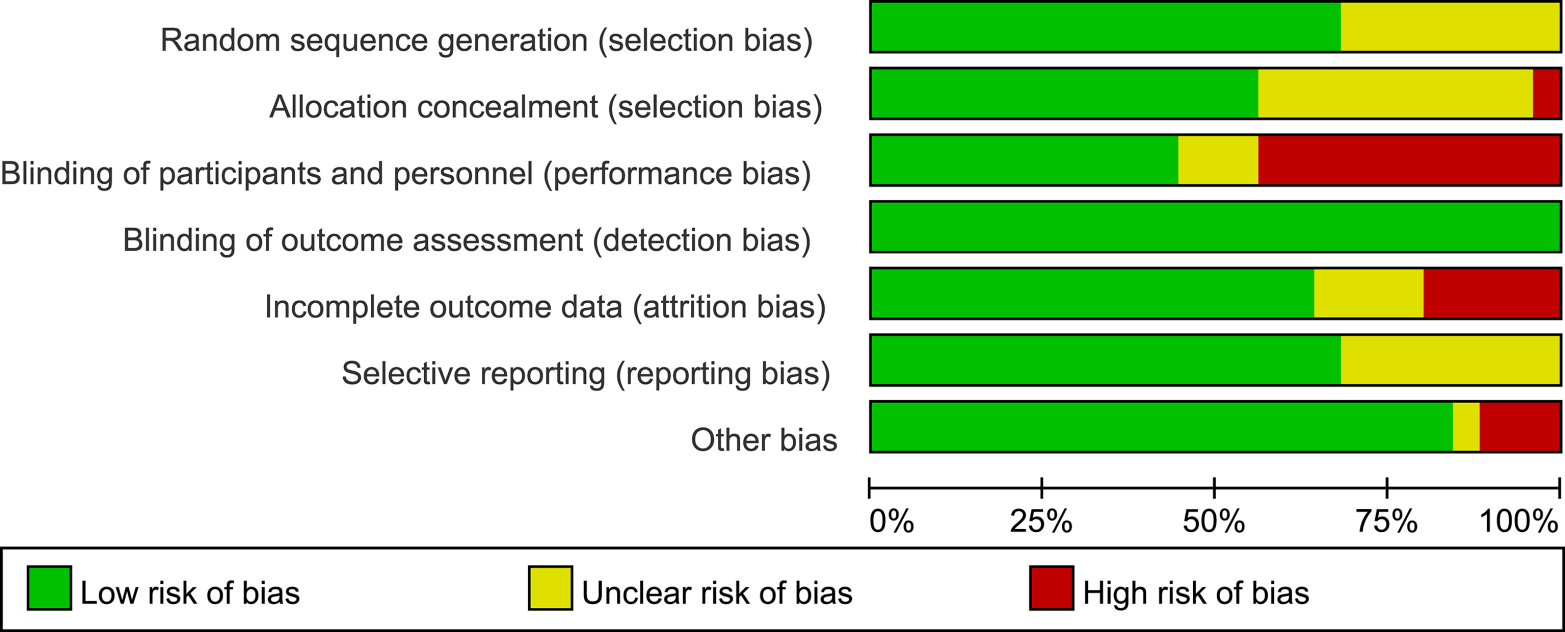
Risk of bias graph. Review of authors’ judgements about each risk of bias presented as percentages across all included studies.

**Figure 4:**
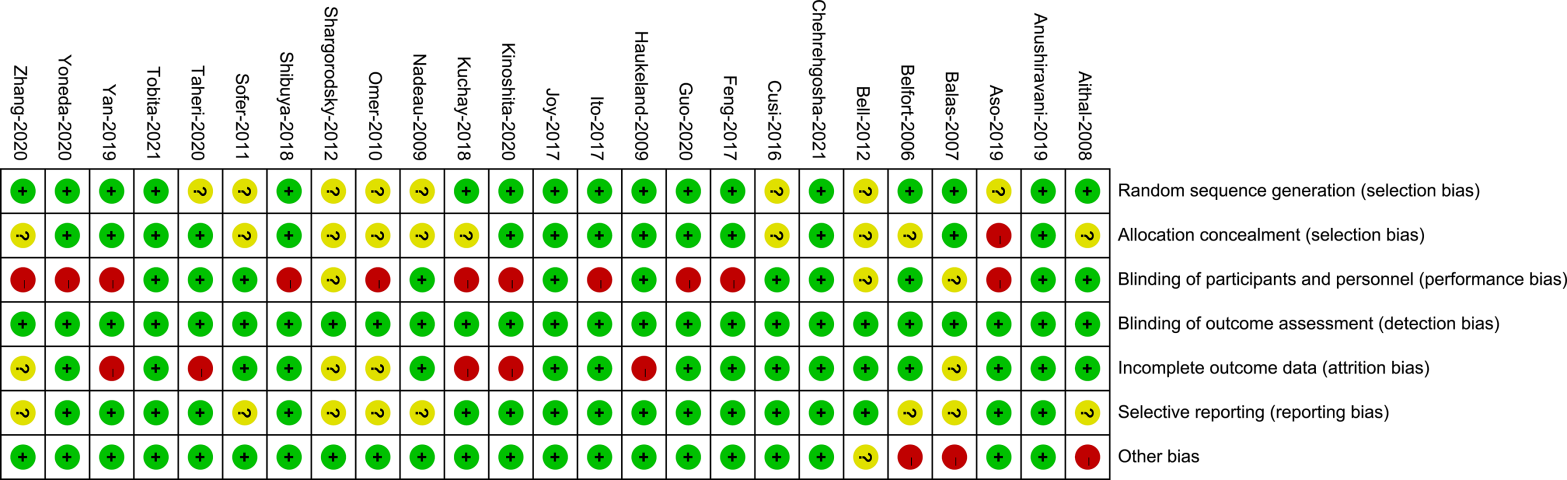
Risk of bias summary. Review of authors’ judgements about each risk of bias for each included study.

**Appendix 3** presents the risk of bias and the reasons for its determination in each trial. The key limitation was low levels of reported blinding of participants and personnel because the GLP-1 RAs were mainly administered by injection and could not be blinded. Of the 25 trials, for selection bias, 17 trials (68%) were at low risk of bias in random sequence generation, 14 trials (56%) were at low risk of bias in allocation concealment, and 11 trials (44%) were at low risk in performance bias. The outcome indicators in this analysis were all objective and were not influenced by evaluators, so the 25 trials (100%) were at low risk for detection bias. 16 trials (64%) were adjudicated as being at low risk of attrition bias, 17 trials (68%) were at low risk for reporting bias, and 21 trials (84%) were judged to have a low risk of other bias (**Figures 3**, **4**).

### 3.3 Outcomes

**Appendix 4** presents the network plot for each outcome indicator. **Appendix 8** gives a network estimate for each drug comparison for all outcomes.

#### 3.3.1 HOMA-IR

**Figure 5:**
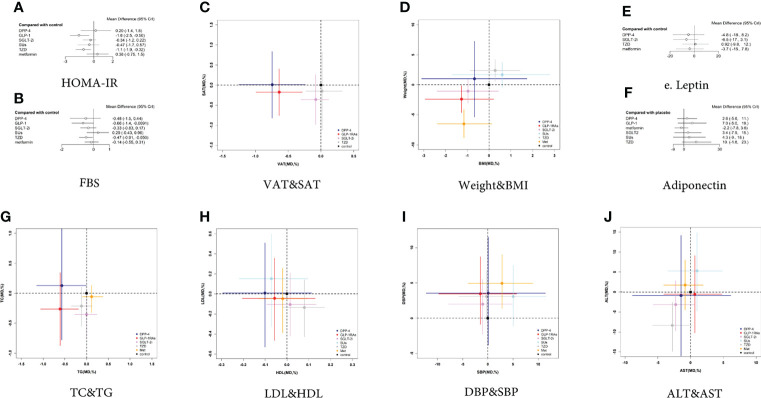
Two-dimensional graphs and forest plots for different outcome indicators. **(A)** HOMA-IR, **(B)** FBS, **(C)** VAT and SAT, **(D)** Weight and BMI, **(E)** Leptin, **(F)** Adiponectin, **(G)** TC and TG, **(H)** LDL and HDL, **(I)** DBP and SBP, **(J)** ALT and AST.

**Figure 6:**
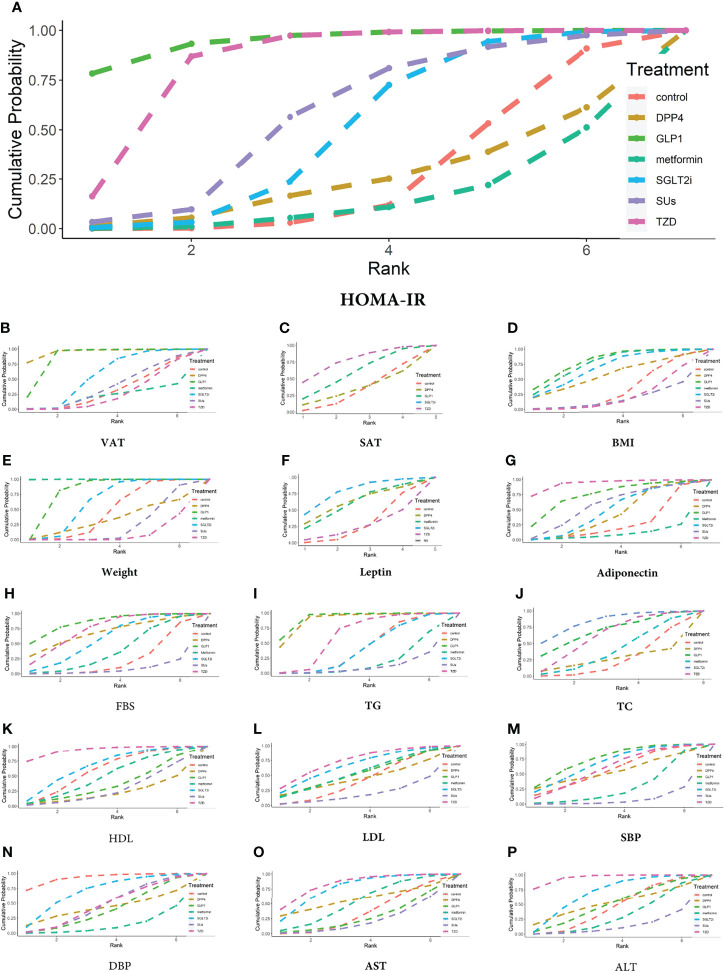
Ranking probabilities of different hypoglycemic agents for different outcome indicators. **(A)** HOMA-IR, **(B)** VAT, **(C)** SAT, **(D)** BMI, **(E)** Weight, **(F)** Leptin, **(G)** Adiponectin, **(H)** FBS, **(I)** TG, **(J)** TC, **(K)** HDL, **(L)** LDL, **(M)** SBP, **(N)** DBP, **(O)** AST, **(P)** ALT.

**Figure 7:**
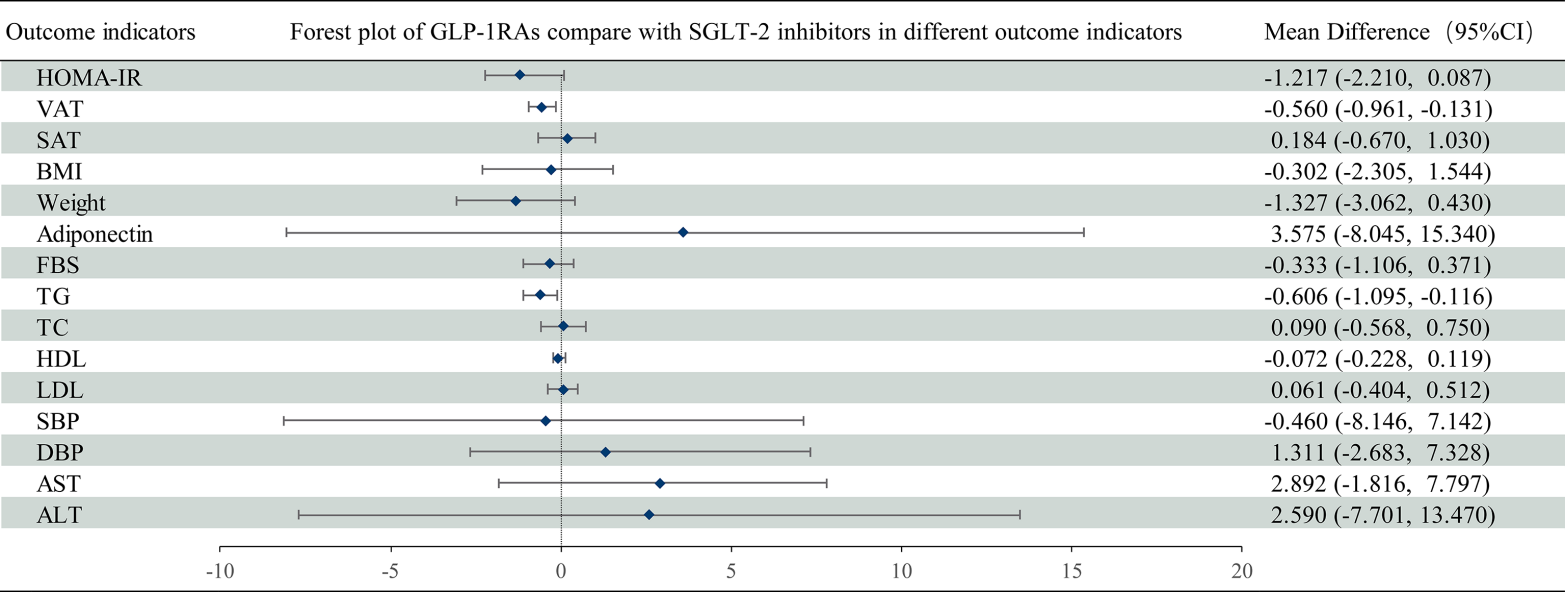
Mean difference of GLP-1 RAs compared with SGLT-2 inhibitors on different outcome indicators in NAFLD patients. Mean difference and 95% confidence intervals were derived with the use of network meta-analysis. GLP-1 RAs, Glucagon-like peptide-1 receptor agonists; SGLT-2 inhibitors, Sodium-glucose cotransporter-2 inhibitors; CI, confidence interval.

HOMA-IR was reported in 14 trials with 1153 patients (**Appendix 4; Supplementary Figure 1**). Compared with the control group, GLP-1 RAs reduced the HOMA-IR (MD [95% CI]; -1.573[-2.523 to -0.495]), whereas SGLT-2 inhibitors had no statistically significant effect (MD -0.342 [-1.156 to 0.218]) (**Figure 5A**). The SUCRA chart shows that the probabilities of GLP-1 RAs and SGLT-2 inhibitors being among the top three most effective drugs were 97% and 23%, respectively (**Figure 6A**). Compare with SGLT-2 inhibitors, GLP-1 RAs showed no difference in the effect on the HOMA-IR (MD -1.217 [-2.210 to 0.087]) (**Figure 7**).

#### 3.3.2 Adipose Tissue and Adipokines

##### 3.3.2.1 VAT and SAT

The VAT was reported in 8 trials with 561 patients (**Appendix 4; Supplementary Figure 2**). Compared with the controls, GLP-1 RAs decreased VAT (MD -0.637 [-0.992 to -0.284]), whereas SGLT-2 inhibitors had no statistically significant effect (MD -0.078 [-0.308 to 0.120) (**Figure 5C**). The SUCRA chart shows that the probabilities of GLP-1 RAs and SGLT-2 inhibitors being among the top three most effective drugs were 99% and 49%, respectively (**Figure 6B**).

The SAT was reported in 4 trials with 235 patients (**Appendix 4; Supplementary Figure 3**). Compared with the control group, both GLP-1 RAs and SGLT-2 inhibitors had no statistically significant effect on SAT (MD -0.176 [-0.758 to 0.403] and -0.360 [-0.979 to 0.260], respectively) (**Figure 5C**). The SUCRA chart shows that the probabilities of GLP-1 RAs and SGLT-2 inhibitors being among the top three most effective drugs were 73% and 89%, respectively (**Figure 6C**).

Compared with SGLT-2 inhibitors, GLP-1 RAs had a higher probability of reducing VAT (MD -0.560 [-0.961 to -0.131]), whereas they did not have different effects on SAT (MD 0.184 [-0.669 to 1.030]) (**Figure 7**).

##### 3.3.2.2 BMI and Weight

BMI was reported in 18 trials with 1006 patients (**Appendix 4; Supplementary Figure 4**). Compared with the control group, GLP-1 RAs and SGLT-2 inhibitors had no statistically significant effect on BMI (MD -1.262 [-2.933 to 0.218] and -0.964 [-2.385 to 0.423], respectively) (**Figure 5D**). The SUCRA chart shows that the probabilities of GLP-1 RAs and SGLT-2 inhibitors being among the top three most effective drugs were 87% and 67%, respectively (**Figure 6D**).

Weight was reported in 19 trials with 1143 patients (**Appendix 4; Supplementary Figure 5**). As shown in **Figure 5D**, compared with the control group, GLP-1 RAs significantly reduced body weight (MD -2.394 [-4.625 to -0.164]), whereas SGLT-2 inhibitors had no effect (MD -1.059 [-3.056 to 0.931]). The SUCRA chart shows that the probabilities of GLP-1 RAs and SGLT-2 inhibitors being among the top three most effective drugs were 98% and 66%, respectively (**Figure 6E**).

Compared with SGLT-2 inhibitors, GLP-1 RAs showed no difference in the effects on BMI or weight (MD 0.501 [-1.582 to 2.434] and 0.5796 [-4.127 to 5.034], respectively) (**Figure 7**).

##### 3.3.2.3 Leptin and Adiponectin

Leptin was reported in 4 trials with 158 patients (**Appendix 4; Supplementary Figure 6**). None of the studies reported the effect of GLP-1 RAs on leptin. Compared with the control group, SGLT-2 inhibitors had no statistically significant effect on leptin (MD -6.479 [-17.4 to 3.127]) (**Figure 5E**). The SUCRA chart shows that the probabilities of SGLT-2 inhibitors being among the top three most effective drugs is 92% (**Figure 6F**).

7 trials, including 345 patients, reported adiponectin (**Appendix 4; Supplementary Figure 7**). Compared with the control group, GLP-1 RAs and SGLT-2 inhibitors had no statistically significant effect on adiponectin (MD 7.007 [-5.033 to 18.850] and 3.402 [-7.910 to 14.670], respectively) (**Figure 5F**). The SUCRA chart shows that the probabilities of GLP-1 RAs being among the top three most effective drugs was 77%, while SGLT-2 inhibitors’ was only 31% (**Figure 6G**).

There was no difference between GLP-1 RAs and SGLT-2 inhibitors in the effect on adiponectin (MD 3.575 [-8.045 to 15.340]) (**Figure 7**).

#### 3.3.3 Glucose and Lipid Metabolism

##### 3.3.3.1 FBS

FBS was reported in 20 trials with 1216 patients (**Appendix 4; Supplementary Figure 8**). Compared with the control group, GLP-1 RAs decreased the FBS (MD -0.663 [-1.377 to -0.021]), whereas SGLT-2 inhibitors had no statistically significant effect (MD -0.330 [-0.832 to 0.170]) (**Figure 5B**). The SUCRA chart shows that the probabilities of GLP-1 RAs and SGLT-2 inhibitors being among the top three most effective drugs were 89% and 47%, respectively (**Figure 6H**). GLP-1 RAs and SGLT-2 inhibitors showed no difference in the effect on FBS (MD -0.333 [-1.106 to 0.371]) (**Figure 7**).

##### 3.3.3.2 TG and TC

TG was reported in 17 trials with 986 patients (**Appendix 4; Supplementary Figure 9**). Compared with the control group, GLP-1 RAs decreased TG (MD -0.608 [-1.056 to -0.188]), whereas SGLT-2 inhibitors had no statistically significant effect (MD -0.003 [-0.279 to 0.234]) (**Figure 5G**). The SUCRA chart shows that the probabilities of GLP-1 RAs and SGLT-2 inhibitors being among the top three most effective drugs were 99% and 12%, respectively (**Figure 6I**).

TC was reported in 12 trials with 741 patients (**Appendix 4; Supplementary Figure 10**). Compared with the control group, neither GLP-1 RAs nor SGLT-2 inhibitors had any statistically significant effect on TC (MD -0.263 [-0.872 to 0.344] and -0.354 [-0.754 to 0.035], respectively) (**Figure 5G**). The SUCRA chart shows that the probabilities of GLP-1 RAs and SGLT-2 inhibitors being among the top three most effective drugs were 75% and 92%, respectively (**Figure 6J**).

Compared with SGLT-2 inhibitors, GLP-1 RAs had a higher probability of decreasing TG (MD -0.607 [-1.095 to -0.116]). However, there was no difference between GLP-1 RAs and SGLT-2 inhibitors in effect on TC (MD 0.090 [-0.568 to 0.750]) (**Figure 7**).

##### 3.3.3.3 HDL and LDL

HDL was reported in 19 trials with 1171 patients (**Appendix 4; Supplementary Figure 11**). Compared with the control group, GLP-1 RAs and SGLT-2 inhibitors had no statistically significant effect on HDL (MD -0.056 [-0.204 to 0.129]) and 0.015 [-0.092 to 0.133]) (**Figure 5H**). The SUCRA chart shows that the probabilities of GLP-1 RAs and SGLT-2 inhibitors being among the top three most effective drugs were 21% and 67%, respectively (**Figure 6K**).

LDL was reported in 19 trials with 1171 patients (**Appendix 4; Supplementary Figure 12**). Compared with the control group, GLP-1 RAs and SGLT-2 inhibitors had no statistically significant effect on LDL (MD -0.045 [-0.466 to 0.355] and -0.107 [-0.421 to 0.205], respectively) (**Figure 5H**). The SUCRA chart shows that the probabilities of GLP-1 RAs and SGLT-2 inhibitors being among the top three most effective drugs were 44% and 64%, respectively (**Figure 6L**).

Compared with SGLT-2 inhibitors, GLP-1 RAs showed no difference in effects on HDL or LDL (MD -0.072 [-0.228 to 0.119] and 0.061 [-0.404 to 0.512], respectively) (**Figure 7**).

#### 3.3.4 Blood Pressure: SBP and DBP

SBP was reported in 9 trials with 604 patients (**Appendix 4; Supplementary Figure 13**). As shown in **Figure 5I**, compared with the control group, GLP-1 RAs and SGLT-2 inhibitors had no statistically significant effect on SBP (MD -1.486 [- 9.753 to 5.709] and -1.029 [- 7.830 to 4.853], respectively). The SUCRA chart shows that the probabilities of GLP-1 RAs and SGLT-2 inhibitors being among the top three most effective drugs were 77% and 67%, respectively (**Figure 6M**).

DBP was reported in 9 trials with 604 patients (**Appendix 4; Supplementary Figure 14**). Compared with the control group, GLP-1 RAs and SGLT-2 inhibitors had no statistically significant effect on DBP (MD 3.457 [-0.877 to 8.709) and 1.990 [-2.272 to 5.526], respectively) (**Figure 5I**). The SUCRA chart shows that the probabilities of GLP-1 RAs and SGLT-2 inhibitors being among the top three most effective drugs were 22% and 75%, respectively (**Figure 6N**).

Compared with SGLT-2 inhibitors, GLP-1 RAs showed no difference in effects on SBP or DBP (MD 0.460 [-8.146 to 7.142] and 1.311 [-2.683 to 7.328], respectively) (**Figure 7**).

#### 3.3.5 Liver Function: AST and ALT

AST was reported in 20 trials with 1206 patients (**Appendix 4; Supplementary Figure 15**). As shown in **Figure 5J**, compared with the control group, GLP-1 RAs and SGLT-2 inhibitors had no statistically significant effect on AST (MD 0.643 [-4.097 to 4.777]and -2.274 [-5.712 to 0.588], respectively). The SUCRA chart shows that the probabilities of GLP-1 RAs and SGLT-2 inhibitors being among the top three most effective drugs were 13% and 84%, respectively (**Figure 6O**).

ALT was reported in 22 trials with 1312 patients (**Appendix 4; Supplementary Figure 16**). As shown in **Figure 5J**, compared with the control group, GLP-1 RAs and SGLT-2 inhibitors had no statistically significant effect on ALT (MD -0.534 [-10.180 to 9.163] and -3.136 [-9.704 to 2.860], respectively). The SUCRA chart shows that the probabilities of GLP-1 RAs and SGLT-2 inhibitors being among the top three most effective drugs were 39% and 73%, respectively (**Figure 6O**).

There was no difference between GLP-1 RAs and SGLT-2 inhibitors on AST or ALT (MD 2.892 [-1.816 to 7.797] and 2.590 [-7.701 to 13.470], respectively) (**Figure 7**).

### 3.4 Heterogeneity and Inconsistency Test

The difference value in deviance information criteria between the consistency and inconsistency models was less than 5, indicating the data have met the premise of consistency. In terms of deviance information criteria and mean posterior residuals, the RE model provided a better fit than the FE model in the analysis of all outcome indicators except for SAT and weight (**Appendix 5**). The node splitting method based on a Monte Carlo Markov Chain simulation was used to evaluate the network inconsistency of different outcome indicators, considering random-effect models, normal priors for treatment fixed effects, and uniform priors for the variances of the random effects. Supplementary materials (**Appendix 6**) show evidence of overall network inconsistencies or heterogeneity with no severe concerns of incoherence between direct and indirect evidence, and there were no local inconsistencies except for the following: (1) BMI of TZDs versus GLP-1 RAs, and TZDs versus metformin (*P* = 0.049 and 0.006, respectively); (2) FBS between TZDs and metformin (*P* = 0.046); (3) HDL level between TZDs and GLP-1 RAs, and metformin and GLP-1 RAs (*P* = 0.006 and 0.032, respectively); (4) AST level between metformin versus TZDs (*P*= 0.008); and (5) ALT level between metformin versus TZDs (*P*= 0.006). Convergence analysis shows that each Monte Carlo Markov chain achieved stable fusion from the initial part, and it could be visually analyzed in the subsequent calculation. Single chain fluctuations could not be recognized, which means the degree of convergence was high (**Appendix 7; Supplementary Figures 1–16**).

## 4 Discussion

To our knowledge, this is the first systematic review and network meta-analysis to directly compare the effects of GLP-1 RAs and SGLT-2 inhibitors on IR levels in patients with NAFLD. NAFLD is a chronic metabolic liver disease, with the main clinical manifestation being increased lipid accumulation in the liver without a clear link to alcohol consumption and is a clinical manifestation of metabolic syndrome in the liver (32). In 2020, two articles proposed that NAFLD should be renamed MAFLD (metabolic associated fatty liver disease), and experts have agreed that compared with NAFLD, MAFLD more accurately reflects the mechanism of NAFLD (32, 33).

Given the increasingly defined metabolic nature of the disease, treatments targeting metabolism will be very promising. GLP-1 RAs and SGLT-2 inhibitors are two types of drugs that treat NAFLD through metabolic targeting. We evaluated the effect of these two drugs on the degree of IR, in patients with NAFLD, by applying Bayesian network meta-analysis and showing that, compared with the control group, GLP-1 RAs can reduce HOMA-IR value, weight, VAT, FBS, and TG, whereas SGLT-2 inhibitors had no significant effect on those outcomes. In addition, in the absence of head-to-head comparisons between GLP-1 RAs and SGLT-2 inhibitors, we also found significant differences between them. Importantly, GLP-1 RAs reduced VAT content and TG levels to a greater extent than SGLT-2 inhibitors. Our results provide both direct and indirect evidence that GLP-1 RAs improves IR and has certain advantages over SGLT-2 inhibitors in ameliorating IR in NAFLD patients.

GLP-1 RAs are incretin hormones secreted by intestinal L-cells following meal ingestion, and have various metabolic functions, including: 1) inducing β-cell proliferation and reducing lipotoxic β-cell apoptosis; 2) enhancing both insulin synthesis and glucose-stimulated insulin secretion; 3) inhibiting glucagon secretion in a glucose-dependent manner; 4) reducing IR and improving peripheral insulin sensitivity through promoting weight loss caused by delayed gastric emptying and appetite suppression; and 5) increasing liver and muscle glucose uptake, followed by lowering of free fatty acid levels (34–37). A recent meta-analysis, concerning the use of GLP-1 RAs in patients with NAFLD (12 studies involving 780 patients), found significant improvements in FBS levels and HOMA-IR when the trial lasted longer than 24 weeks in subgroup analysis (38), similar to the results of our analysis. However, few studies have focused on the improvement in IR. In another RCT, GLP-1 RAs also reduced VAT in patients with polycystic ovary syndrome (39). In addition, low activity of brown adipose tissue has been associated to NAFLD (40), but none of 25 included RCTs have involved data of brown adipose tissue between groups, suggesting the need for NAFLD drug therapy studies focusing on brown adipose tissue. Mechanistically, GLP-1 RAs reduce hepatic steatosis and increases insulin sensitivity of hepatocytes through AMP-activated protein kinase, which exert an influence on insulin signaling pathways (41). At the same time, GLP-1 RAs may also reduce the expression of genes related to fatty acid synthesis, TG level or *de novo* synthesis, and the accumulation of liver and ectopic fat (42), which is consistent with the results obtained in this paper. We speculate that GLP-1 RAs improve IR and further reduce FBS and TG, as well as improve glucose and lipid metabolism by reducing VAT. This would suggest that GLP-1 RAs should be applied in NAFLD patients with IR and obesity (especially abdominal obesity), and glucose or lipid metabolic disorders. Moreover, GLP-1 RAs tended to reduce BMI, TC, SAT and LDL levels, and increase HDL and adiponectin, but these improvements were not statistically significant. In the included studies, the mean duration of medication in all 25 studies was 28.86 weeks, but for those studies using GLP-1 RAs, medication was collected after taken for only 20.4 weeks in average. The average duration of treatment with GLP-1 RAs was less than the average intervention duration of all 25 studies, which may have reduced efficacy.

SGLT-2 inhibitors are a new class of antidiabetic drugs that reduce blood sugar by inhibiting the kidney’s reabsorption of glucose and allowing excess glucose to be excreted in the urine. In short, its mechanism of action is the direct excretion of glucose instead of insulin sensitization to promote glucose transport. Its principle is similar to the dam principle, only promoting the excretion of excess glucose, which also makes the risk of hypoglycemia low. In animal studies, SGLT-2 inhibitors have reduced new fat generation and increased lipoprotein decomposition (43, 44). Based on the existing literature, SGLT-2 inhibitors have been suggested to reduce HOMA-IR, weight, BMI, SAT, VAT, FBS, TC, LDL, AST, ALT, SBP, and also increase HDL, but these improvements were not statistically significant. According to SUCRA, SGLT-2 inhibitors have more advantages than GLP-1 RAs in improving HDL, LDL, TC, AST, ALT, and DBP in NAFLD patients. Considering that some of our patients with NAFLD did not have T2DM comorbidity, their median FBS was 7.66 mmol/L, indicating glucose toxicity was not severe. In this case, due to the normal levels of glucose, the ability of SGLT-2 inhibitors to improve IR, i.e. by excreting excess glucose, would not be activated. No trial has been reported on SGLT-2 inhibitors in pure NAFLD patients without T2DM diabetes. A study on the “Effect of Empagliflozin on Liver Fat in Non-diabetic Patients” (NCT04642261) has been registered in Clinical Trials and is expected to be completed by December 31, 2022. In addition, the average duration of SGLT-2 inhibitor medication for all studies was 25.09 weeks, shorter than the average duration of intervention in the included studies overall, which may be one of the reasons why SGLT-2 inhibitors have no significant effect on IR in NAFLD patients.

The advantages of this systematic review and network meta-analysis are as follows. First, we grasp the nature of NAFLD as a metabolic disease and focus our analysis on IR as a metabolic marker. Second, a network meta-analysis is used to comprehensively measure the effects of GLP-1 RAs and SGLT-2 inhibitors on various indicators that are related to IR in patients with NAFLD, making up for the lack of direct comparison between them. Third, a network meta-analysis is used to enlarge the sample size and correct the results obtained with smaller sample size. In addition, the emergence of new studies on these two classes of drugs has created a need for updated analysis, and this article meets this need (45–55).

There are also some limitations in this study. First, there is some heterogeneity in the clinical environment of each trial. For example, due to the small number of related studies in this field, we did not limit whether the included patients had diabetes, which may lead to some heterogeneity. Still, the consistency of the results was acceptable. We also run both the RE and the FE models, choosing the appropriate model to obtain more reliable results. Second, the measurement of insulin resistance in our included trails were HOMA-IR instead of hyper-insulinemic-euglycemic clamp technology, which is internationally recognized as the gold standard. Hyper-insulinemic-euglycemic clamp technology can be applied to all study groups, but at the same time it is a complex operation and requires repeated blood puncture. HOMA-IR is suitable for large-scale evaluation of IR in research with large sample sizes (56). However, the sample size of some included trials was relatively small and the application of HOMA-IR to evaluate IR may have some defects. Therefore, we selected other indicators that are highly correlated with the degree of IR, such as SAT, VAT, BMI, TG, and adipocytokines (57–60) to assist judgment of IR and make up for this deficiency. Finally, the average duration of treatment was not balanced. For example, the average duration of treatment with GLP-1 RAs and SGLT-2 inhibitors was lower than the average duration of all included studies, which suggests that larger and longer RCTs are needed to verify our results.

## 5 Conclusion

In conclusion, this network meta-analysis provides evidence for the effect of GLP-1 RAs and SGLT-2 inhibitors on reducing IR in patients with NAFLD. This study suggests that GLP-1 RAs can improve the metabolism of NAFLD, and in this regard, the effect of SGLT-2 inhibitors still needs to be determined using rigorous long-term and large-scale RCTs.

## 6 Prospects

As one of the most prevalent chronic diseases in the world, the public health and economic impact of NAFLD has been gradually given increasing attention by patients, regulatory agencies, and biopharmaceutical organizations. Although the cure for NAFLD is still unknown, drug research and development for each link of its mechanism is underway. Due to the close relationship between NAFLD and metabolic syndrome, especially IR, this review indicates that GLP-1 RAs, but not SGLT-2 inhibitors can be used for treating NAFLD patients, based on obesity especially abdominal obesity, a high-HOMA-IR index and glucose or lipid metabolic disorder. More clinical studies targeting IR are needed to provide more evidence for improving IR and reduce the risk of chronic complications in patients with NAFLD.

## Data Availability Statement

The original contributions presented in the study are included in the article/**Supplementary Material**. Further inquiries can be directed to the corresponding author.

## Author Contributions

HY designed the study, collected the data, contributed to the statistical analysis, and served as the primary author of the manuscript. WL co-designed the study and contributed to the writing of the manuscript and provided critical feedback to shape the manuscript. CH and XS contributed to the statistical analysis and assisted with the writing of the manuscript. JL and SZ contributed to the data collection. All authors read and approved the final manuscript.

## Conflict of Interest

The authors declare that the research was conducted in the absence of any commercial or financial relationships that could be construed as a potential conflict of interest.

## Publisher’s Note

All claims expressed in this article are solely those of the authors and do not necessarily represent those of their affiliated organizations, or those of the publisher, the editors and the reviewers. Any product that may be evaluated in this article, or claim that may be made by its manufacturer, is not guaranteed or endorsed by the publisher.
