# Supplementary material for: GLP-1 RAs and SGLT-2 Inhibitors for Insulin Resistance in Nonalcoholic Fatty Liver Disease: Systematic Review and Network Meta-Analysis
Source: Front Endocrinol (Lausanne). 2022 Jul 13;13:923606. doi: 10.3389/fendo.2022.923606 (PMC9325993; doi:10.3389/fendo.2022.923606)
Supplement: Supplementary file 1 [file DataSheet_1.pdf]

Appendix 1 Search strategies

Detailed search strategy for EMBASE

#1'nonalcoholic fatty liver/exp OR "nonalcoholic fatty liver"
#2'sodium glucose cotransporter 2 inhibitor'lexp OR 'sodium glucose cotransporter 2 inhibitor'#1
#3'dipeptidyl peptidase iv inhibitorlexp OR 'dipeptidyl peptidase iv inhibitor"
#4'glp 1"/exp OR 'glp 1'
#5'glucagon like peptide 1"/exp OR 'glucagon like peptide 1'OR'glucagon like peptide 1 receptor agonistlexp OR'glucagon likepeptide 1 receptor agonist"
#6'peroxisome proliferator activated receptorlexp OR 'peroxisome proliferator activated receptor' OR 'peroxisome proliferator activated receptor gamma'exp OR 'peroxisome proliferator activated receptor gamma' OR 'peroxisome proliferator activatedreceptor alpha'/exp OR "peroxisome proliferator activated receptor alpha"
#7'metformin'lexp OR metformin
#8' #2 OR #3 OR #4 OR #5 OR#6 OR #7
#9'#1AND #8
#11'#9AND 'randomized controlled trial'/de

Detailed search strategy for Cochrane

#1 (NAFLD):ti,ab,kw
#2 MeSH descriptor: [Non-alcoholic Fatty Liver Disease] explode all trees
#3 #1 OR #2
#4 metformin:ti,ab,kw
#5 (sulfonylurea* or sulphonylurea*):ti,ab,kw
#6 acetohexamide:ti,ab,kw
#7 carbutamide:ti,ab,kw
#8 chlopropramide:ti,ab,kw
#9 glibenclamide:ti,ab,kw
#10 gliclazide:ti,ab,kw
#11 glimepiride:ti,ab,kw
#12 gliquidone:ti,ab,kw
#13 glyburide:ti,ab,kw
#14 glibornuride:ti,ab,kw
#15 glipizide:ti,ab,kw
#16 glycopyramide:ti,ab,kw
#17 tolazimide:ti,ab,kw
#18 (glitazone near/1 (derivative* or analogue*)):ti,ab,kw
#19 thiazolidinedione:ti,ab,kw
#20 rivoglitazone:ti,ab,kw
#21 pioglitazone:ti,ab,kw
#22 rosiglitazone:ti,ab,kw
#23 ("glucagon-like peptide 1 receptor inhibitor" or "glucagon-like peptide 1 receptor agonist" or "glucagon-like peptide 1 inhibitor" or "glucagon-like peptide 1 agonist" or "GLP-1 receptor inhibitor" or "GLP-1 receptor agonist"or "GLP-1 inhibitor" or "GLP-1 agonist"):ti,ab,kw
#24 ("glucagon-like peptide 1 receptor inhibitors" or "glucagon-like peptide 1 receptor agonists" or "glucagon-like peptide 1 inhibitors" or "glucagon-like peptide 1 agonists" or "GLP-1 receptor inhibitors" or "GLP-1 receptor agonists" or "GLP-1 inhibitors" or "GLP-1 agonists"):ti,ab,kw
#25 albiglutide:ti,ab,kw
#26 dulaglutide:ti,ab,kw
#27 exenatide:ti,ab,kw
#28 “exendin 4”:ti,ab,kw
#29 liraglutide:ti,ab,kw
#30 lixisenatide:ti,ab,kw
#31 semaglutide:ti,ab,kw
#32 taspoglutide:ti,ab,kw
#33 ("dipeptidyl-peptidase IV Inhibitor" or "dipeptidylpeptidase 4 Inhibitor" or dipeptidyl-peptidase IV Inhibitors or "dipeptidyl-peptidase 4 Inhibitors"):ti,ab,kw
#34 ((DPP4 or DPP 4 or DPP IV) next inhibitor*):ti,ab,kw

#35 alogliptin:ti,ab,kw
#36 anagliptin:ti,ab,kw
#37 gemigliptin:ti,ab,kw
#38 linagliptin:ti,ab,kw
#39 omarigliptin:ti,ab,kw
#40 saxagliptin:ti,ab,kw
#41 sitagliptin:ti,ab,kw
#42 teneligliptin:ti,ab,kw
#43 vildagliptin:ti,ab,kw
#44 evogliptin:ti,ab,kw
#45 dutogliptin:ti,ab,kw
#46 retagliptin:ti,ab,kw
#47 ("sodium glucose transporter 2 inhibitor" or "sodium glucose transporter ii inhibitor" or "SGLT 2 inhibitor" or "sodium glucose transporter 2 inhibitors" or "sodium glucose transporter ii inhibitors" or "SGLT 2 inhibitors"):ti,ab,kw
#48 ("sodium glucose cotransporter" near/3 inhibitor*):ti,ab,kw
#49 ("sodium glucose co-transporter" near/3 inhibitor*):ti,ab,kw
#50 remogliflozin:ti,ab,kw
#51 sergliflozin:ti,ab,kw
#52 tofogliflozin:ti,ab,kw
#53 bexagliflozin:ti,ab,kw
#54 licogliflozin:ti,ab,kw
#55 luseogliflozin:ti,ab,kw
#56 canagliflozin:ti,ab,kw
#57 dapagliflozin:ti,ab,kw
#58 empagliflozin:ti,ab,kw
#59 ertugliflozin:ti,ab,kw
#60 henagliflozin:ti,ab,kw
#61 ipragliflozin:ti,ab,kw
#62 sotagliflozin:ti,ab,kw
#63 sotagliflozin:ti,ab,kw
#64 OR#5-#63
#65 #3 OR #64

Detailed search strategy for PubMed.

Aa No.	Search item
#1	“Non-alcoholic Fatty Liver Disease” [Mesh Terms]
#2	Steatohepatitis, Nonalcoholic [Title/Abstract] OR Steatohepatitides, Nonalcoholic [Title/Abstract] OR Nonalcoholic Steatohepatitides [Title/Abstract] OR Nonalcoholic Steatohepatitis [Title/Abstract] OR Nonalcoholic Fatty Livers [Title/Abstract] OR Nonalcoholic Fatty Liver [Title/Abstract] OR Livers, Nonalcoholic Fatty [Title/Abstract] OR Liver, Nonalcoholic Fatty [Title/Abstract] OR Fatty Livers, Nonalcoholic [Title/Abstract])) OR Fatty Liver, Nonalcoholic [Title/Abstract] OR Nonalcoholic Fatty Liver Disease [Title/Abstract] OR NAFLD [Title/Abstract] OR Non alcoholic Fatty Liver Disease [Title/Abstract]
#3	#1 OR #2
#3	“antidiabetic” [Mesh Terms]
#5	("Elafibranor"[Title/Abstract] OR "lanifibranor"[Title/Abstract] OR "Liraglutide" [Title/Abstract] OR "semaglutide"[Title/Abstract] OR "pioglitazone"[Title/Abstract] OR "Empagliflozin"[Title/Abstract] OR "Canagliflozin"[Title/Abstract] OR "Dapagliflozin"[Title/Abstract] OR "Empagliflozin"[Title/Abstract] OR "Ipragliflozin" [Title/Abstract] OR "Luseogliflozin"[Title/Abstract] OR "Tofogliflozin" [Title/Abstract] OR "metformin"[Title/Abstract] OR "PPAR"[Title/Abstract] OR "SGLT"[Title/Abstract] OR "DPP-4"[Title/Abstract] OR "GLP-1"[Title/Abstract])
#6	#4 AND #5
#7	Randomized controlled trial [Publication Type] AND Controlled clinical trial [Publication Type]
#8	Randomized [Title/Abstract] OR random allocation [Title/Abstract
#9	#7 OR #8
#10	#3 AND #6 AND #9

Appendix 2 Included studies

STUDY ID	Trial registration	TIME	Country	Randomised treatments	DOSE	N	SEX (N of men)	AGE (Mean±SD)	FPG (mmol/L)	T2DM or not	Combined therapy	Lifestyle	
Aithal-2008	ISRCTN: 10319160	48W	UK	placebo	/	37	19	51.4± 35.4	5.6±0.9	×	NA	√	Patients were seen by a dietician and were instructed to reduce their calorie intake by 500 Kcal/day as well as to perform modest exercise (such as walking, swimming, gardening, and so forth) regularly for 30 – 40 minutes per day at least 5 days per week.
				TZD	30 mg/day	37	26	50.2± 33.1	5.7±1.6				
Anushiravani-2019	ISRCTN: 10319160	12W	Iran	placbo	/	30	77	47.0±9.1	NA	×	NA	√	500 kcal/day deficit from weight-maintaining caloric intake
				metformin	500 mg/day	30			NA				
				TZD	15 mg/day	30			NA				
Aso-2019	UMIN000022155	24 W	Japan	SGLT-2	5 mg/d	33	19	56.2 ± 11.5	7.66±3.01	√	standard treatment		NA
				standard treatment	/	24	15	57.1 ± 13.8	7.59±2.29				
Balas-2007	NA	24W	USA&Italy	placbo	/	14	5	48.4 ± 3.1	6.22±0.44	√	NA	√	reduce their caloric intake by ~500 kcal/day
				TZD	30 mg/day<br>titrated after 2months to 45mg/day	21	11	51 ± 1.6	5.39±0.17				
Belfort-2006	NCT00227110	24W	USA	placbo	/	21	7	51±10	6.39±1.56	impaired glucose tolerance or type 2 diabetes	NA	√	hypocaloric diet (a reduction of 500 kcal per day in relation to the calculated daily intake required to maintain body weight)
				TZD	45 mg daily	26	14	51±7	6.61±1.94				
Bell-2012	NCT00063622	96W	USA	Placebo	/	82	NA	NA	NA	×	NA		NA
				TZD	30mg daily	80	NA	NA	NA				
Chehrehgosha-2021	IRCT20190122042450N3	24W	Iran	placebo	/	37	14	51.8 ± 7.8	8.68±1.89	√	stain	√	Participants were asked to perform moderate intensity physical activity based on a metabolic<br>equivalent task (METS) at least 3 times a week,<br>and they were encouraged to follow the recommendation<br>at least 45 min without interruption during the study period.<br>Participants were given standard dietary advice as well.
				SGLT-2	30mg	35	15	50.5 ± 8.4	8.31±2.34				
				TZD	10mg	34	17	52.5 ± 7.9	9.41±2.68				
Cusi-2016	NCT00994682	72M	USA	Placebo	/	51	35	49±11	6.78±1.51	√	NA	√	hypocaloric diet(500 – kcal/d deficit from weight maintaining caloric intake)
				TZD	30 mg/d<br>(titrated after 2 months to 45 mg/d)	50	36	52±10	6.94±1.51				
Feng-2017	NCT03068065	24W	China	GLP-1	first week:0.6 mg/day<br>second week:1.2 mg/day<br>third week:1.8mg/day	29	21	46.79 ± 1.80	8.8±2.4	√	NA	√	proper diet and exercise
				sulfonylurea	30 mg	29	20	48.07 ± 2.34	9±1.6				
				Metformin	first week:250 mg thrice daily<br>second week:500 mg thrice daily<br>third week:1000 mg twice daily	29	19	46.31 ± 2.29	8±1.9				
Guo-2020	no. ChiCTR2000035091	26W	China	GLP-1	starting dose of 0.6 mg 1/ day<br>increased by weekly forced titration to 1.8mg	31	18	52.0 ± 8.7	7.1±1.2	√	Metformin at<br>a constant dose.	√	exercise,<br>weight loss and diet adjustment.
				Placebo	/	30	16	53.1 ± 6.3	7.3±1.6				
HAUKELAND-2009	NCT00303537	24W	Norway	Placebo	/	24	16	49.9 ± 12.8	5.7±1	√	NA	√	physical activity at least 30 min daily and a diet low in fat, particularly saturated fat, and refined carbohydrates.
				Metformin	started with 500 mg<br>increased every week until a maximal daily dose of 2500 mg or 3000 mg	20	16	44.3 ± 9.0	5.5±0.8				
Ito-2017	UMIN000022651	24W	Japan	TZD	15–30 mg	34	18	59.1 ± 9.8	9.41±2.82	√	Medications:<br>Metformin;DPP-4 inhibitor;<br>Sulfonylurea;Insulin;<br>ARB or ACE inhibitor;<br>Statin	√	received diet and exercise counseling
				SGLT2	50 mg	32	14	57.3 ± 12.1	8.89±2.15				
Joy-2017	NCT01260246	24W	UK	placebo	/	6	2	54.7 ± 9.8	NA	√			NA
				DPP-4	100 mg daily	6	3	56.7 ± 9.9	NA				
Kinoshita-2020	UMIN 000021291	28W	Japan	TZD	7.5–15 mg/day	33	15	59±1.9	7.61±0.22	√	Medications:<br>Glinide;DPP-4 inhibitor;<br>Metformin;GLP-1 RA;<br>α-GI; Ursodeoxycholic acid;<br>ARB or ACE inhibitor;<br>Statin;Fibrate;EPA		NA
				Sus	0.5–1 mg/day	33	15	58.0±2.3	8.22±0.34				
				SGLT-2	5 mg/day	32	15	58.7±1.6	8.06±0.38				
Kuchay-2018	NA	20W	India	standard treatment	/	20	NA	NA	9.78±3.17	√	NA		NA
				SGLT-2	10 mg/d	22	NA	NA	9.61±2.44				
Nadeau-2009	NA	24W	USA	Placebo	/	13	5	NA	4.93±0.14	×	NA		NA
				Metformin	tartedinitially 500 mg once daily<br>increased to 500 mg twice daily at 1 month<br>then to 850 mg twice daily at 2 months	37	12	NA	5.06±0.12				
Omer-2010	NA	48W	Turkey	Metformin	1700 mg/d	22	15	48.0 ± 9.8	6.59±1.28	√	NA	√	exercise， dietary counseling
				SGLT2	4 mg/d	20	9	49.3 ± 6	7.02±1.44				
Shargorodsky-2012	NCT01084486	48W	Israel	Metformin	850– 1700 mg/day	32	17	51.9 ± 10.9	7.32±2.85	NA	Concomitant medication:<br>Antidiabetic treatment；<br>statins； ACEIs/ARBs；<br>Diuretics； B-blockers；<br>CCB-blockers； Aspirin		NA
				placebo	/	31	14	55.2 ± 14.0	5.46±0.82				
Shibuya-2018	no. UMIN000016090	24W	Japan	SGLT2	2.5 mg	16	10	53.5± 12.1	7.06±0.28	√	NA	×	Lifestyle modification was not suggested during the study period
				Metformin	1500 mg	16	8	53.6± 10.6	8.17±0.49				
Sofer-2011	NCT01084486	16W	Israel	Metformin	850-1700 mg/d	32	17	51.9 ± 10.9	7.5±2.93	NA	Concomitant medication:<br>Antidiabetic treatment；<br>statins ;ACEIs/ARBs；<br>Diuretics； B-blockers；<br>CCB-blockers； Aspirin		NA
				placebo	/	31	14	55.2 ± 14.0	5.45±0.88				
Taheri-2020	IRCT20190122042450N1	24W	Iran	SGLT2	10 mg/day	43	28	43.8± 9.7	5.22±0.51	×	NA	√	Participants were encouraged to perform moderate intensity physical activity in the form of 3–6 times the metabolic equivalent task (METs)for more than three times a week, and they were encouraged to follow the recommendation at least 45 min without interruption during the study period. Participants were given standard dietary advice as well.
				placebo	/	47	22	44.1± 9.3	5.08±0.43				
Tobita-2021	UMIN000027304	12W	Japan	SGLT-2	5 mg/d	12	8	51.6 ± 12.6	5.57±0.38	×	NA		NA
				DPP-4	20Mg/d	10	7	41.8 ± 16.5	5.29±0.37				
Yan-2019	NCT02147925	26W	China	GLP-1	1.8 mg /d	24	17	43.1 ± 9.7	8.6±2.8	√	metformin	√	dietary and exercise
				DPP-4	100 mg once daily	27	21	45.7 ± 9.2	8.4±2.5				
Yoneda-2020	NA	24W	Japan	TZD	15-30 mg	19	8	58.8±8.1	8.01±2.28	√	Medication of diabetes<br>(Metformin;DPP-4;<br>Sulfonylurea;Alpha- G)		NA
				SGLT-2	20 mg	21	13	58.4±12.2	8.03±2.79				
Zhang-2020	NA	24W	China	GLP-1	first week:0.6 mg/day<br>second week:1.2mg/day	30	13	50.2 ± 11.5	8.9±2.9	√	Metformin<br>500 mg TID		NA
				TZD	first week:15 mg/day<br>second week:30 mg/day	30	15	51 .5 ± 12.1	8.7±3.3				

Appendix 3 Risk of bias in included studies

Study	Bais	Evaluation	Evidence
Aithal-2008	Random sequence generation(selection bias)	LOW	Randomization was performed via the arand computer program (Pharmacy department, University Hospitals NHS Trust, Nottingham, UK)
	Allocation concealiment (selectionbias)	UN	NA
	Blinding of participants and personnel(performance bias)	LOW	All clinical and laboratory data were collected in a double-blind manner.
	Blinding of outcome assessment(detection bias)	LOW	Liver biopsy specimens were graded and staged according to the NASH histologic scoring
	incomplete outcome data (attritionbias)	LOW	37/37,37/37
	Selective reporting (reporting bias)	UN	NA
	other bias	Hight	Takeda Pharmaceuticals UK provided the pioglitazone and placebo tablets for this
Anushiravani-2019	Random sequence generation(selection bias)	LOW	These patients were selected randomly using a table of random permutations of 20
	Allocation concealiment (selectionbias)	LOW	Study pills were allocated in separate packs blinded and labeled using a four-digit code.
	Blinding of participants and personnel(performance bias)	LOW	Apart from the project coordinator, the patients, attending physicians, staff involved in the
	Blinding of outcome assessment(detection bias)	LOW	Apart from the project coordinator, the patients, attending physicians, staff involved in the
	incomplete outcome data (attritionbias)	LOW	30/30,30/30
	Selective reporting (reporting bias)	LOW	https://www.irct.ir/trial/6761
	other bias	LOW	There are no conflicts of interest.
Aso-2019	Random sequence generation(selection bias)	UN	NA
	Allocation concealiment (selectionbias)	Hight	open-label
	Blinding of participants and personnel(performance bias)	Hight	open-label
	Blinding of outcome assessment(detection bias)	LOW	objective indicator
	incomplete outcome data (attritionbias)	LOW	33/33,24/24
	Selective reporting (reporting bias)	LOW	https://upload.umin.ac.jp/cgi-open-bin/ctr_e/ctr_view.cgi?recptno=R000025530
	other bias	LOW	Funding information:Ono Medical Research Foundation
Balas-2007	Random sequence generation(selection bias)	LOW	Randomization was computer-generated by the research pharmacy and blinded to investigators.
	Allocation concealiment (selectionbias)	LOW	computer-generated by the research pharmacy
	Blinding of participants and personnel(performance bias)	UN	blinded to investigators
	Blinding of outcome assessment(detection bias)	LOW	objective indicator
	incomplete outcome data (attritionbias)	UN	NA
	Selective reporting (reporting bias)	UN	NA
	other bias	Hight	they received partial funding from the manufacturers to carry out their research (Takeda Pharmaceuticals North America).
Belfort-2006	Random sequence generation(selection bias)	LOW	Randomization was computer-generated by the research pharmacy.
	Allocation concealiment (selectionbias)	UN	/
	Blinding of participants and personnel(performance bias)	LOW	The investigators were unaware of the treatment assignments.
	Blinding of outcome assessment(detection bias)	LOW	objective indicator
	incomplete outcome data (attritionbias)	LOW	21/25,26/30
	Selective reporting (reporting bias)	UN	NA
	other bias	Hight	Takeda Pharma-ceuticals
Bell-2012	Random sequence generation(selection bias)	UN	NA
	Allocation concealiment (selectionbias)	UN	NA
	Blinding of participants and personnel(performance bias)	UN	NA
	Blinding of outcome assessment(detection bias)	LOW	objective indicator
	incomplete outcome data (attritionbias)	LOW	74/82,73/84.69/80
	Selective reporting (reporting bias)	LOW	Pioglitazone vs Vitamin E vs Placebo for Treatment of Non-Diabetic Patients With
	other bias	UN	NA
Chehrehgosha-2021	Random sequence generation(selection bias)	LOW	computer-generated randomization sequence
	Allocation concealiment (selectionbias)	LOW	allocation to the treatment arms using a computer-generated randomization sequence.
	Blinding of participants and personnel(performance bias)	LOW	double-blind, placebo-controlled
	Blinding of outcome assessment(detection bias)	LOW	objective indicator
	incomplete outcome data (attritionbias)	LOW	37/37,35/35,34/34
	Selective reporting (reporting bias)	LOW	IRCT | Comparison of the effect of empagliflozin and pioglitazone and placebo on
	other bias	LOW	Abidi Pharmaceuticals supplied empagliflozin, pioglitazone, and placebo and had no other
Cusi-2016	Random sequence generation(selection bias)	UN	NA
	Allocation concealiment (selectionbias)	UN	NA
	Blinding of participants and personnel(performance bias)	LOW	Placebo is an oral tablet identical to pioglitazone
	Blinding of outcome assessment(detection bias)	LOW	objective indicator
	incomplete outcome data (attritionbias)	LOW	51/51,50/50
	Selective reporting (reporting bias)	LOW	University of Texas H.S.C. San Antonio Pioglitazone in Non-Alcoholic Steatohepatitis
	other bias	LOW	By the Burroughs Wellcome Fund (1006762.01 [Dr. Cusi]) and the American Diabetes
Feng-2017	Random sequence generation(selection bias)	LOW	Use computergenerated random numbers
	Allocation concealiment (selectionbias)	LOW	Use computergenerated random numbers
	Blinding of participants and personnel(performance bias)	Hight	open-label
	Blinding of outcome assessment(detection bias)	LOW	objective indicator
	incomplete outcome data (attritionbias)	LOW	29/29,29/29,29/29
	Selective reporting (reporting bias)	LOW	Antidiabetic Effects on Intrahepatic Fat - Full Text View - ClinicalTrials.gov
	other bias	LOW	This work was supported by grants from the National Natural Science Foundation of China.
Guo-2020	Random sequence generation(selection bias)	LOW	Patients were randomized by the trial pharmacist using computer-generated numbers.
	Allocation concealiment (selectionbias)	LOW	Patients were randomized by the trial pharmacist using computer-generated numbers.
	Blinding of participants and personnel(performance bias)	Hight	This was not a blinded study.
	Blinding of outcome assessment(detection bias)	LOW	objective indicator
	incomplete outcome data (attritionbias)	LOW	30/32,31/32,30/32
	Selective reporting (reporting bias)	LOW	中国临床试验注册中心 - 世界卫生组织国际临床试验注册平台一级注册机构 (chictr.org.cn)
	other bias	LOW	The project was funded by the Natural Science Foundation of Fujian Province (2017Y0068) and 900 Hospital of the Joint Logistics Team Internal Hospital Project (2019L19).
HAUKELAND-2009	Random sequence generation(selection bias)	LOW	Patients included in the study were randomized to treatment with metformin for 6 months
	Allocation concealiment (selectionbias)	LOW	Patients included in the study were randomized to treatment with metformin for 6 months
	Blinding of participants and personnel(performance bias)	LOW	The allocation code was blinded to patients and investigators until all patients had
	Blinding of outcome assessment(detection bias)	LOW	objective indicator
	incomplete outcome data (attritionbias)	Hight	16/20,24/24
	Selective reporting (reporting bias)	LOW	https://clinicaltrials.gov/ct2/show/NCT00303537?term=NCT00303537&draw=2&rank=1
	other bias	LOW	The authors report no conflicts of interest. The authors alone are responsible for the content and
Ito-2017	Random sequence generation(selection bias)	LOW	computer-generated randomization sequence
	Allocation concealiment (selectionbias)	LOW	patients were randomly assigned (1:1) using a computer-generated randomization sequence
	Blinding of participants and personnel(performance bias)	Hight	open-label
	Blinding of outcome assessment(detection bias)	LOW	objective indicator
	incomplete outcome data (attritionbias)	LOW	30/34,31/32
	Selective reporting (reporting bias)	LOW	https://upload.umin.ac.jp/cgi-open-bin/ctr_e/ctr_view.cgi?recptno=R000026084
	other bias	LOW	Sponsor:Ogawa Red Cross Hospital
Joy-2017	Random sequence generation(selection bias)	LOW	The St. Joseph's Hospital clinical trial pharmacy team randomized patients into
	Allocation concealiment (selectionbias)	LOW	The St. Joseph's Hospital clinical trial pharmacy team randomized patients into
	Blinding of participants and personnel(performance bias)	LOW	Blinding and allocation concealment was maintained by use of identicallooking bottles and
	Blinding of outcome assessment(detection bias)	LOW	objective indicator
	incomplete outcome data (attritionbias)	LOW	5/6,5/6
	Selective reporting (reporting bias)	LOW	https://clinicaltrials.gov/ct2/show/NCT01260246?term=NCT01260246&draw=2&rank=1
	other bias	LOW	Sponsor:Lawson Health Research Institute
Kinoshita-2020	Random sequence generation(selection bias)	LOW	A random allocation sequence was generated by a clinical epidemiologist who was not aware of this study protocol using a computer software program
	Allocation concealiment (selectionbias)	LOW	The enrollment of patients and their assignment into the three groups were carried out by
	Blinding of participants and personnel(performance bias)	Hight	open-label
	Blinding of outcome assessment(detection bias)	LOW	objective indicator
	incomplete outcome data (attritionbias)	Hight	33/36,33/34,32/40
	Selective reporting (reporting bias)	LOW	https://rctportal.niph.go.jp/detail/um?trial_id=UMIN000021291
	other bias	LOW	Research Project Grant 29G-002 from Kawasaki Medical School.
Kuchay-2018	Random sequence generation(selection bias)	LOW	computer-generated numbers
	Allocation concealiment (selectionbias)	UN	NA
	Blinding of participants and personnel(performance bias)	Hight	open-label
	Blinding of outcome assessment(detection bias)	LOW	objective indicator
	incomplete outcome data (attritionbias)	Hight	22/25(3 Discontinued study:1 Balanoposthitis ,1 Non specific fatigue ,1 Significant arthralgia),
	Selective reporting (reporting bias)	LOW	https://clinicaltrials.gov/ct2/show/NCT02686476?term=NCT02686476&draw=2&rank=1
	other bias	LOW	The current study was supported by an investigator-initiated study grant from the Endocrine and Diabetes Foundation, India (to M.S.K.). The funding agency did not play a role in the design and conduct of the study; collection, management, analysis, or interpretation of the
Nadeau-2009	Random sequence generation(selection bias)	UN	Subjects were randomized by a research pharmacist
	Allocation concealiment (selectionbias)	UN	Subjects were randomized by a research pharmacist
	Blinding of participants and personnel(performance bias)	LOW	double-blind
	Blinding of outcome assessment(detection bias)	LOW	objective indicator
	incomplete outcome data (attritionbias)	LOW	10/13,28/37
	Selective reporting (reporting bias)	UN	NA
	other bias	LOW	This research was supported by grant number M01 RR00069, General Clinical Research Centers Program, National Centers for Research Resources, NIH and NIH/NCRR 1K23 RR020038
Omer-2010	Random sequence generation(selection bias)	UN	NA
	Allocation concealiment (selectionbias)	UN	NA
	Blinding of participants and personnel(performance bias)	Hight	open-label
	Blinding of outcome assessment(detection bias)	LOW	objective indicator
	incomplete outcome data (attritionbias)	UN	NA
	Selective reporting (reporting bias)	UN	NA
	other bias	LOW	Departments of Internal Medicine, Endocrinology, Gastroenterology and Pathology, Ege University Medical School, Bornova, Izmir, Turkey
Shargorodsky-2012	Random sequence generation(selection bias)	UN	NA
	Allocation concealiment (selectionbias)	UN	NA
	Blinding of participants and personnel(performance bias)	UN	placebo controlled
	Blinding of outcome assessment(detection bias)	LOW	objective indicator
	incomplete outcome data (attritionbias)	UN	NA
	Selective reporting (reporting bias)	UN	Effect of Treatment With Insulin Sensitizer on Arterial Properties, Metabolic Parameters and Liver Function in Patients With Nonalcoholic Fatty Liver Disease - Full Text View -
	other bias	LOW	The corresponding author and all of the authors have no conflicts of interest or financial or other contractual agreements that might cause conflicts of interest.
Shibuya-2018	Random sequence generation(selection bias)	LOW	Participants were randomly assigned by the envelope method
	Allocation concealiment (selectionbias)	LOW	Participants were randomly assigned by the envelope method
	Blinding of participants and personnel(performance bias)	Hight	open-label
	Blinding of outcome assessment(detection bias)	LOW	objective indicator
	incomplete outcome data (attritionbias)	LOW	16/16,16/16
	Selective reporting (reporting bias)	LOW	脂肪肝合併２型糖尿病患者におけるルセオグリフロジン の影響に関する無作為化比較試験
	other bias	LOW	The authors declare no potential conflict of interest concerning this article.
Sofer-2011	Random sequence generation(selection bias)	UN	NA
	Allocation concealiment (selectionbias)	UN	NA
	Blinding of participants and personnel(performance bias)	LOW	matching placebo capsules
	Blinding of outcome assessment(detection bias)	LOW	objective indicator
	incomplete outcome data (attritionbias)	LOW	27/32,25/31
	Selective reporting (reporting bias)	UN	https://clinicaltrials.gov/ct2/show/NCT01084486?term=NCT01084486&draw=2&rank=1
	other bias	LOW	Sponsor:Wolfson Medical Center
Taheri-2020	Random sequence generation(selection bias)	UN	NA
	Allocation concealiment (selectionbias)	LOW	Eligible subjects were randomly assigned in the two study arms using a block randomization method with block size of 4.
	Blinding of participants and personnel(performance bias)	LOW	Empagliflozin and placebo were quite similar with identical size, shape, color, and packaging.
	Blinding of outcome assessment(detection bias)	LOW	objective indicator
	incomplete outcome data (attritionbias)	Hight	43/50,47/50
	Selective reporting (reporting bias)	LOW	IRCT | Comparison of the effect of Empagliflozin with Placebo on liver steatosis change in fibroscan in non diabetic patients with nonalcoholic fatty liver disease
	other bias	LOW	The role of Abidi Pharmaceuticals was limited to supply of the medicines, empagliflozin and placebo. Data management was performed by the Institute of Endocrinology and Metabolism monitoring committee that was blinded to the study arms. No funding or sponsorship was received for this study or publication of this article.
Tobita-2021	Random sequence generation(selection bias)	LOW	Randomized patients using computer-generated numbers.
	Allocation concealiment (selectionbias)	LOW	Randomized patients using computer-generated numbers.
	Blinding of participants and personnel(performance bias)	LOW	Blinding and allocation concealment were maintained by use of identical-looking bottles and
	Blinding of outcome assessment(detection bias)	LOW	objective indicator
	incomplete outcome data (attritionbias)	LOW	12/13(Excluded one patientfor ALT<31 u/L),10/13(Excluded 3 patients forPG >200 mg/dl at 120 min in 75 g OGTT)
	Selective reporting (reporting bias)	LOW	非アルコール性脂肪性肝疾患に対する糖尿病治療薬（SGLT2阻害薬・DPP4阻害薬）の効果に 関する検討：無作為化並行群間二重盲検比較試験 | 関連する治験情報【臨床研究情報ポ
	other bias	LOW	The authors have no sources of funding, grant support and financial disclosure of this study.
Yan-2019	Random sequence generation(selection bias)	LOW	A randomization list was generated using Statistical Analysis System (SAS Institute, Inc., Cary, NC)
	Allocation concealiment (selectionbias)	LOW	Patients were allocated using a secure Oracle-based interactive web response system (Jiaxing Taimei Medical Technology, Shanghai, China) in accordance with the sequence from the randomization list.
	Blinding of participants and personnel(performance bias)	Hight	open-label
	Blinding of outcome assessment(detection bias)	LOW	objective indicator
	incomplete outcome data (attritionbias)	Hight	18/24(four lost to follow-up, one for protocol violations, and one for AEs),26/27(one patient in
	Selective reporting (reporting bias)	LOW	https://clinicaltrials.gov/ct2/show/NCT02147925?term=NCT02147925&draw=2&rank=1
	other bias	LOW	Novo Nordisk neither influenced the content of this publication nor was it involved in the study
Yoneda-2020	Random sequence generation(selection bias)	LOW	Randomization was performed using a computer- generated, centrally administered procedure
	Allocation concealiment (selectionbias)	LOW	Patients were allocated to each treatment group through the central registration system
	Blinding of participants and personnel(performance bias)	Hight	open-label
	Blinding of outcome assessment(detection bias)	LOW	objective indicator
	incomplete outcome data (attritionbias)	LOW	17/19(2 discontinued the treatment :1 due to adverse event (severe edema),1 due to incidental
	Selective reporting (reporting bias)	LOW	糖尿病を合併した非アルコール性脂肪性肝疾患（NAFLD）患者におけるトホグリフロジンの肝脂肪化改善効果をピオグリタゾンと比較する非盲検ランダム化探索的試験 | 関連する治験情報【臨床研究情報ポータルサイト】 (niph.go.jp)
	other bias	LOW	The present clinical trial was conducted at the Yokohama City University (as the sponsor) and
Zhang-2020	Random sequence generation(selection bias)	LOW	computer-generated sequence
	Allocation concealiment (selectionbias)	UN	NA
	Blinding of participants and personnel(performance bias)	Hight	open-label
	Blinding of outcome assessment(detection bias)	LOW	objective indicator
	incomplete outcome data (attritionbias)	UN	NA
	Selective reporting (reporting bias)	UN	NA
	other bias	LOW	This work was supported by the Yantai Affiliated Hospital of Binzhou Medical University

Appendix 4   Network plots for each outcome

Fig.s1 Network plot for HOMA-IR

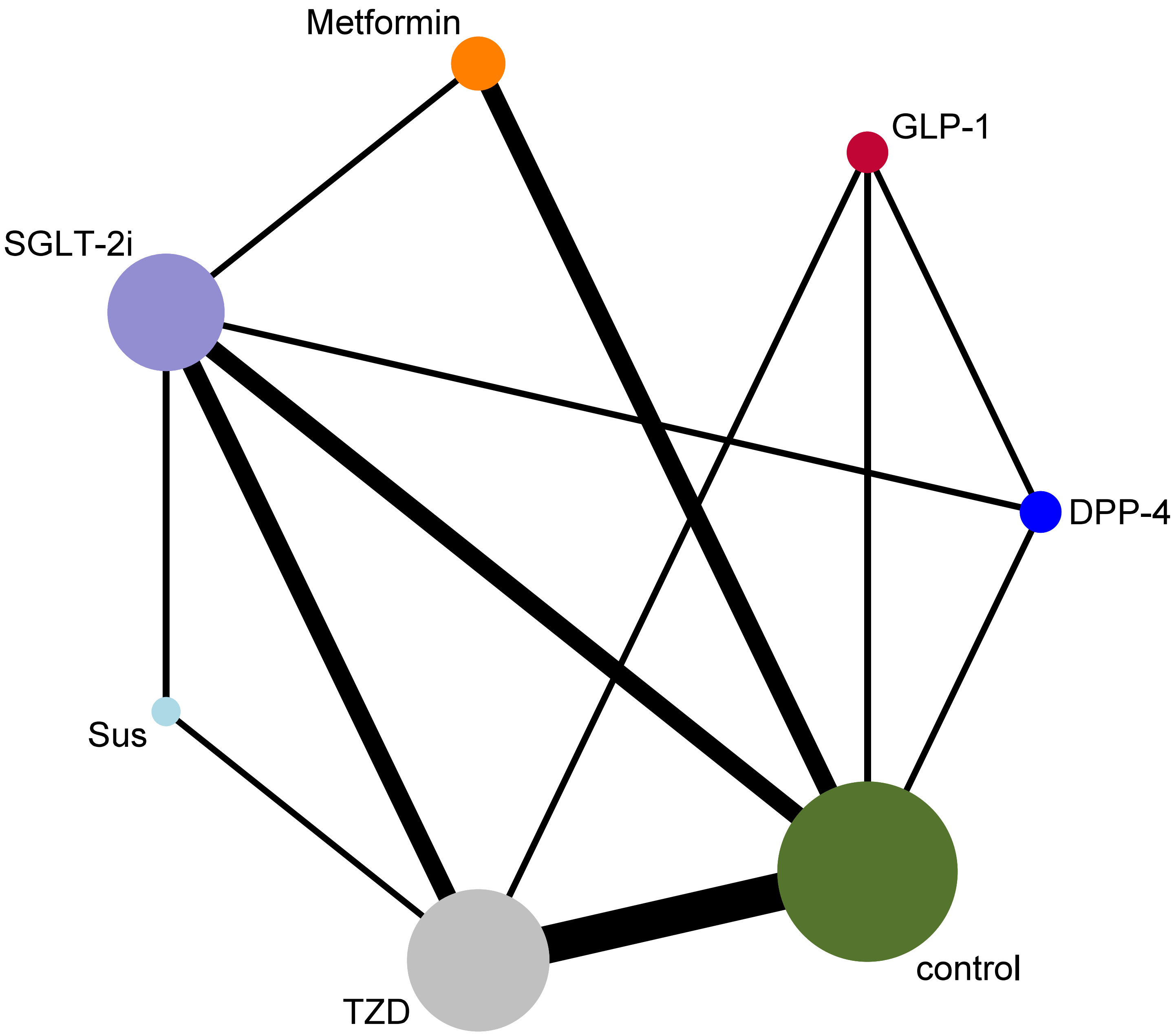

Fig.s2 Network plot for VAT

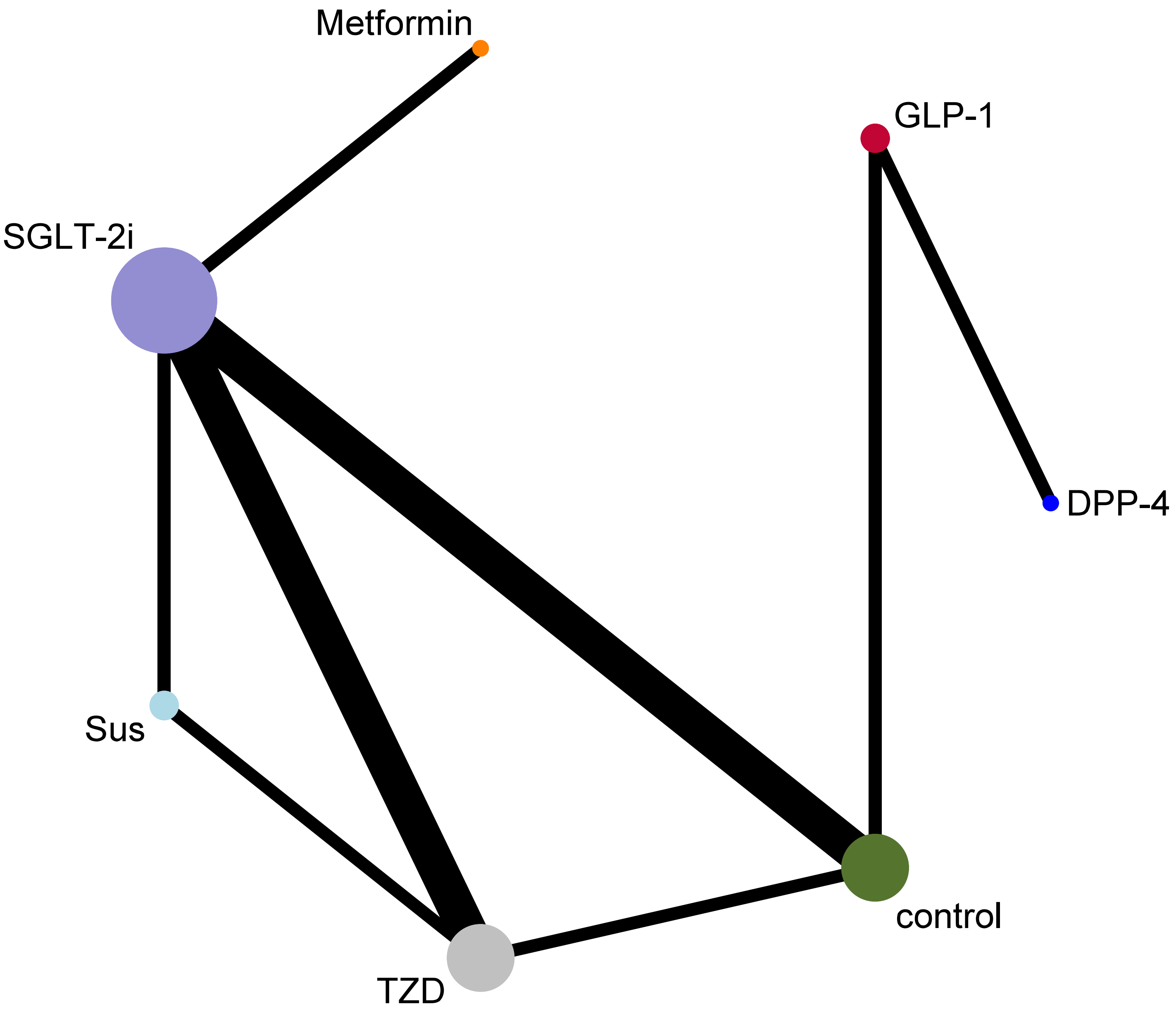

Fig.s3 Network plot for SAT

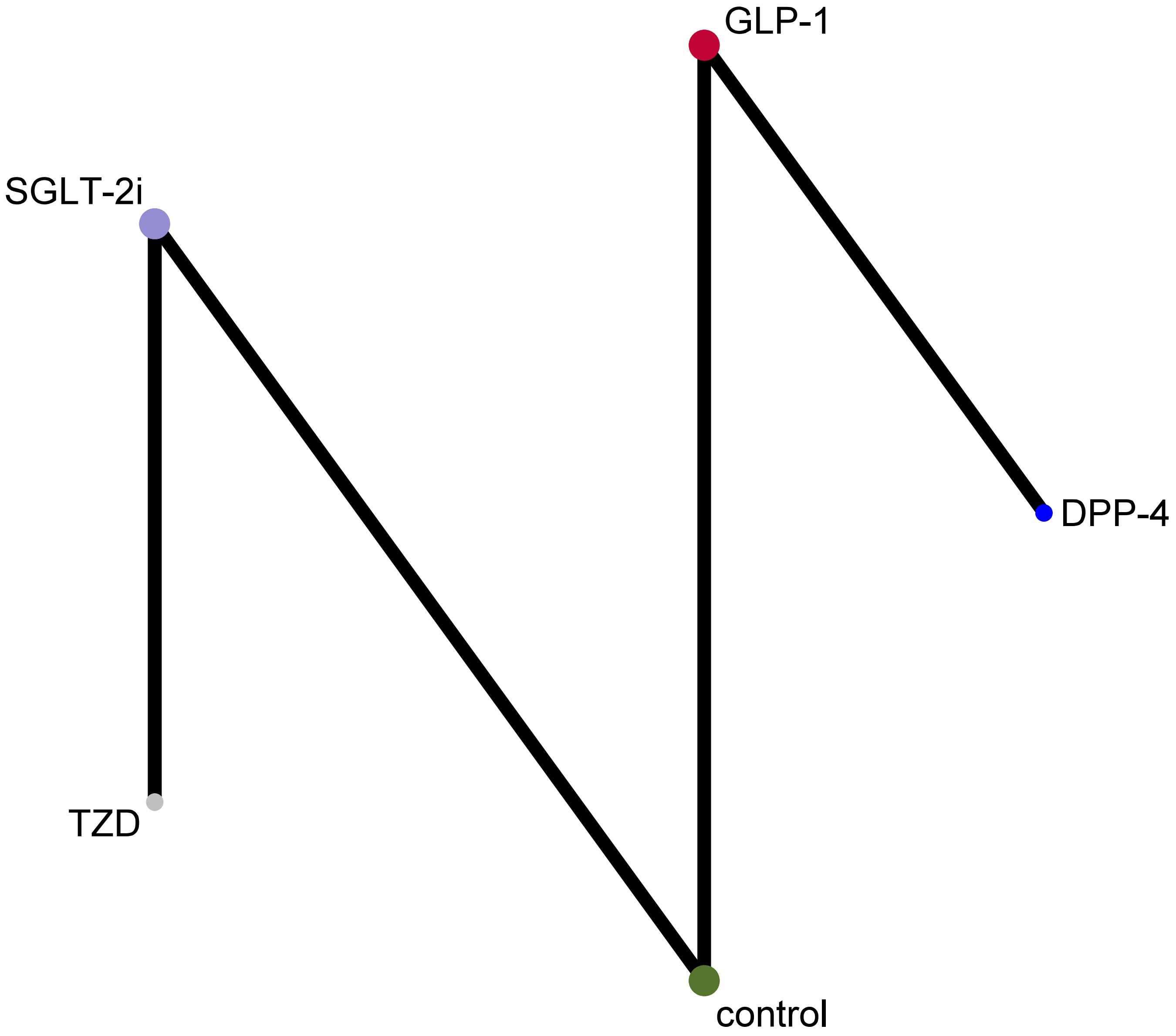

Fig.s4 Network plot for BMI

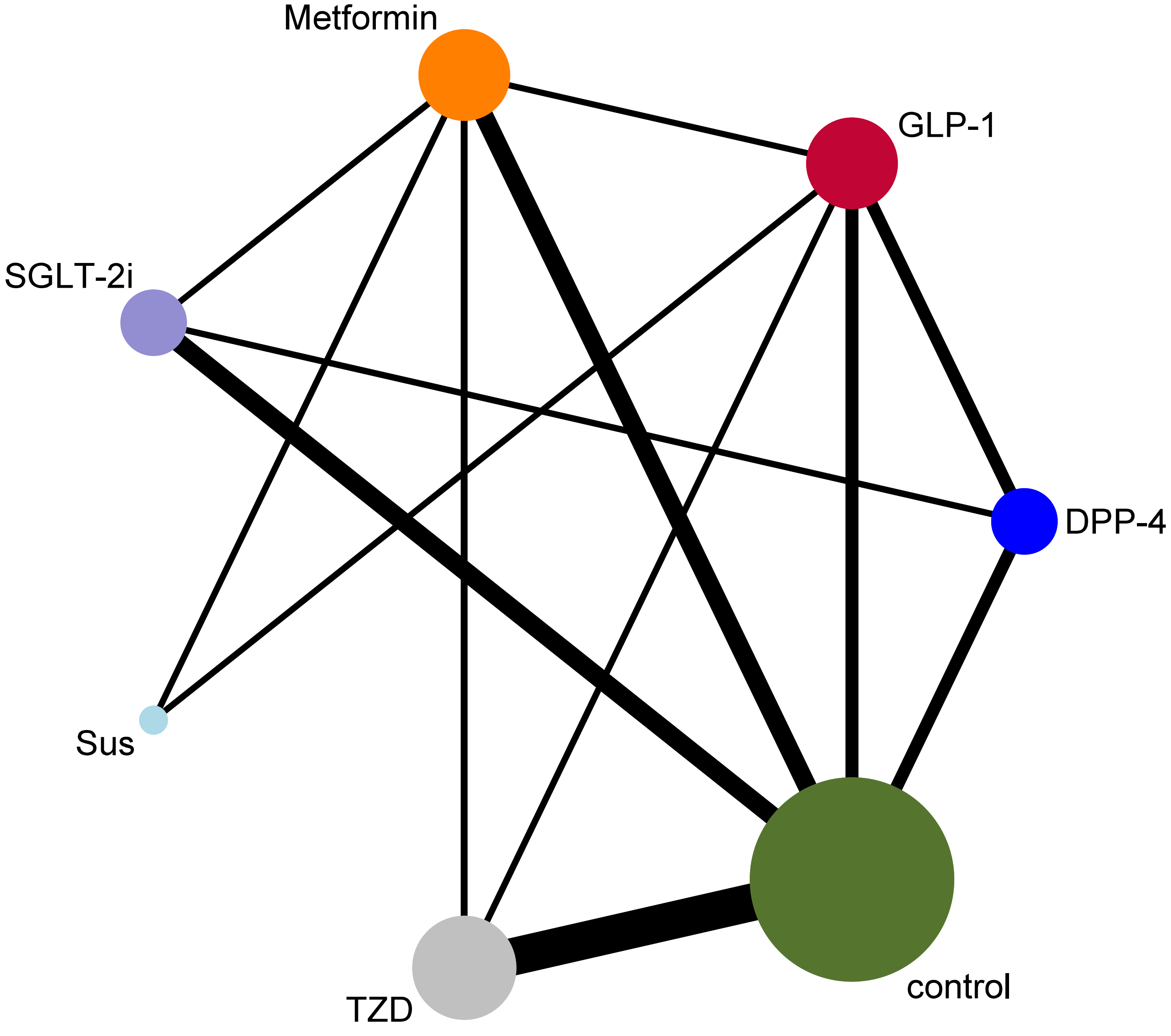

Fig.s5 Network plot for Weight

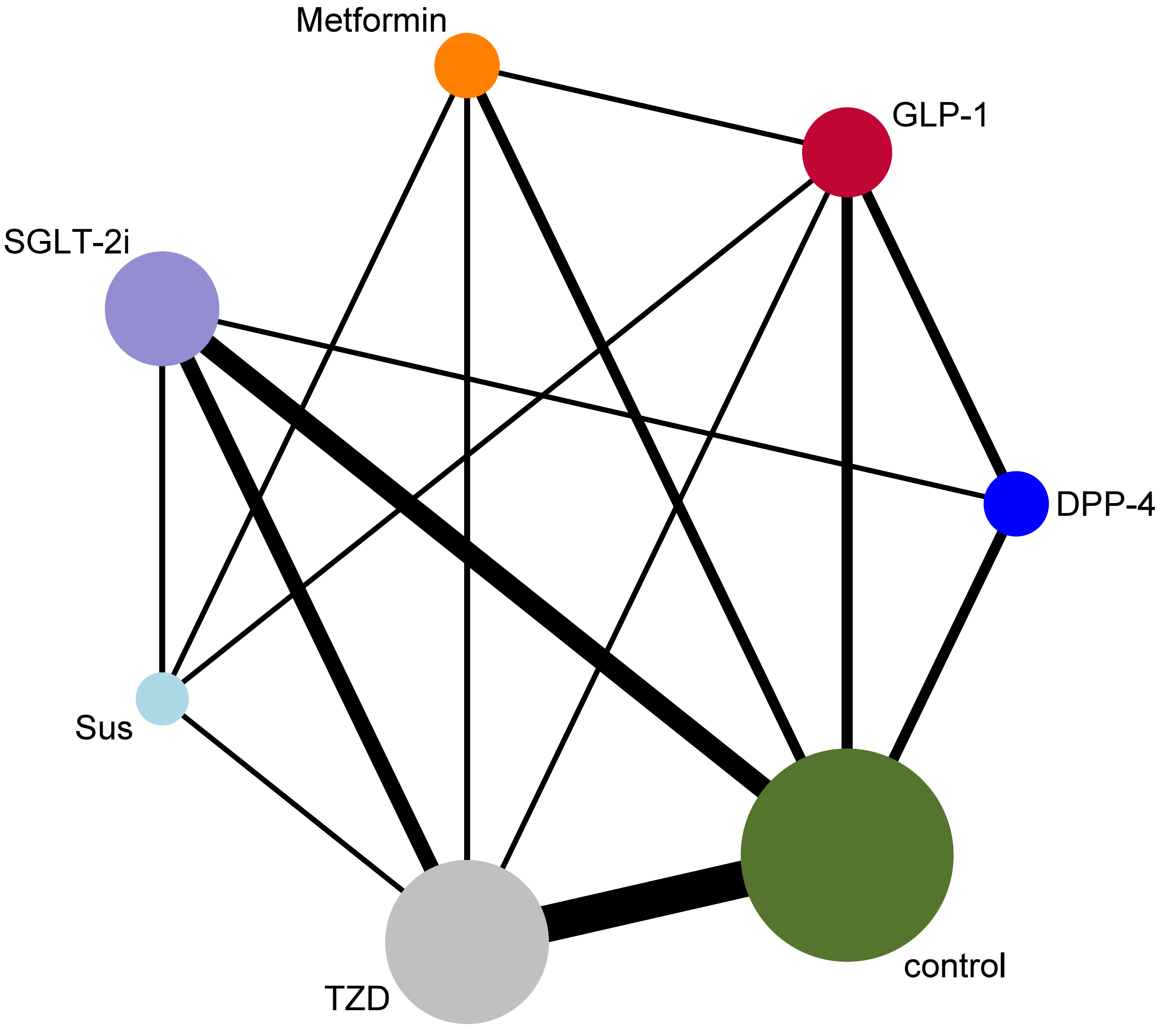

Fig.s6 Network plot for Leptin

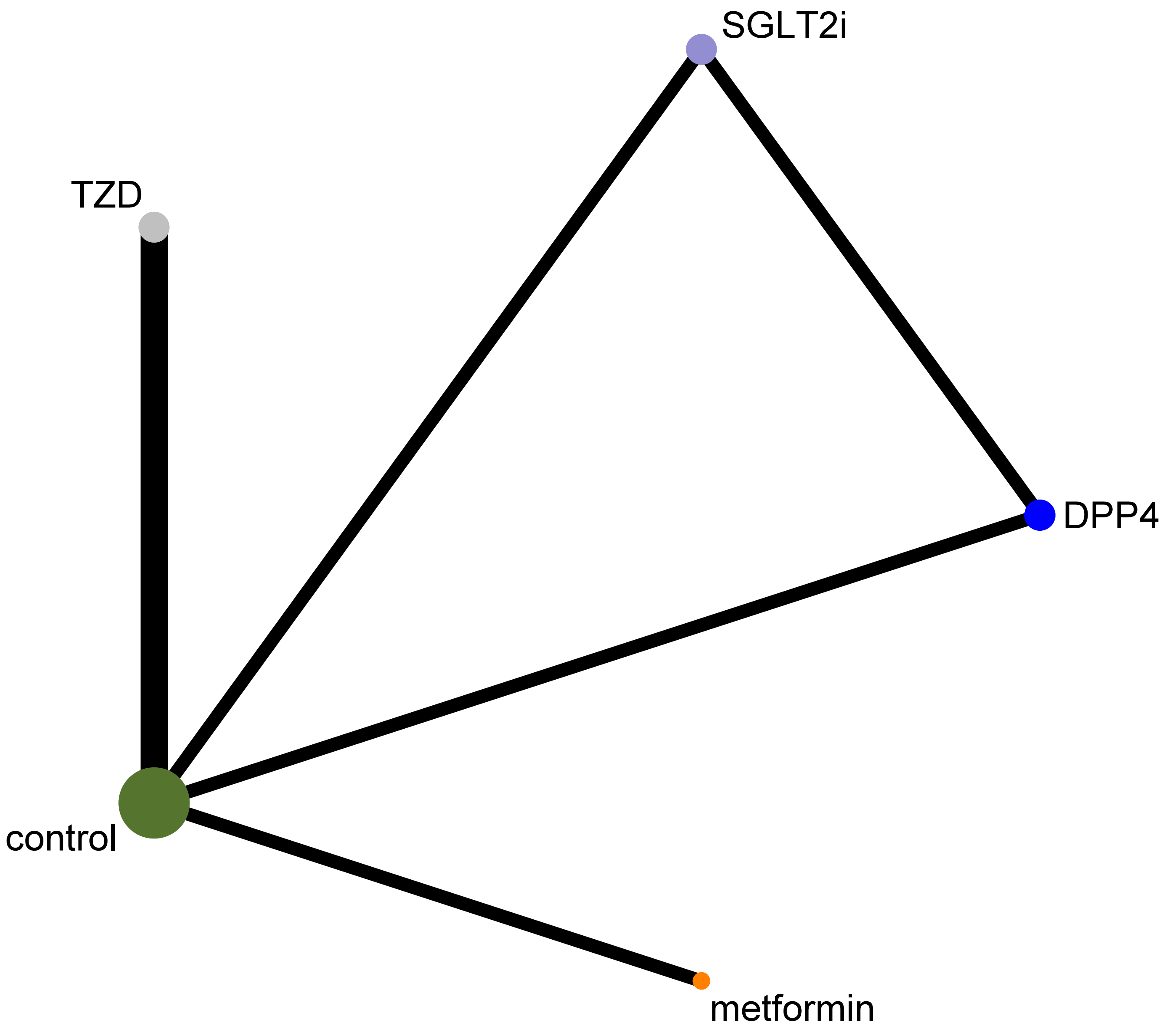

Fig.s7 Network plot for Adionectin

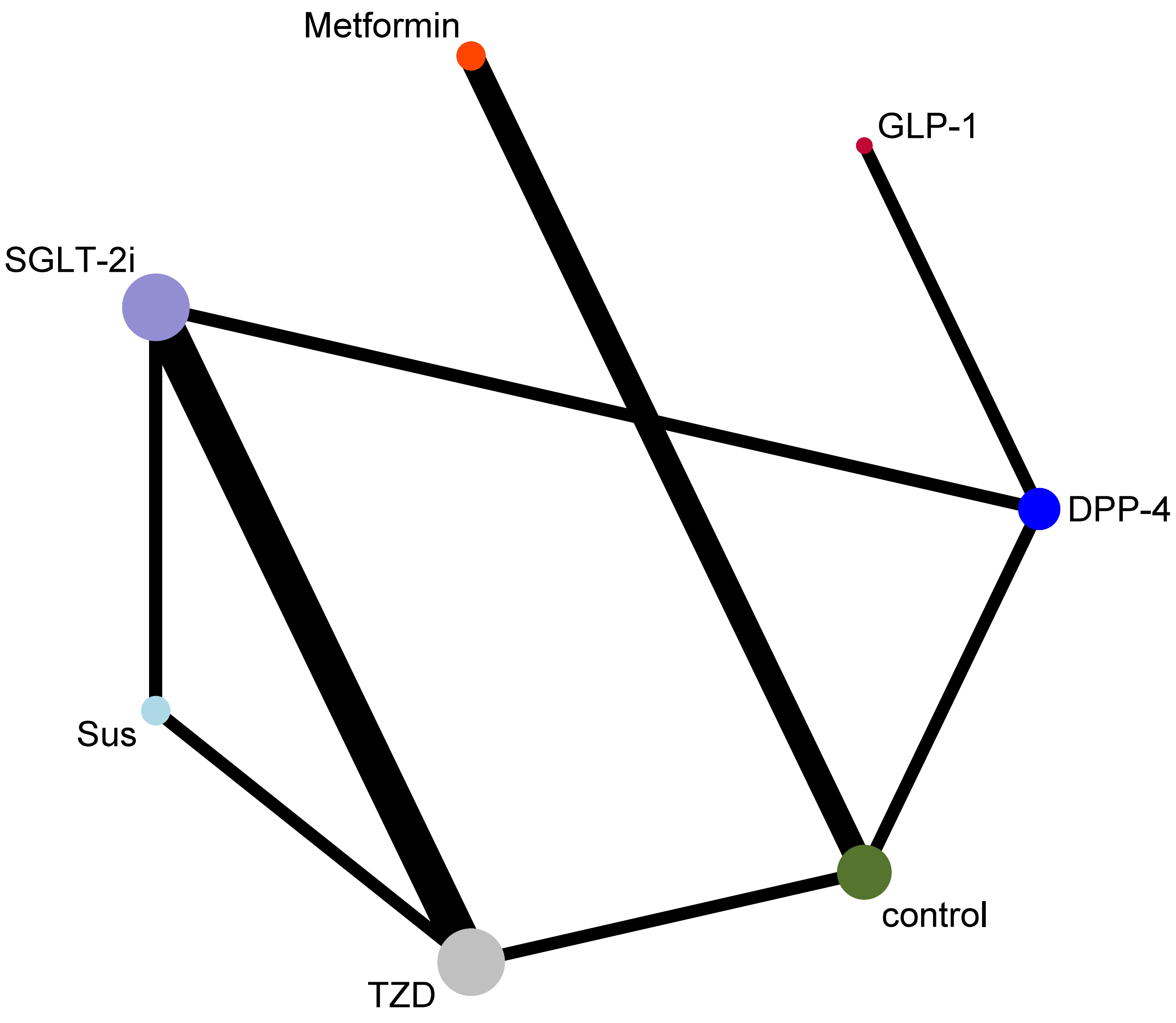

Fig.s8 Network plot for FBS

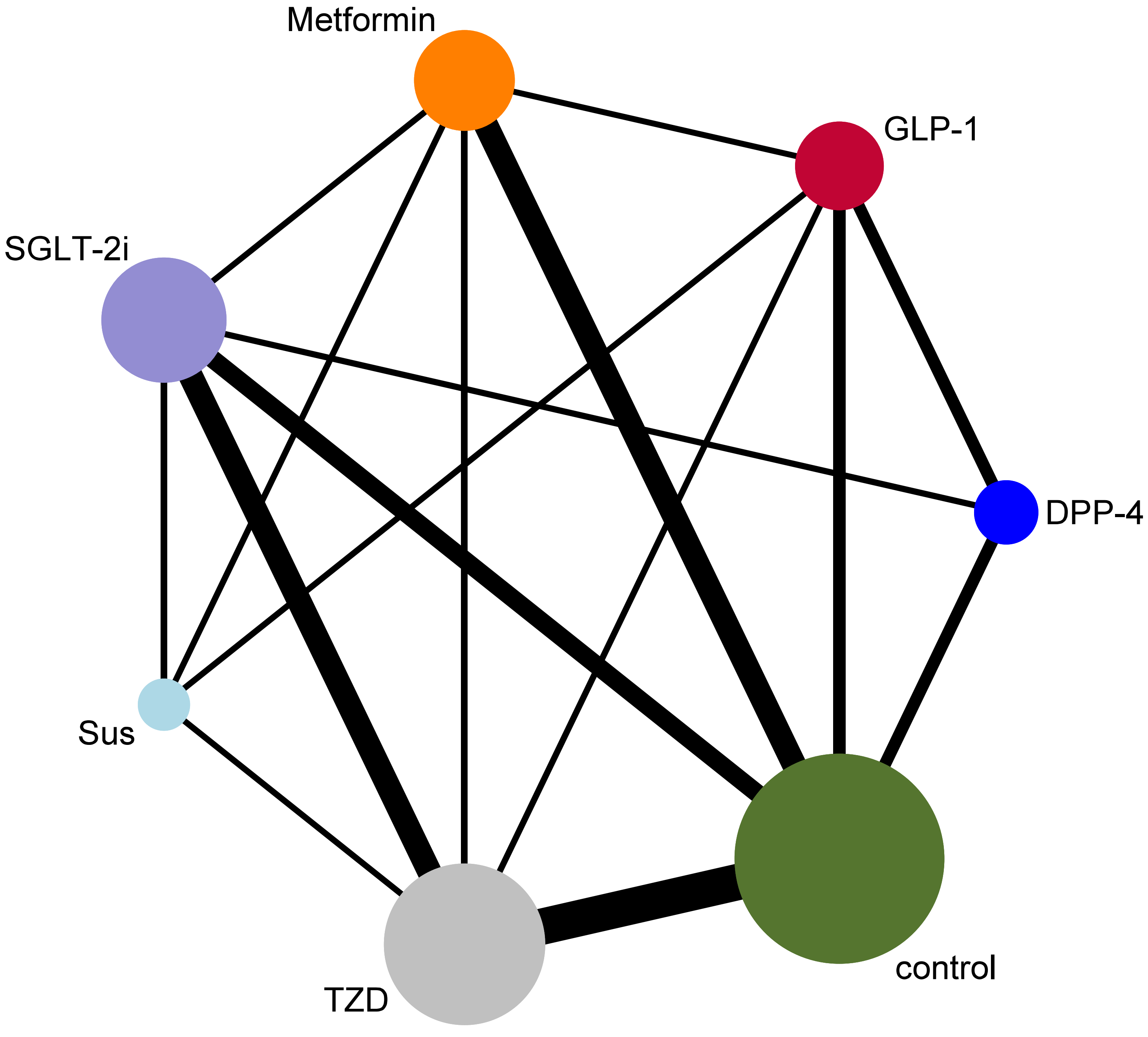

Fig.s9 Network plot for TG

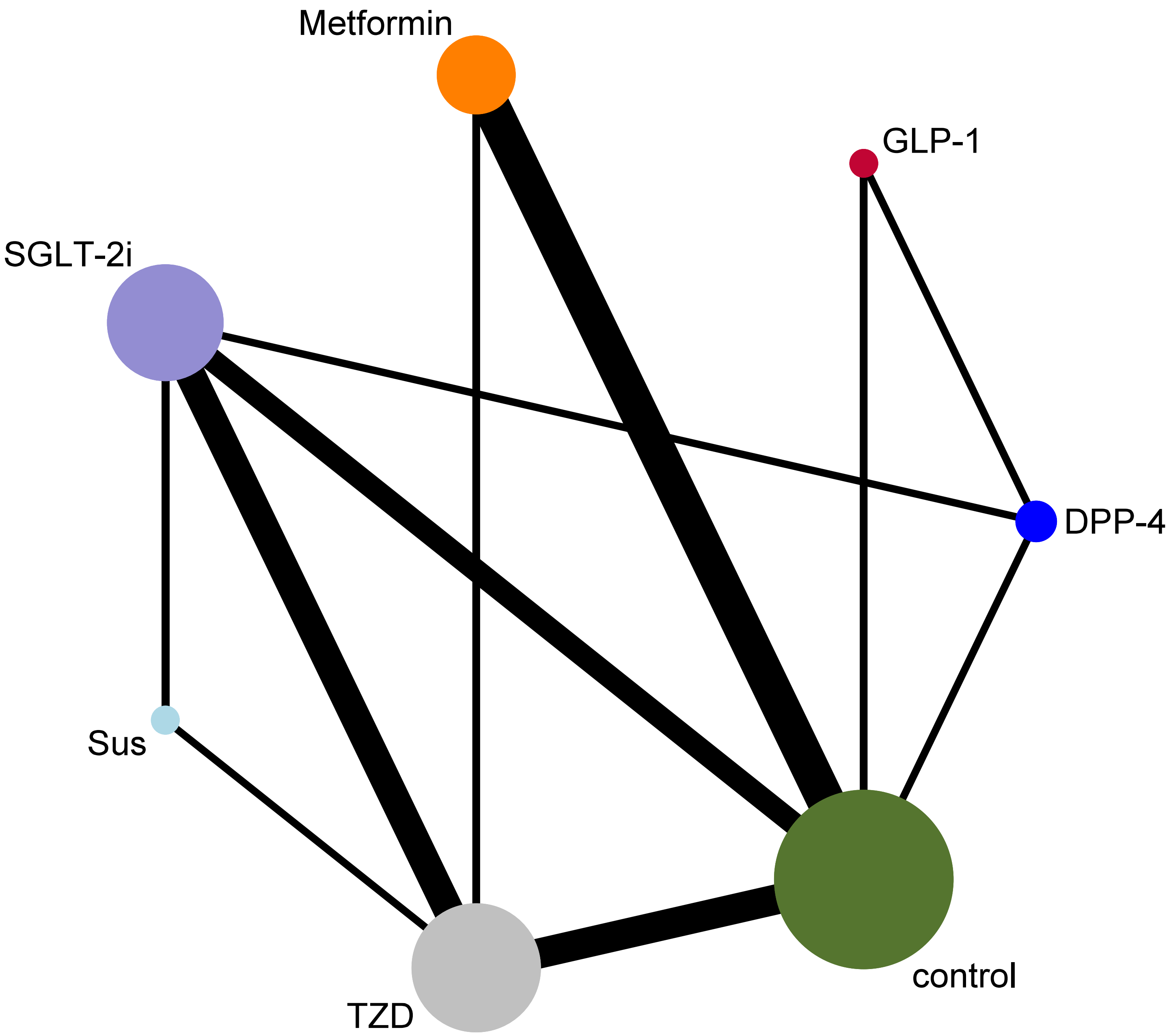

Fig.s10 Network plot for TC

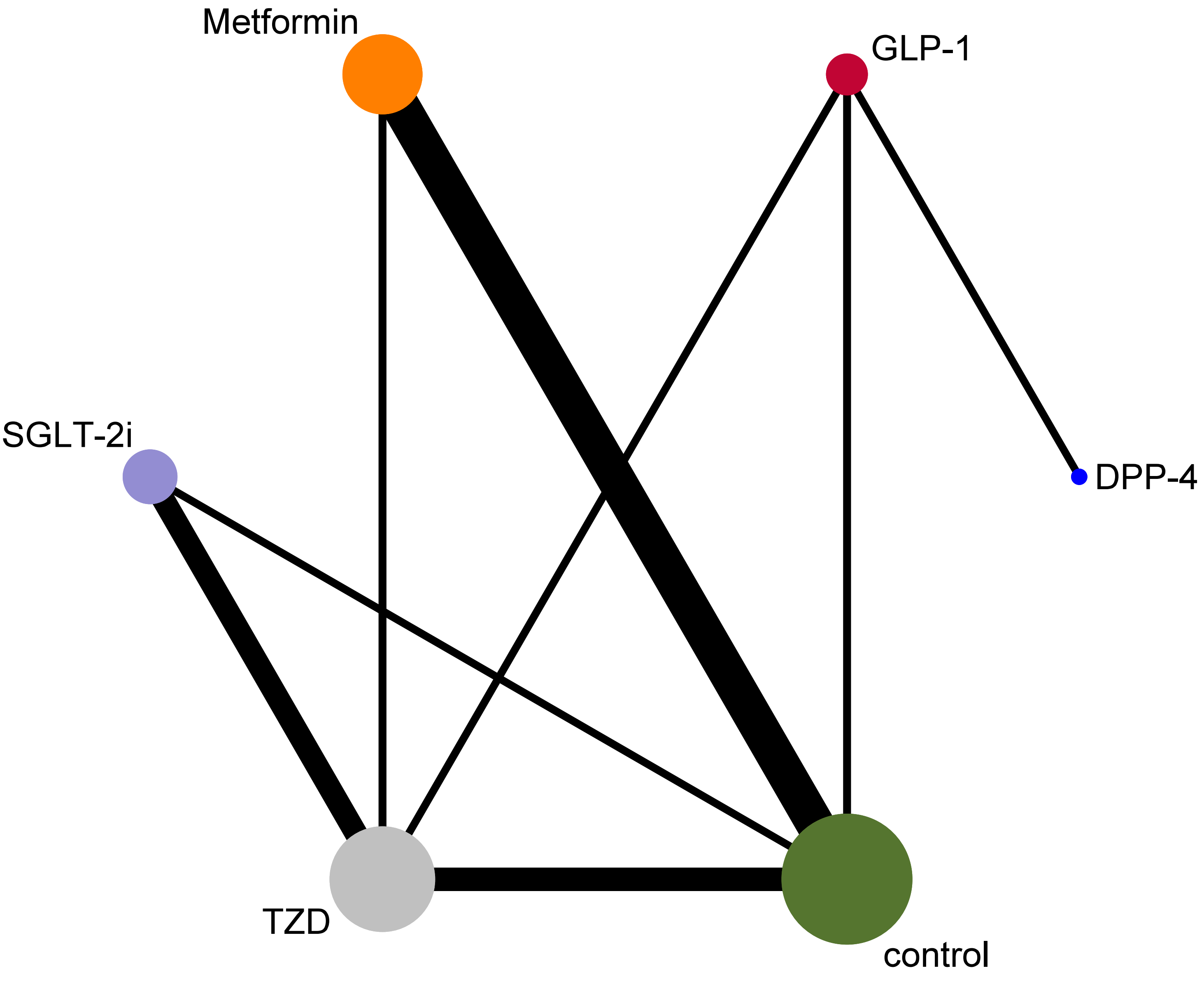

Fig.s11 Network plot for HDL

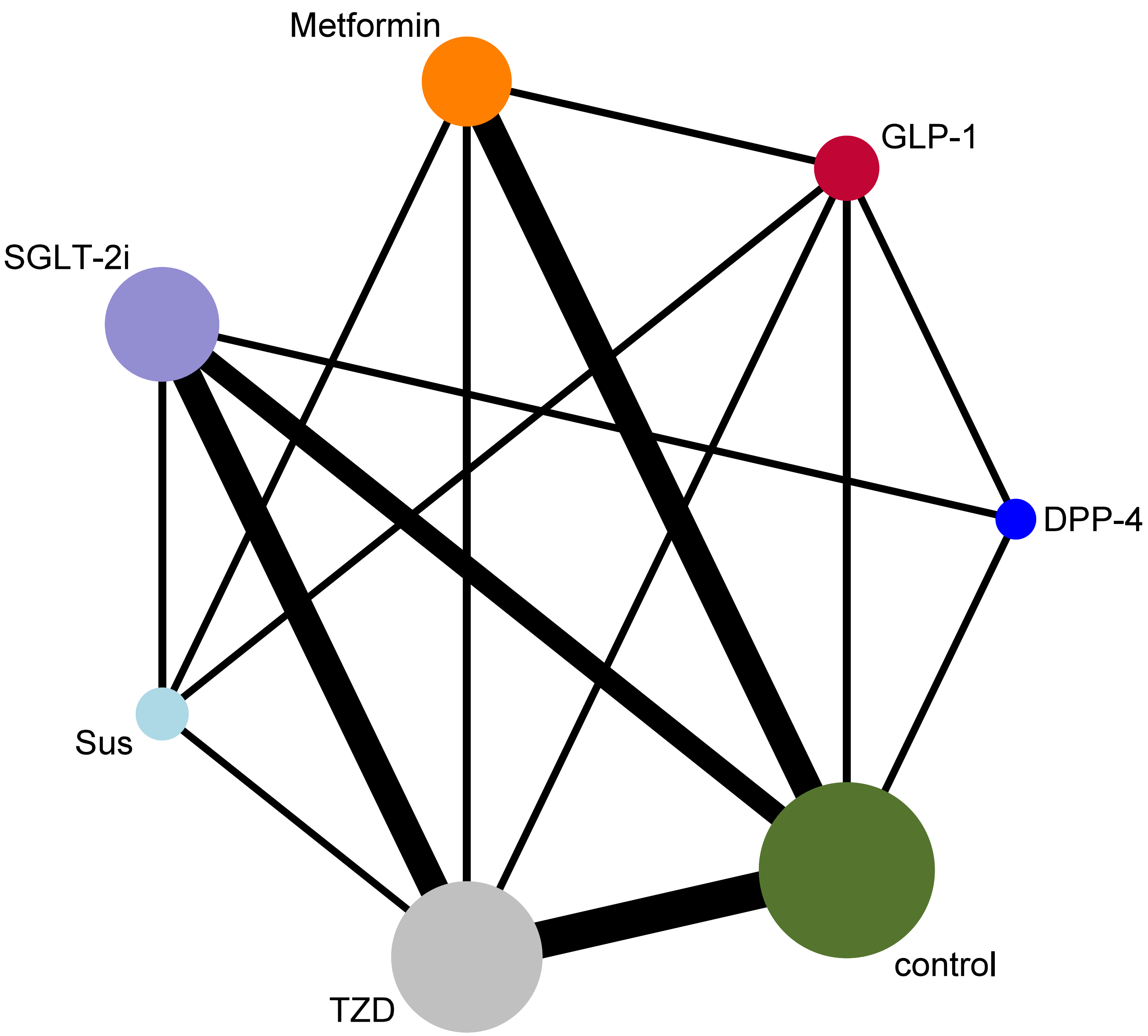

Fig.s12 Network plot for LDL

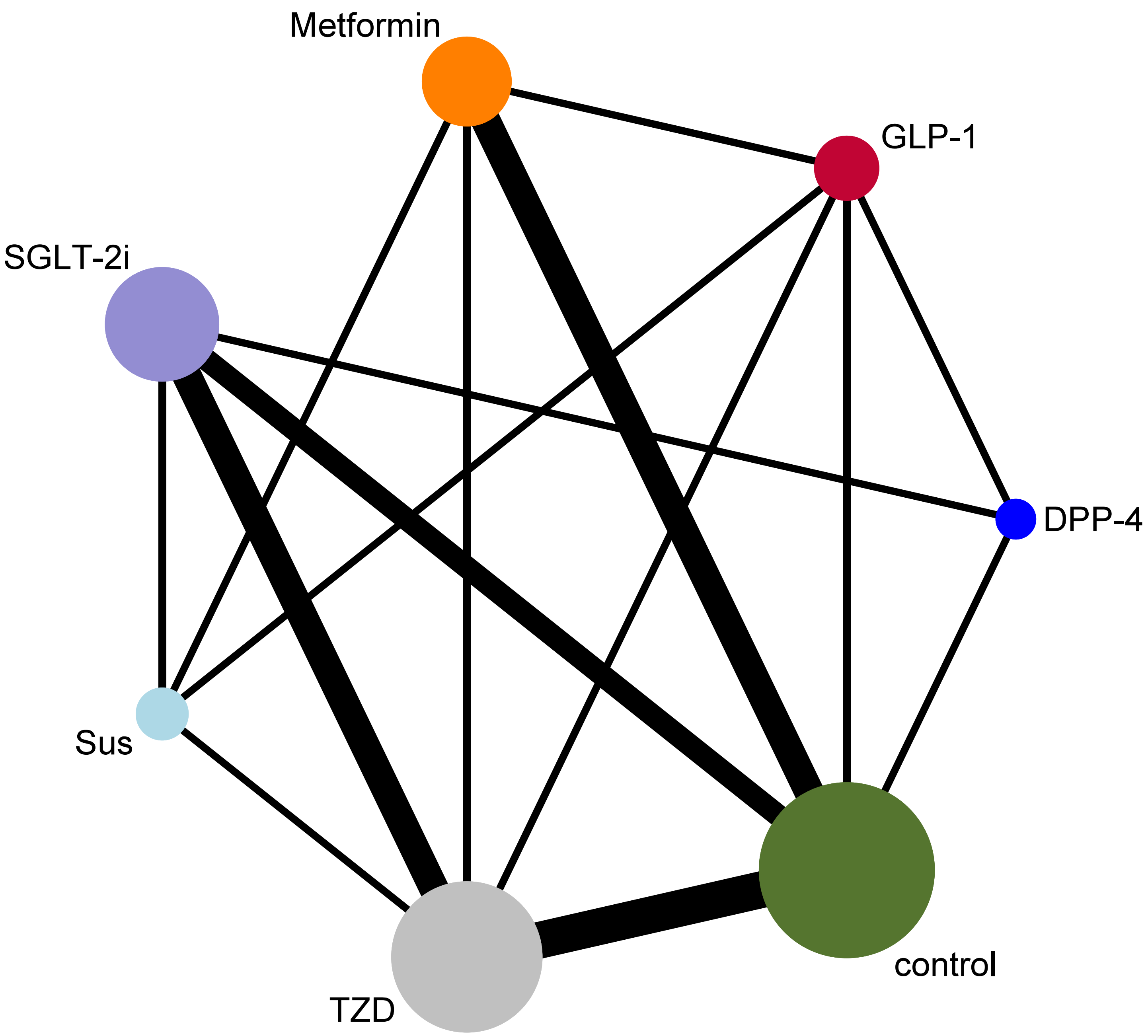

Fig.s13 Network plot for SBP

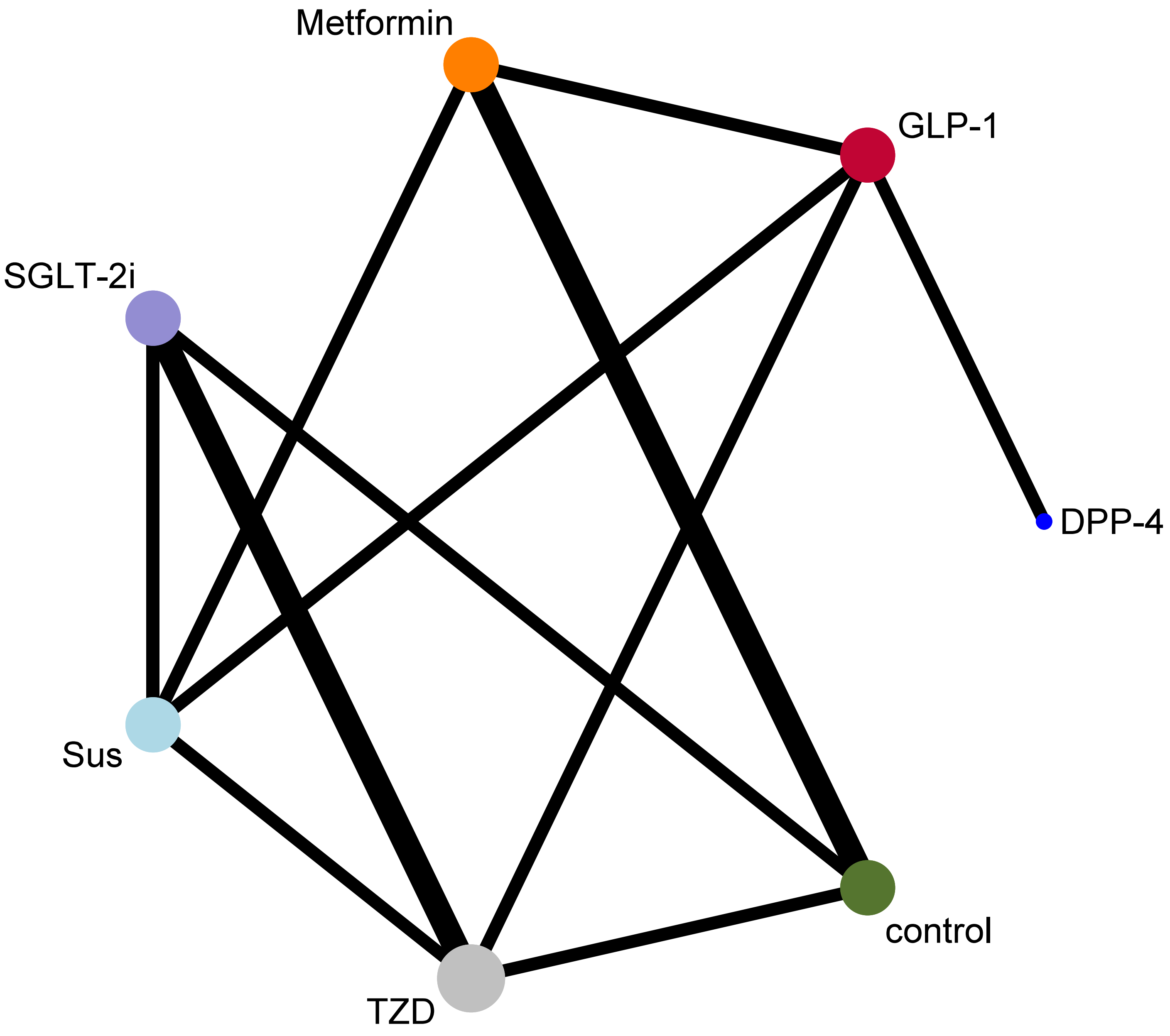

Fig.s14 Network plot for DBP

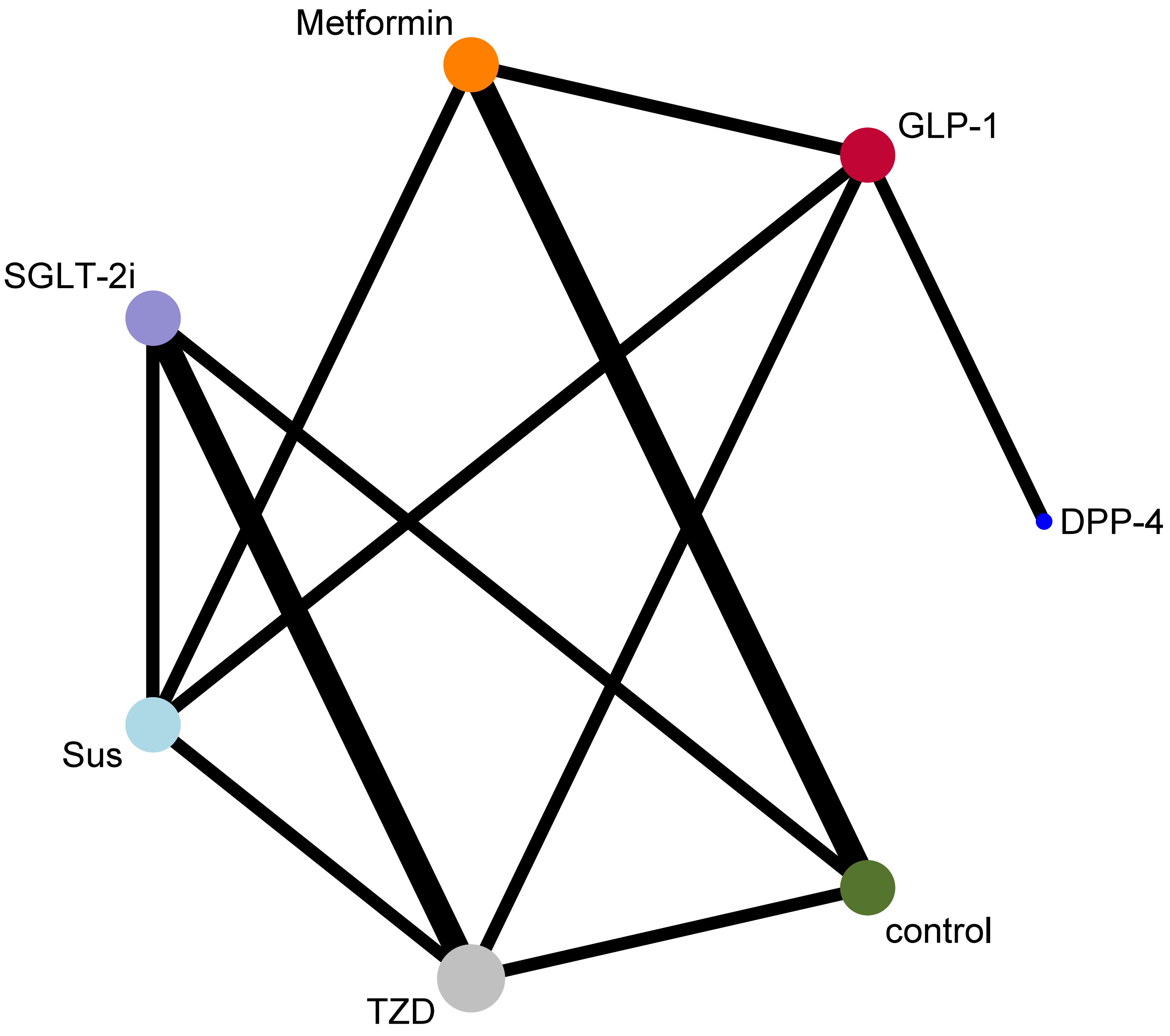

Fig.s15 Network plot for AST

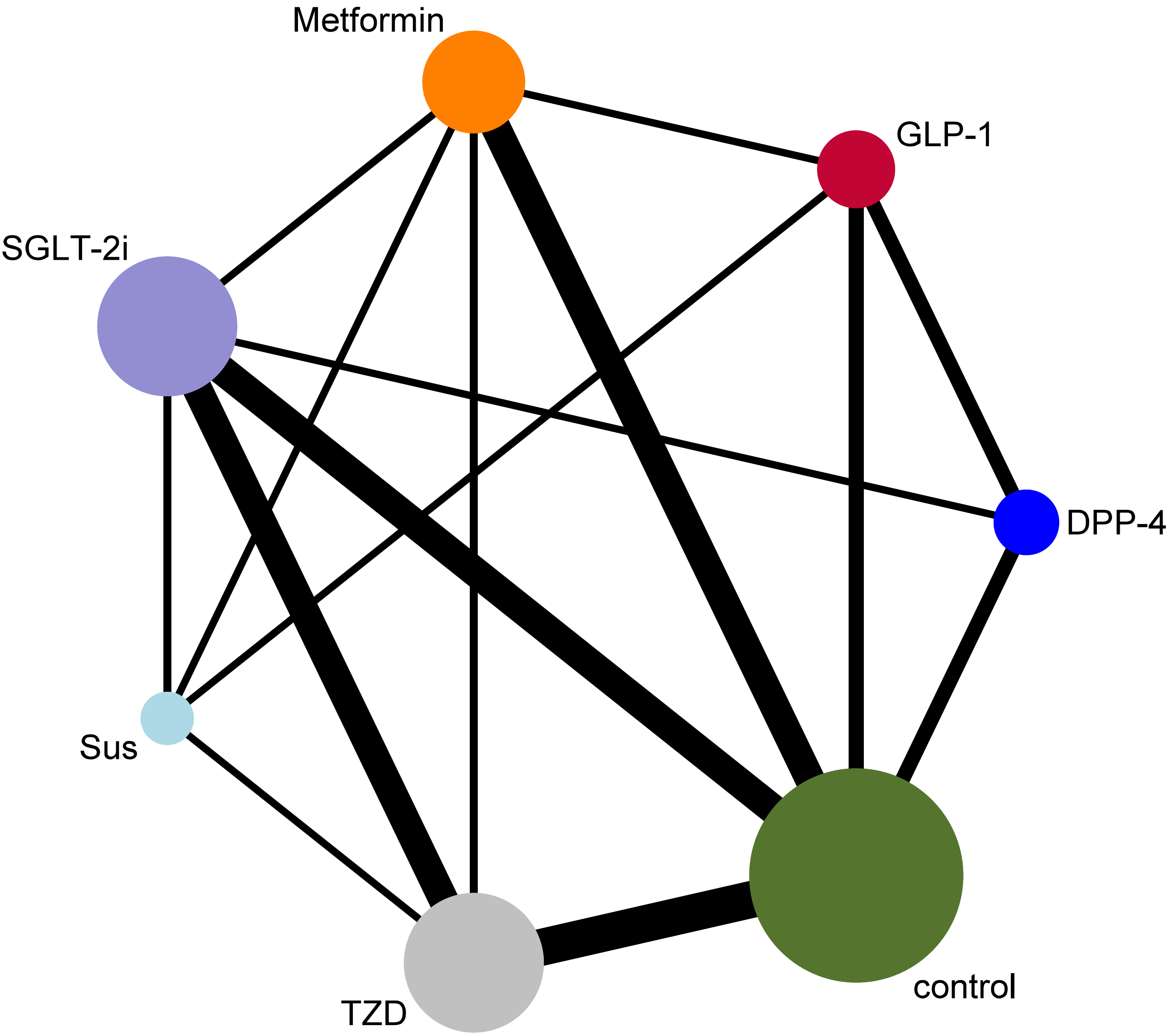

Fig.s16 Network plot for ALT

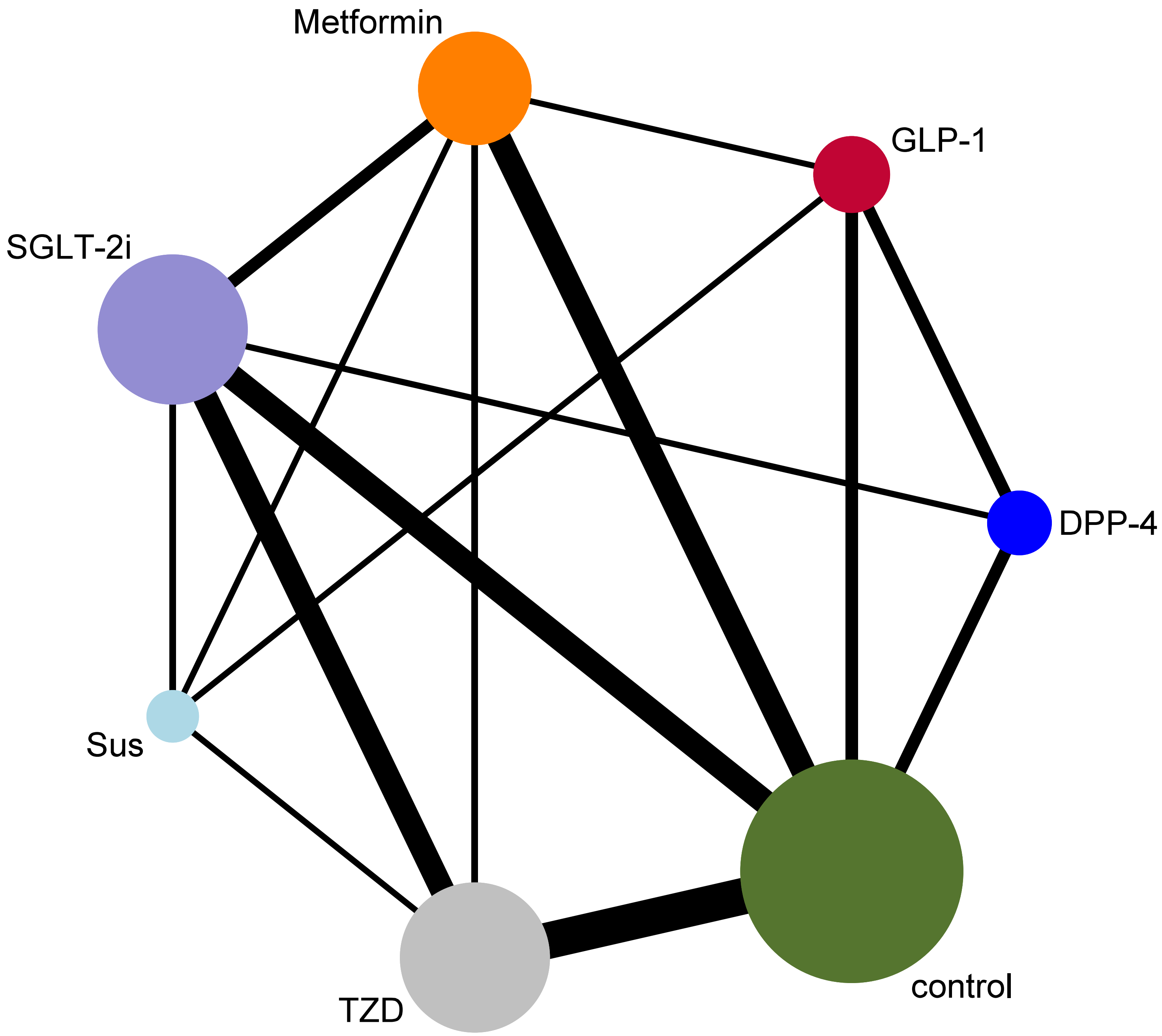

Appendix 5 Model fit statistics for all outcomes

Outcome	Model	DIC	Dbar	pD	ratio	I^2	Used in base case analyses
HOMA-IR	FE	52.73121	31.6716	21.05961	0.9897	2.000%	NO
	RAN	54.04235	30.27051	23.77185	0.946	0.000%	YES
VAT	FE	33.96985	19.9436	14.02625	1.108	15.000%	NO
	RAN	34.21679	17.83497	16.38182	0.9908	4.000%	YES
SAT	FE	15.96671	7.98354	7.983172	0.9979	12.000%	YES
	RAN	15.93458	7.967794	7.966786	0.996	12.000%	NO
BMI	FE	72.01006	49.01922	22.99084	1.362	29.000%	NO
	RAN	64.04594	34.14233	29.90361	0.9484	0.000%	YES
Weight	FE	57.0633	34.01555	23.04775	0.9193	0.000%	YES
	RAN	58.50517	33.19511	25.31006	0.8972	0.000%	NO
LEPTIN	FE	18.88895	9.904476	8.984473	0.9904	9.000%	NO
	RAN	19.18426	9.805453	9.378808	0.9805	8.000%	YES
Adiponectin	FE	39.13536	25.13102	14.00434	1.478	36.000%	NO
	RAN	34.30251	17.46657	16.83595	1.027	8.000%	YES
FBS	FE	166.9528	135.8877	31.06513	2.954	67.000%	NO
	RAN	86.67001	47.8542	38.8158	1.04	6.000%	YES
HbA1C	FE	247.296	225.2899	22.00612	6.258	84.000%	NO
	RAN	70.73826	35.97271	34.76556	0.9992	3.000%	YES
TG	FE	66.65405	43.67948	22.97457	1.181	18.000%	NO
	RAN	67.03778	39.29897	27.73882	1.062	8.000%	YES
TC	FE	42.74546	25.65956	17.0859	0.9869	3.000%	YES
	RAN	43.94261	24.55648	19.38612	0.9445	0.000%	NO
HDL	FE	103.712	78.61189	25.10011	1.872	48.000%	NO
	RAN	78.66469	42.69077	35.97392	1.016	4.000%	YES
LDL	FE	105.3713	80.12119	25.25012	1.908	49.000%	NO
	RAN	82.38497	44.89084	37.49412	1.069	9.000%	YES
AST	FE	87.57976	62.17176	25.408	1.48	34.000%	NO
	RAN	74.98211	41.78366	33.19845	0.9948	2.000%	YES
ALT	FE	149.0912	121.6897	27.40147	2.645	63.000%	NO
	RAN	84.07908	44.58658	39.4925	0.9693	0.000%	YES
SBP	FE	39.39381	23.38299	16.01082	1.063	10.000%	NO
	RAN	39.99386	21.51153	18.48233	0.9778	2.000%	YES
RBP	FE	39.85949	23.79597	16.06352	1.082	12.000%	NO
	RAN	40.35654	21.57362	18.78292	0.9806	3.000%	YES

Appendix 6 Direct, indirect and network treatment estimates

HOMA-IR					
Intervention	Comparator	Direct estimate	Indirect estimate		Network estimate
DPP4	GLP1	-0.28 (-2.5, 2.0)	-3.2 (-5.4, -0.97)	0.07139	-1.7 (-3.4, -0.080)
DPP4	SGLT2i	-2.3 (-5.2, 0.65)	0.22 (-1.8, 2.1)	0.16513	-0.55 (-2.3, 1.1)
GLP1	TZD	0.70 (-1.2, 2.6)	0.32 (-1.5, 1.7)	0.72031	0.47 (-0.73, 1.5)
SGLT2i	TZD	-0.87 (-1.9, 0.14)	0.084 (-1.6, 2.3)	0.32211	-0.75 (-1.4, 0.086)
DPP4	control	-0.76 (-3.7, 2.1)	0.018 (-1.9, 2.)	0.64307	-0.19 (-1.8, 1.4)
GLP1	control	1.8 (0.41, 3.2)	1.0 (-0.81, 2.7)	0.44376	1.6 (0.46, 2.5)
SGLT2i	control	0.22 (-0.71, 1.3)	0.81 (-0.58, 2.4)	0.45312	0.34 (-0.23, 1.1)
TZD	control	0.93 (-0.53, 2.3)	1.2 (-0.16, 2.5)	0.7847	1.1 (0.31, 1.9)
control	metformin	-0.13 (-1.4, 1.2)	1.6 (-0.41, 3.5)	0.14773	0.38 (-0.72, 1.5)
SGLT2i	metformin	1.8 (-0.080, 3.7)	0.10 (-1.3, 1.7)	0.16226	0.73 (-0.40, 2.0)

VAT					
Intervention	Comparator	Direct estimate	Indirect estimate	p value	Network estimate
TZD	control	-0.053 (-0.71, 0.60)	-0.018 (-0.69, 0.57)	0.9209	-0.015 (-0.32, 0.23)

BMI					
Intervention	Comparator	Direct estimate	Indirect estimate	p value	Network estimate
DPP4	GLP1	-0.29 (-3.0, 2.5)	-1.4 (-5.7, 2.8)	0.64847	-0.59 (-2.9, 1.6)
DPP4	SGLT2i	-1.3 (-5.6, 3.1)	0.21 (-2.9, 3.6)	0.57127	-0.28 (-2.8, 2.3)
GLP1	TZD	3.6 (1.1, 6.1)	0.46 (-1.2, 2.3)	0.04853	1.6 (-0.023, 3.3)
GLP1	metformin	-0.66 (-2.7, 1.3)	1.4 (-0.87, 3.4)	0.15348	0.12 (-1.3, 1.8)
DPP4	control	1.1 (-6.4, 8.9)	0.64 (-1.9, 3.2)	0.90678	0.70 (-1.7, 3.2)
GLP1	control	1.3 (-1.8, 4.4)	1.3 (-0.55, 3.4)	0.98956	1.3 (-0.19, 2.9)
SGLT2i	control	0.91 (-0.77, 2.6)	1.2 (-1.6, 4.1)	0.86902	0.98 (-0.40, 2.4)
TZD	control	-0.24 (-1.1, 0.75)	-2.6 (-5.3, 0.0025)	0.08271	-0.28 (-1.4, 0.86)
control	metformin	-1.0 (-2.2, 0.19)	-2.2 (-3.7, -0.53)	0.23262	-1.2 (-2.4, 0.14)
SGLT2i	metformin	-0.39 (-3.4, 2.6)	-0.062 (-2.1, 2.1)	0.8522	-0.17 (-1.8, 1.5)
TZD	metformin	0.80 (-0.93, 2.5)	-2.8 (-4.0, -1.4)	0.00591	-1.4 (-2.9, 0.11)

Weight					
Intervention	Comparator	Direct estimate	Indirect estimate	p value	Network estimate
DPP4	GLP1	-3.6 (-11., 3.9)	-3.8 (-18., 10.)	0.96857	-3.7 (-10., 2.7)
DPP4	SGLT2i	-3.3 (-18., 12.)	-1.6 (-9.2, 6.4)	0.84359	-2.1 (-8.7, 4.7)
GLP1	SUs	3.8 (0.55, 7.2)	6.9 (1.5, 12.)	0.28787	4.3 (1.9, 7.4)
GLP1	TZD	9.9 (4.4, 15.)	3.9 (0.52, 6.9)	0.06013	5.1 (2.4, 8.4)
GLP1	metformin	-4.3 (-7.6, -0.94)	5.9 (-4.3, 16.)	0.06169	-3.8 (-6.1, 0.25)
SGLT2i	SUs	2.8 (-1.6, 7.2)	2.1 (-3.8, 9.1)	0.81428	2.7 (0.078, 5.6)
SGLT2i	TZD	2.8 (-0.82, 5.8)	6.2 (0.96, 12.)	0.23798	3.5 (1.3, 5.9)
SUs	TZD	0.38 (-4.2, 4.9)	1.8 (-4.8, 7.2)	0.63809	0.80 (-1.9, 3.5)
DPP4	control	5.2 (-28., 36.)	-0.96 (-8.4, 6.2)	0.71945	-0.95 (-7.7, 5.8)
GLP1	control	2.2 (-4.2, 8.5)	3. (-0.68, 7.3)	0.82101	2.7 (-0.24, 6.1)
SGLT2i	control	2.1 (-1.5, 5.8)	0.13 (-4.5, 4.2)	0.4471	1.1 (-1.3, 3.7)
TZD	control	-2.3 (-5.2, 0.66)	-2.6 (-7.3, 1.7)	0.89831	-2.4 (-4.7, -0.19)
control	metformin	1.2 (-8.5, 11.)	-7.3 (-12., -3.7)	0.10868	-6.4 (-9.8, -2.3)
SUs	metformin	-8.1 (-11., -4.8)	-0.95 (-11., 9.3)	0.16962	-8.0 (-11., -4.4)

LEPTIN					
Intervention	Comparator	Direct estimate	Indirect estimate	p value	Network estimate
DPP4	SGLT2i	-6.3 (-24., 11.)	3.6 (-17., 24.)	0.44967	-1.8 (-15., 11.)
DPP4	control	8.7 (-8.2, 26.)	-1.0 (-22., 20.)	0.45479	4.7 (-8.2, 18.)
SGLT2i	control	5.1 (-6.9, 17.)	15. (-9.4, 39.)	0.45543	6.4 (-3.2, 18.)

FBS					
Intervention	Comparator	Direct estimate	Indirect estimate	p value	Network estimate
DPP4	GLP1	-0.30 (-1.9, 1.3)	-0.10 (-1.4, 1.2)	0.85264	-0.18 (-1.2, 0.80)
DPP4	SGLT2i	0.088 (-0.99, 1.2)	0.36 (-1.3, 2.1)	0.78897	0.15 (-0.68, 1.0)
GLP1	SUs	0.61 (-0.43, 1.7)	1.6 (0.29, 2.9)	0.22515	0.95 (0.22, 1.8)
GLP1	TZD	1.1 (-0.66, 2.9)	0.046 (-0.72, 0.83)	0.27588	0.20 (-0.49, 0.93)
GLP1	metformin	0.28 (-0.76, 1.3)	0.93 (-0.070, 2.0)	0.34889	0.52 (-0.12, 1.3)
SGLT2i	SUs	0.78 (-0.29, 1.9)	0.32 (-0.80, 1.5)	0.5149	0.61 (-0.083, 1.3)
SGLT2i	TZD	-0.25 (-0.95, 0.41)	0.049 (-0.84, 0.97)	0.56726	-0.14 (-0.64, 0.35)
SUs	TZD	-1.0 (-2.1, 0.032)	-0.43 (-1.5, 0.64)	0.38581	-0.75 (-1.4, -0.076)
DPP4	control	3.2 (-1.5, 7.9)	0.39 (-0.56, 1.4)	0.25575	0.48 (-0.43, 1.4)
GLP1	control	0.90 (-0.38, 2.2)	0.58 (-0.20, 1.5)	0.67466	0.66 (0.018, 1.4)
SGLT2i	control	0.25 (-0.51, 1.1)	0.39 (-0.33, 1.1)	0.79212	0.33 (-0.16, 0.83)
TZD	control	0.57 (0.081, 1.1)	0.51 (-0.31, 1.3)	0.89155	0.47 (0.044, 0.91)
control	metformin	-0.080 (-0.51, 0.48)	-0.19 (-1.1, 0.63)	0.81159	-0.15 (-0.55, 0.31)
SGLT2i	metformin	0.40 (-0.81, 1.6)	0.13 (-0.49, 0.83)	0.68364	0.18 (-0.35, 0.77)
SUs	metformin	-0.33 (-1.4, 0.72)	-0.66 (-1.6, 0.48)	0.62555	-0.43 (-1.1, 0.30)
TZD	metformin	-0.39 (-1.2, 0.46)	0.59 (0.091, 1.2)	0.04586	0.32 (-0.16, 0.88)

TC					
Intervention	Comparator	Direct estimate	Indirect estimate	p value	Network estimate
GLP1	TZD	-0.29 (-1.1, 0.53)	0.45 (-0.44, 1.3)	0.22593	0.048 (-0.55, 0.64)
GLP1	control	0.60 (-0.18, 1.4)	-0.15 (-1.0, 0.72)	0.20707	0.26 (-0.33, 0.84)
SGLT2i	control	0.33 (-0.26, 0.93)	0.35 (-0.31, 1.1)	0.96276	0.35 (-0.037, 0.75)
TZD	control	0.20 (-0.17, 0.59)	0.90 (-0.24, 2.0)	0.2477	0.21 (-0.10, 0.54)
TZD	metformin	0.035 (-0.59, 0.65)	0.15 (-0.30, 0.59)	0.76213	0.15 (-0.23, 0.50)

TG					
Intervention	Comparator	Direct estimate	Indirect estimate	p value	Network estimate
DPP4	GLP1	-0.088 (-1.0, 0.84)	-0.0036 (-0.82, 0.87)	0.8908	-0.047 (-0.66, 0.58)
DPP4	SGLT2i	0.43 (-0.36, 1.2)	0.74 (-0.11, 1.6)	0.59466	0.57 (0.011, 1.1)
SGLT2i	TZD	-0.11 (-0.41, 0.24)	-0.11 (-0.64, 0.48)	0.9952	-0.12 (-0.33, 0.14)
DPP4	control	1.0 (-0.18, 2.3)	0.44 (-0.20, 1.1)	0.39561	0.58 (0.019, 1.1)
GLP1	control	0.60 (0.094, 1.1)	0.69 (-0.45, 1.9)	0.88799	0.62 (0.18, 1.1)
SGLT2i	control	0.0070 (-0.34, 0.38)	0.026 (-0.40, 0.51)	0.93028	-0.0015 (-0.24, 0.27)
TZD	control	0.093 (-0.29, 0.48)	0.17 (-0.43, 0.81)	0.80723	0.12 (-0.13, 0.35)
TZD	metformin	0.30 (-0.28, 0.88)	0.20 (-0.26, 0.74)	0.7454	0.23 (-0.055, 0.56)

HDL					
Intervention	Comparator	Direct estimate	Indirect estimate	p value	Network estimate
DPP4	GLP1	0.00015 (-0.26, 0.26)	0.11 (-0.18, 0.44)	0.56204	0.045 (-0.14, 0.25)
DPP4	SGLT2i	0.22 (-0.081, 0.52)	0.029 (-0.26, 0.29)	0.33644	0.12 (-0.086, 0.31)
GLP1	SUs	0.090 (-0.11, 0.29)	-0.22 (-0.49, 0.035)	0.05569	-0.013 (-0.21, 0.14)
GLP1	TZD	-0.20 (-0.47, 0.070)	0.27 (0.11, 0.39)	0.00619	0.13 (-0.054, 0.28)
GLP1	metformin	0.16 (-0.034, 0.35)	-0.16 (-0.40, 0.061)	0.03203	0.038 (-0.15, 0.18)
SGLT2i	SUs	-0.060 (-0.26, 0.14)	-0.063 (-0.27, 0.18)	0.97096	-0.085 (-0.24, 0.074)
SGLT2i	TZD	-0.060 (-0.26, 0.14)	-0.063 (-0.27, 0.18)	0.19886	-0.085 (-0.24, 0.074)
SUs	TZD	0.26 (0.061, 0.46)	0.081 (-0.16, 0.27)	0.1579	0.15 (-0.013, 0.29)
DPP4	control	-0.011 (-0.46, 0.44)	0.12 (-0.12, 0.34)	0.60325	0.10 (-0.11, 0.29)
GLP1	control	-0.20 (-0.62, 0.23)	0.094 (-0.094, 0.24)	0.22022	0.056 (-0.13, 0.20)
SGLT2i	control	-0.025 (-0.19, 0.14)	-0.0093 (-0.20, 0.16)	0.88933	-0.014 (-0.13, 0.093)
TZD	control	-0.076 (-0.20, 0.054)	-0.086 (-0.30, 0.13)	0.92941	-0.079 (-0.17, 0.018)
control	metformin	-0.051 (-0.18, 0.081)	0.11 (-0.14, 0.40)	0.2313	-0.020 (-0.13, 0.092)
SUs	metformin	0.070 (-0.13, 0.26)	0.057 (-0.16, 0.27)	0.92242	0.052 (-0.12, 0.20)
TZD	metformin	-0.12 (-0.41, 0.18)	-0.092 (-0.25, 0.077)	0.85851	-0.099 (-0.23, 0.034)

LDL					
Intervention	Comparator	Direct estimate	Indirect estimate	p value	Network estimate
DPP4	GLP1	-0.20 (-1.0, 0.60)	0.093 (-0.68, 0.87)	0.58528	-0.047 (-0.58, 0.49)
DPP4	SGLT2i	0.28 (-0.65, 1.2)	-0.29 (-0.96, 0.37)	0.30678	-0.11 (-0.64, 0.44)
GLP1	SUs	0.17 (-0.59, 0.94)	0.24 (-0.64, 1.1)	0.89713	0.19 (-0.30, 0.69)
GLP1	TZD	0.0032 (-0.96, 0.96)	-0.11 (-0.62, 0.41)	0.83037	-0.093 (-0.52, 0.35)
GLP1	metformin	-0.021 (-0.78, 0.74)	0.017 (-0.68, 0.71)	0.93791	-0.0056 (-0.45, 0.44)
SGLT2i	SUs	0.24 (-0.52, 1.0)	0.23 (-0.60, 1.0)	0.9884	0.26 (-0.22, 0.71)
SGLT2i	TZD	0.016 (-0.41, 0.44)	-0.19 (-0.88, 0.48)	0.58701	-0.028 (-0.34, 0.27)
SUs	TZD	-0.33 (-1.1, 0.44)	-0.25 (-1.0, 0.55)	0.86892	-0.28 (-0.73, 0.17)
DPP4	control	-0.12 (-0.95, 0.72)	0.076 (-0.60, 0.79)	0.71147	-0.0051 (-0.51, 0.52)
GLP1	control	-0.10 (-0.88, 0.68)	0.11 (-0.39, 0.64)	0.62855	0.041 (-0.36, 0.47)
SGLT2i	control	0.14 (-0.32, 0.60)	0.053 (-0.47, 0.58)	0.77985	0.11 (-0.21, 0.42)
TZD	control	0.24 (-0.093, 0.60)	-0.015 (-0.55, 0.49)	0.36732	0.13 (-0.14, 0.42)
control	metformin	-0.13 (-0.50, 0.18)	0.026 (-0.60, 0.64)	0.6242	-0.047 (-0.39, 0.27)
SUs	metformin	-0.19 (-0.95, 0.57)	-0.22 (-1.1, 0.59)	0.9486	-0.20 (-0.68, 0.27)
TZD	metformin	0.23 (-0.28, 0.73)	0.12 (-0.094, 0.30)	0.66501	0.087 (-0.31, 0.47)

SBP					
Intervention	Comparator	Direct estimate	Indirect estimate	p value	Network estimate
GLP1	SUs	6.2 (-3.2, 16.)	8.8 (-6.9, 24.)	0.72733	6.5 (0.49, 13.)
GLP1	TZD	3.2 (-9.0, 15.)	0.65 (-8., 11.)	0.74069	1.0 (-4.7, 9.0)
GLP1	metformin	4.3 (-5.0, 14.)	4.6 (-13., 22.)	0.98324	4.3 (-2.3, 11.)
SGLT2i	SUs	5.8 (-3.0, 15.)	6.4 (-6.3, 20.)	0.91789	6.0 (0.21, 13.)
SGLT2i	TZD	1.4 (-4.6, 10.)	-1.5 (-16., 12.)	0.65127	0.53 (-3.6, 7.1)
SUs	TZD	-6.5 (-15., 2.2)	-2.3 (-14., 8.7)	0.46788	-5.6 (-10., 1.2)
SGLT2i	control	-1.0 (-11., 9.4)	2.4 (-5.6, 13.)	0.56456	0.98 (-4.9, 7.8)
TZD	control	0.88 (-7.6, 9.4)	-0.56 (-11., 7.8)	0.78831	0.27 (-5.9, 5.7)
control	metformin	1.8 (-7.1, 11.)	3.7 (-8.2, 15.)	0.75883	2.8 (-4.0, 8.9)
SUs	metformin	-1.8 (-11., 7.5)	-4.2 (-18., 10.)	0.72414	-2.2 (-8.7, 3.7)

DBP					
Intervention	Comparator	Direct estimate	Indirect estimate	p value	Network estimate
GLP1	SUs	-0.050 (-7.0, 6.9)	-2.9 (-15., 8.3)	0.60556	-0.34 (-5.1, 3.5)
GLP1	TZD	-2.3 (-11., 6.4)	-0.021 (-6.3, 6.8)	0.62453	-0.41 (-5.3, 3.9)
GLP1	metformin	1.6 (-5.4, 8.6)	-0.26 (-13., 12.)	0.76696	1.5 (-3.1, 5.7)
SGLT2i	SUs	0.30 (-5.8, 6.4)	4.1 (-4.2, 13.)	0.33314	1.0 (-2.4, 5.8)
SGLT2i	TZD	0.72 (-3.6, 6.6)	3.9 (-5.2, 13.)	0.48409	0.98 (-1.8, 5.2)
SUs	TZD	-0.20 (-6.2, 5.8)	-0.37 (-8.3, 6.8)	0.96336	-0.057 (-3.7, 3.9)
SGLT2i	control	0.012 (-5.9, 6.)	-3.4 (-8.2, 2.6)	0.33282	-2. (-5.6, 2.4)
TZD	control	-4.5 (-9.5, 0.81)	-1.9 (-7.7, 3.6)	0.44359	-3.1 (-6.9, 0.42)
control	metformin	5.3 (0.11, 11.)	4.7 (-2.9, 13.)	0.89485	5. (1.4, 9.1)
SUs	metformin	1.6 (-5.5, 8.7)	2.6 (-6.9, 12.)	0.82037	1.9 (-2.0, 6.1)

AST					
Intervention	Comparator	Direct estimate	Indirect estimate	p value	Network estimate
DPP4	GLP1	3.8 (-4.9, 13.)	-1.8 (-14., 11.)	0.46086	2.0 (-5.1, 9.)
DPP4	SGLT2i	-8.1 (-21., 5.5)	2.1 (-6.8, 11.)	0.21128	-0.91 (-8.6, 6.5)
GLP1	SUs	-0.29 (-6.9, 6.3)	1.9 (-8.2, 11.)	0.68363	0.29 (-4.4, 5.1)
GLP1	metformin	-1.4 (-7.9, 5.2)	-0.91 (-9.6, 7.3)	0.92133	-1.5 (-5.9, 2.9)
SGLT2i	SUs	2.5 (-3.2, 8.2)	2.9 (-3.7, 9.4)	0.91665	3.2 (-0.85, 7.5)
SGLT2i	TZD	-0.96 (-4.8, 3.5)	1.4 (-5.4, 8.2)	0.53882	-0.52 (-3.4, 2.9)
SUs	TZD	-5.8 (-11., -0.23)	-1.8 (-8.1, 4.7)	0.27345	-3.8 (-7.7, 0.63)
DPP4	control	7.2 (-13., 27.)	0.52 (-7.6, 8.5)	0.5514	1.4 (-6.1, 8.9)
GLP1	control	2.1 (-6.2, 10.)	-1.6 (-6.9, 4.)	0.44786	-0.67 (-4.9, 4.1)
SGLT2i	control	2.5 (-1.5, 6.8)	2.3 (-2.9, 8.5)	0.95638	2.3 (-0.56, 5.6)
TZD	control	2.5 (-1.1, 6.5)	4.0 (-1.3, 9.2)	0.59026	2.8 (-0.26, 5.9)
control	metformin	0.44 (-2.5, 2.6)	-1.1 (-6.5, 3.2)	0.51064	-0.81 (-4.0, 1.9)

SGLT2i	metformin	-7.3 (-33., 18.)	1.6 (-2.2, 5.6)	0.48961	1.5 (-2.3, 5.3)
SUs	metformin	-1.1 (-7.7, 5.5)	-2.9 (-10., 4.3)	0.65565	-1.8 (-6.0, 2.3)
TZD	metformin	-4.8 (-10., 0.61)	4.7 (1.2, 7.5)	0.00831	2. (-2.0, 5.5)

ALT					
Intervention	Comparator	Direct estimate	Indirect estimate	p value	Network estimate
DPP4	SGLT2i	-8.6 (-45., 29.)	-0.88 (-19., 16.)	0.71477	-2.5 (-19., 13.)
GLP1	SUs	5.9 (-9.5, 21.)	5.3 (-16., 25.)	0.95615	5.7 (-5.6, 17.)
GLP1	metformin	1.0 (-14., 16.)	3.5 (-13., 19.)	0.81337	2.3 (-7.7, 12.)
SGLT2i	SUs	6.9 (-8.6, 22.)	10. (-5.9, 27.)	0.77374	8.4 (-1.2, 18.)
SGLT2i	TZD	-6.8 (-15., 1.5)	-1.7 (-14., 10.)	0.48419	-5.2 (-12., 1.0)
SUs	TZD	-13. (-29., 2.3)	-15. (-32., 0.88)	0.87531	-14. (-24., -4.1)
DPP4	control	-2.9 (-32., 26.)	2.5 (-16., 21.)	0.75765	0.67 (-15., 16.)
GLP1	control	3.4 (-12., 19.)	-1.5 (-14., 11.)	0.61276	0.48 (-9.2, 10.)
SGLT2i	control	3.7 (-5.3, 13.)	2.8 (-6.6, 13.)	0.89073	3.2 (-2.9, 9.7)
TZD	control	8.2 (1.5, 16.)	11. (0.43, 22.)	0.6682	8.4 (2.5, 15.)
control	metformin	4.2 (-2.1, 9.7)	0.33 (-8.2, 10.)	0.47796	1.8 (-4.5, 8.1)
SGLT2i	metformin	9.7 (-4.5, 24.)	3.3 (-5.3, 12.)	0.44491	5. (-2.2, 13.)
SUs	metformin	-5. (-20., 10.)	-1.9 (-17., 14.)	0.75935	-3.4 (-13., 6.5)
TZD	metformin	-4.3 (-15., 6.6)	15. (8.6, 23.)	0.00557	10. (3., 18.)

Appendix 7
The trajectory map, density map and convergent diagnostic diagram

Fig.s1 HOMO-IR

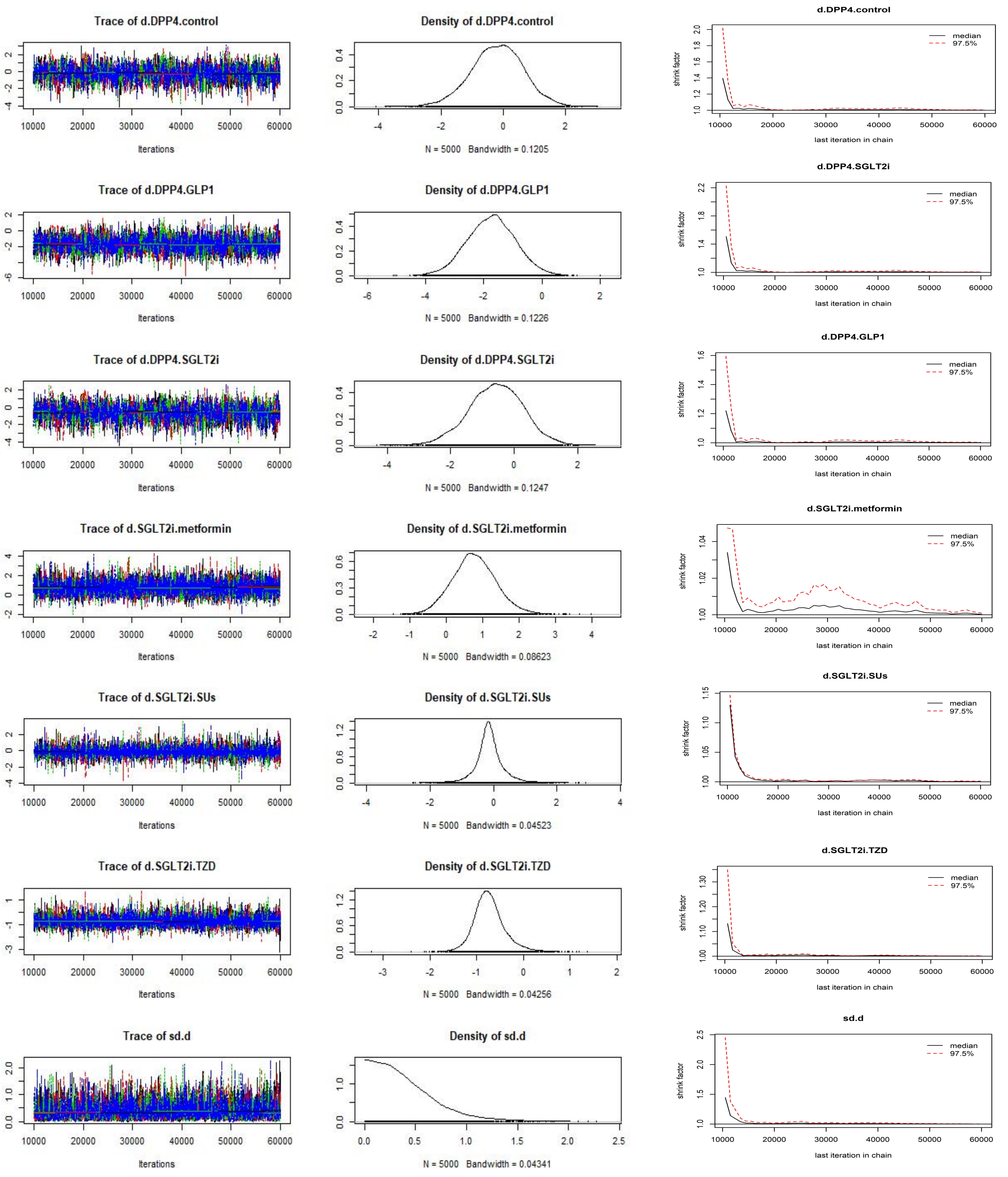

Fig.s2 VAT

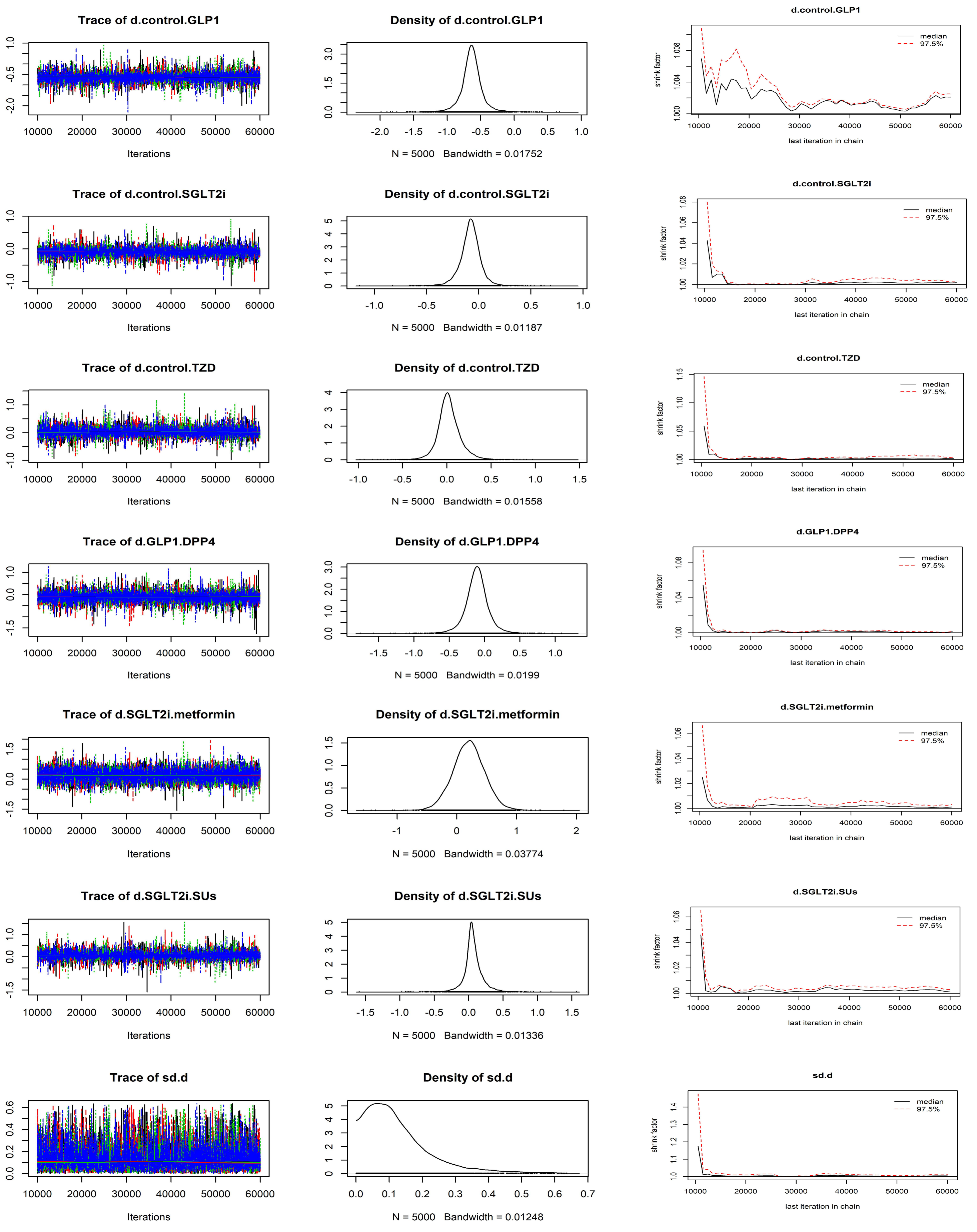
Trace of d.control.GLP1
Density of d.control.GLP1
d.control.GLP1
Iterations
N = 5000 Bandwidth = 0.01752
shrink factor
last iteration in chain
median
97.5%
Trace of d.control.SGLT2i
Density of d.control.SGLT2i
d.control.SGLT2i
N = 5000 Bandwidth = 0.01187
Trace of d.control.TZD
Density of d.control.TZD
d.control.TZD
N = 5000 Bandwidth = 0.01558
Trace of d.GLP1.DPP4
Density of d.GLP1.DPP4
d.GLP1.DPP4
N = 5000 Bandwidth = 0.0199
Trace of d.SGLT2i.metformin
Density of d.SGLT2i.metformin
d.SGLT2i.metformin
N = 5000 Bandwidth = 0.03774
Trace of d.SGLT2i.SUs
Density of d.SGLT2i.SUs
d.SGLT2i.SUs
N = 5000 Bandwidth = 0.01336
Trace of sd.d
Density of sd.d
sd.d
N = 5000 Bandwidth = 0.01248

Fig.s3 SAT

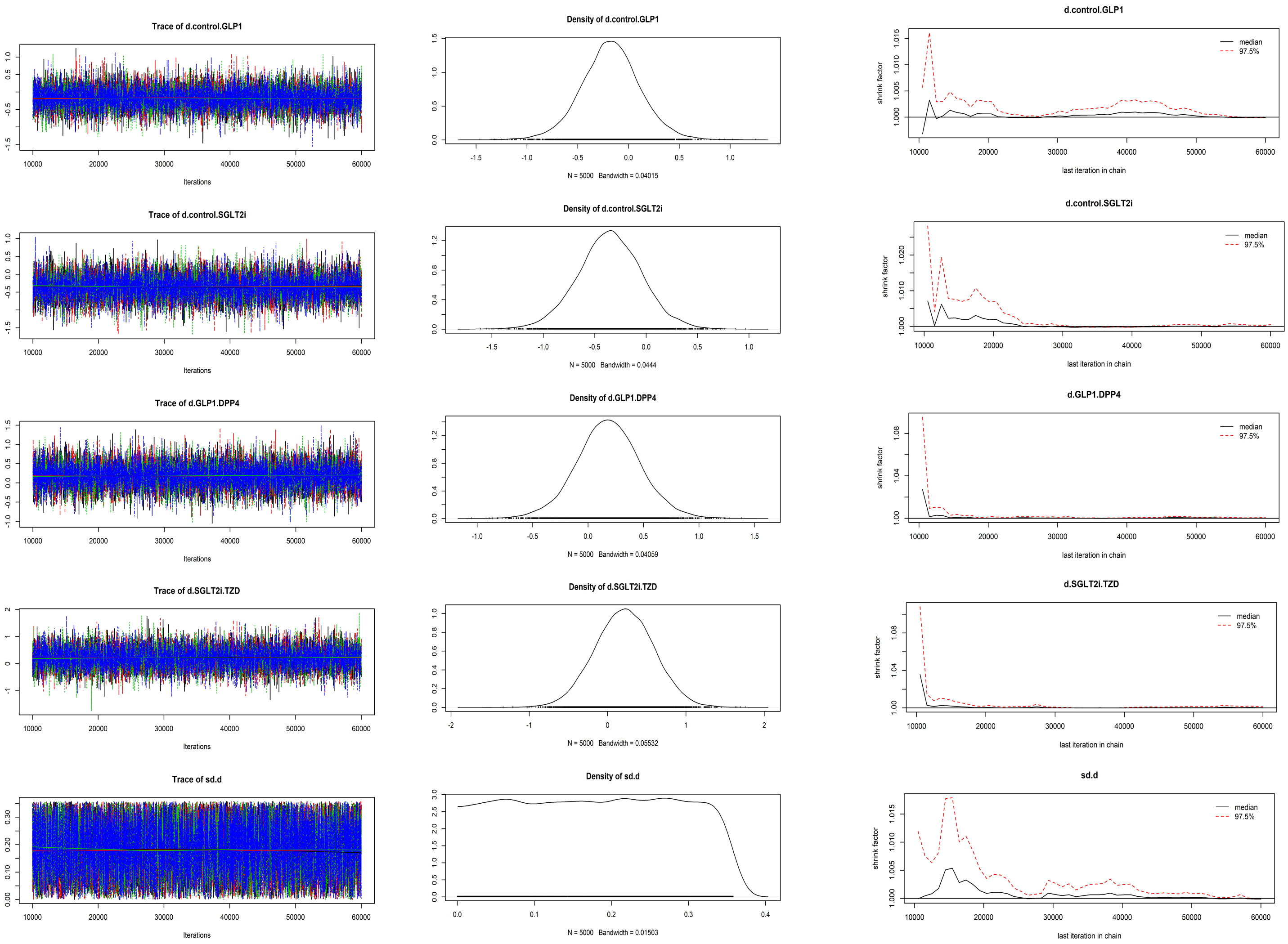

Trace of d.control.GLP1
Iterations
Density of d.control.GLP1
N = 5000 Bandwidth = 0.04015
d.control.GLP1
shrink factor
median
97.5%
last iteration in chain
Trace of d.control.SGLT2i
Iterations
Density of d.control.SGLT2i
N = 5000 Bandwidth = 0.0444
d.control.SGLT2i
shrink factor
median
97.5%
last iteration in chain
Trace of d.GLP1.DPP4
Iterations
Density of d.GLP1.DPP4
N = 5000 Bandwidth = 0.04059
d.GLP1.DPP4
shrink factor
median
97.5%
last iteration in chain
Trace of d.SGLT2i.TZD
Iterations
Density of d.SGLT2i.TZD
N = 5000 Bandwidth = 0.05532
d.SGLT2i.TZD
shrink factor
median
97.5%
last iteration in chain
Trace of sd.d
Iterations
Density of sd.d
N = 5000 Bandwidth = 0.01503
sd.d
shrink factor
median
97.5%
last iteration in chain

Fig.s4 BMI

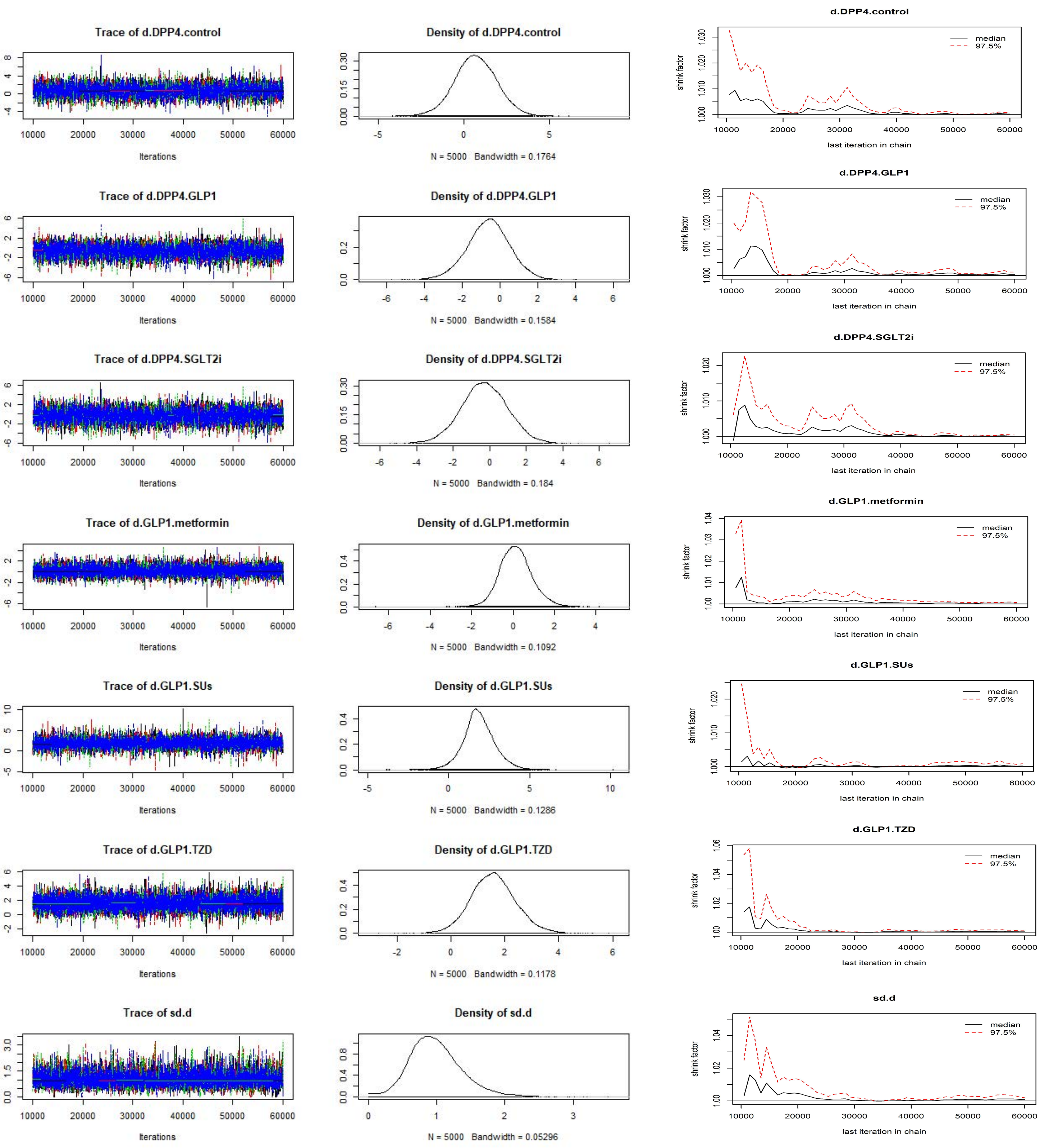
Trace of d.DPP4.control
Iterations
Density of d.DPP4.control
N = 5000 Bandwidth = 0.1764
d.DPP4.control
shrink factor
median
97.5%
last iteration in chain
Trace of d.DPP4.GLP1
Iterations
Density of d.DPP4.GLP1
N = 5000 Bandwidth = 0.1584
d.DPP4.GLP1
shrink factor
median
97.5%
last iteration in chain
Trace of d.DPP4.SGLT2i
Iterations
Density of d.DPP4.SGLT2i
N = 5000 Bandwidth = 0.184
d.DPP4.SGLT2i
shrink factor
median
97.5%
last iteration in chain
Trace of d.GLP1.metformin
Iterations
Density of d.GLP1.metformin
N = 5000 Bandwidth = 0.1092
d.GLP1.metformin
shrink factor
median
97.5%
last iteration in chain
Trace of d.GLP1.SUs
Iterations
Density of d.GLP1.SUs
N = 5000 Bandwidth = 0.1286
d.GLP1.SUs
shrink factor
median
97.5%
last iteration in chain
Trace of d.GLP1.TZD
Iterations
Density of d.GLP1.TZD
N = 5000 Bandwidth = 0.1178
d.GLP1.TZD
shrink factor
median
97.5%
last iteration in chain
Trace of sd.d
Iterations
Density of sd.d
N = 5000 Bandwidth = 0.05296
sd.d
shrink factor
median
97.5%
last iteration in chain

Fig.s5 Weight

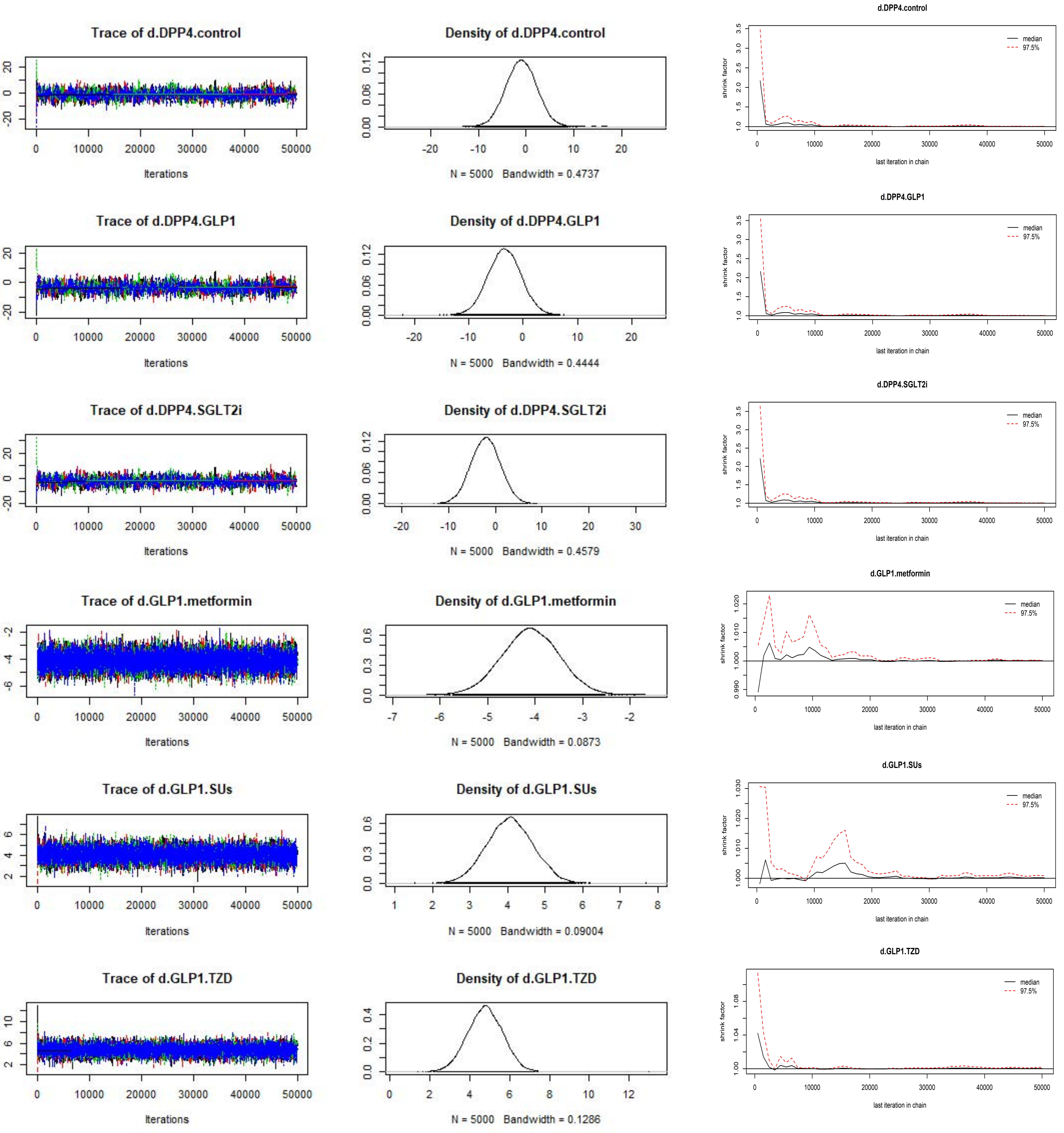

Fig.s6 Leptin

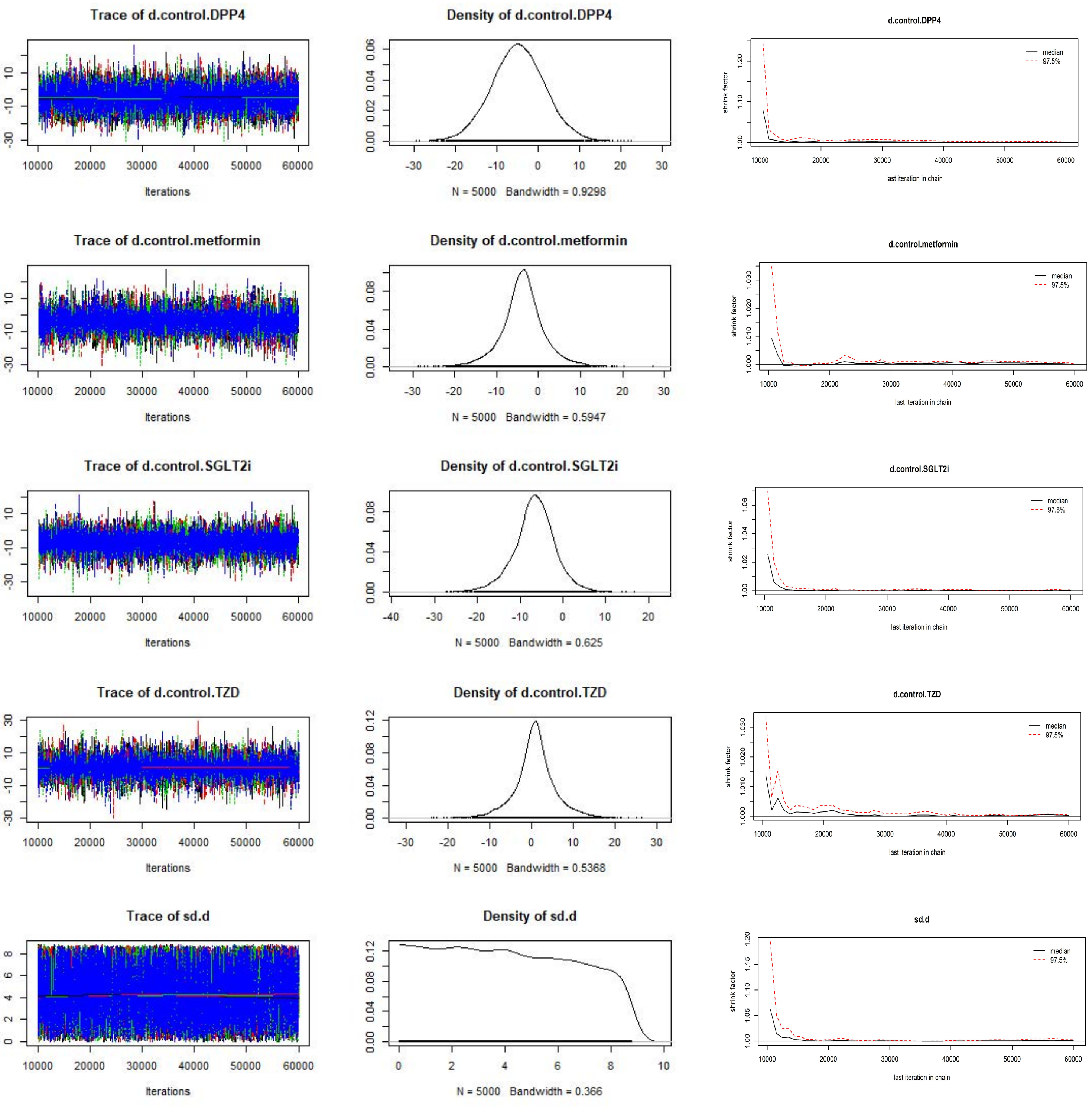

Fig.s7 Adiponectin

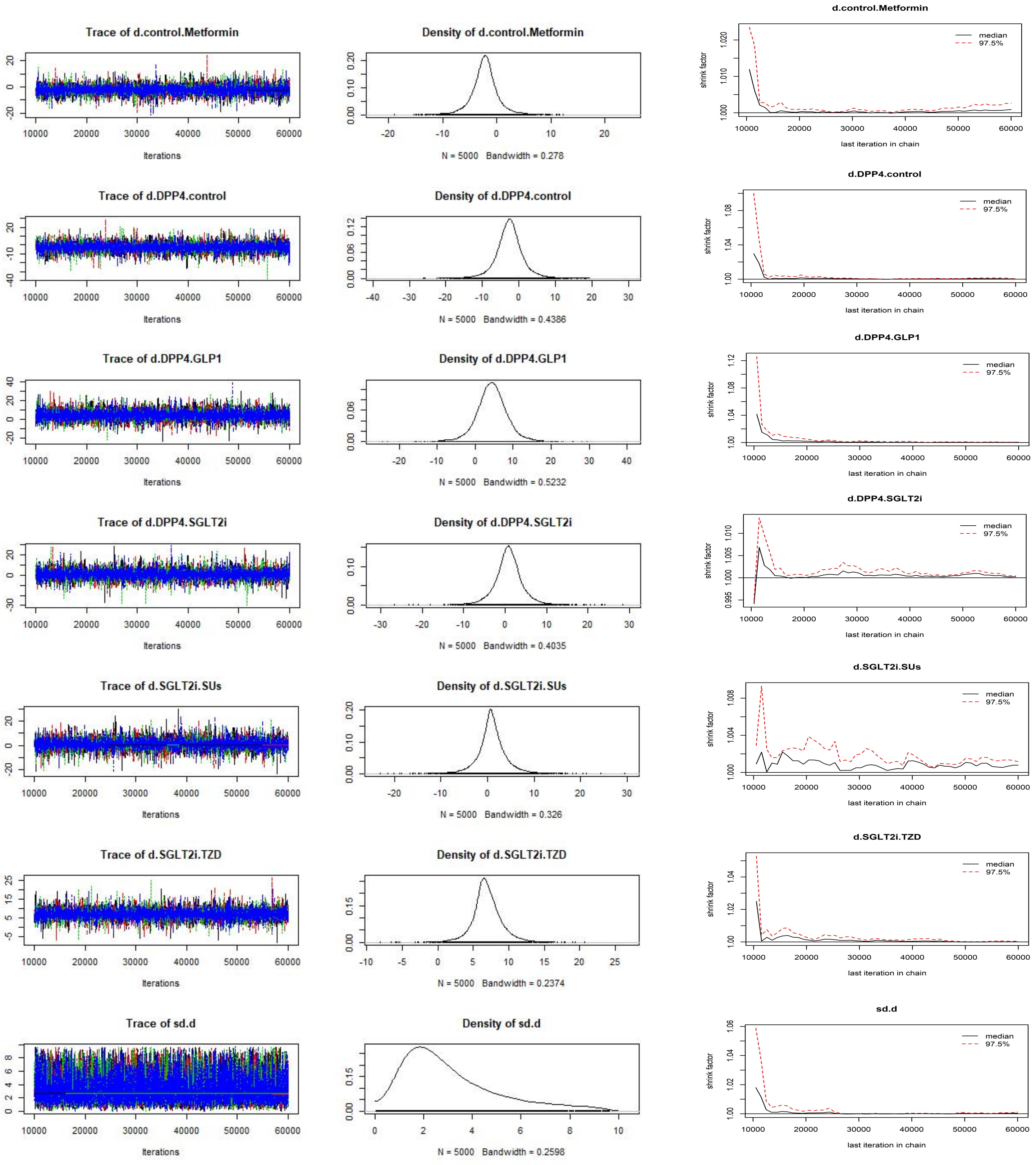

Fig.s8 FBS

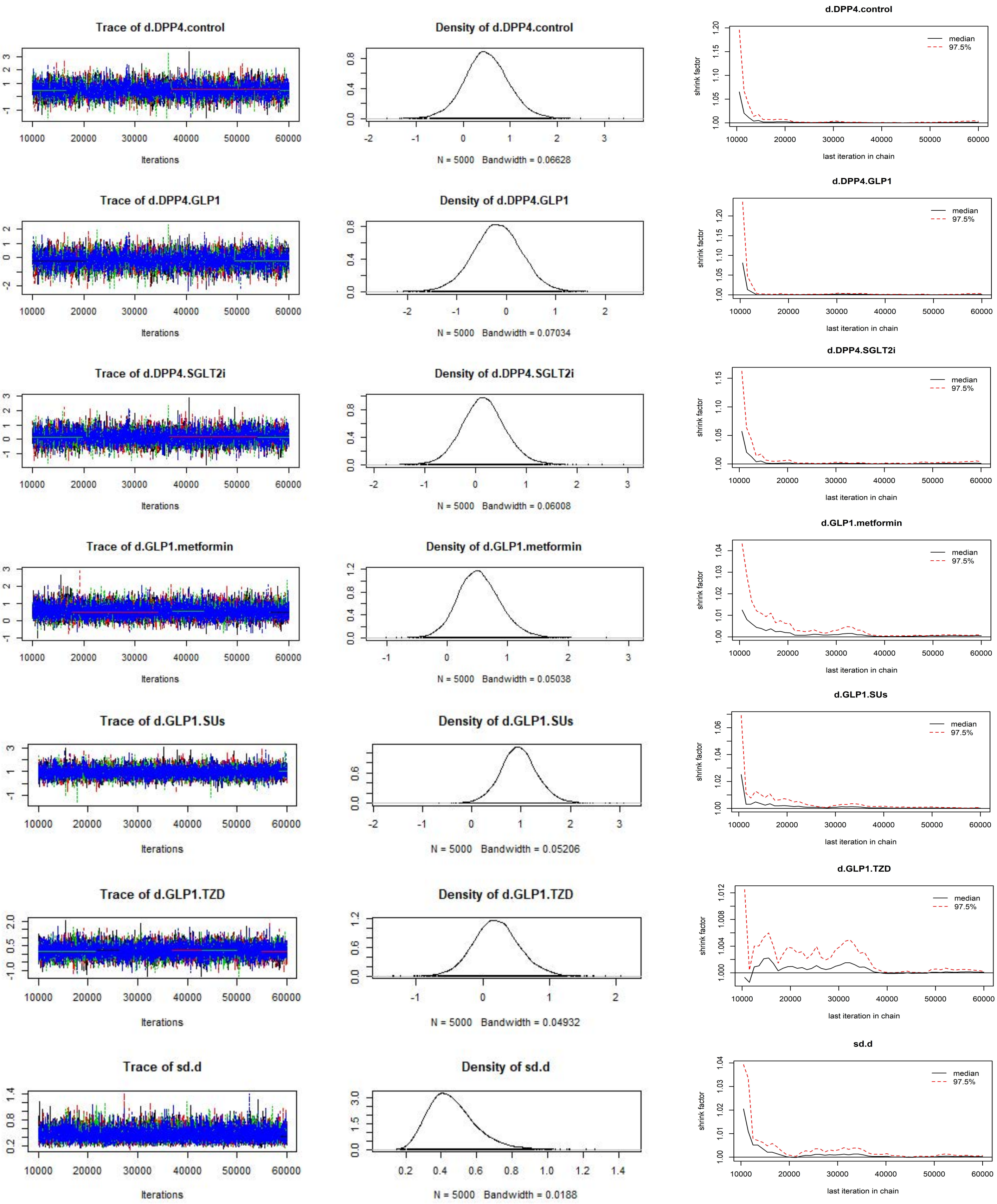
Trace of d.DPP4.control
Iterations
Density of d.DPP4.control
N = 5000 Bandwidth = 0.06628
d.DPP4.control
shrink factor
median
97.5%
last iteration in chain
Trace of d.DPP4.GLP1
Iterations
Density of d.DPP4.GLP1
N = 5000 Bandwidth = 0.07034
d.DPP4.GLP1
shrink factor
median
97.5%
last iteration in chain
Trace of d.DPP4.SGLT2i
Iterations
Density of d.DPP4.SGLT2i
N = 5000 Bandwidth = 0.06008
d.DPP4.SGLT2i
shrink factor
median
97.5%
last iteration in chain
Trace of d.GLP1.metformin
Iterations
Density of d.GLP1.metformin
N = 5000 Bandwidth = 0.05038
d.GLP1.metformin
shrink factor
median
97.5%
last iteration in chain
Trace of d.GLP1.SUs
Iterations
Density of d.GLP1.SUs
N = 5000 Bandwidth = 0.05206
d.GLP1.SUs
shrink factor
median
97.5%
last iteration in chain
Trace of d.GLP1.TZD
Iterations
Density of d.GLP1.TZD
N = 5000 Bandwidth = 0.04932
d.GLP1.TZD
shrink factor
median
97.5%
last iteration in chain
Trace of sd.d
Iterations
Density of sd.d
N = 5000 Bandwidth = 0.0188
sd.d
shrink factor
median
97.5%
last iteration in chain

Fig.s9 TG

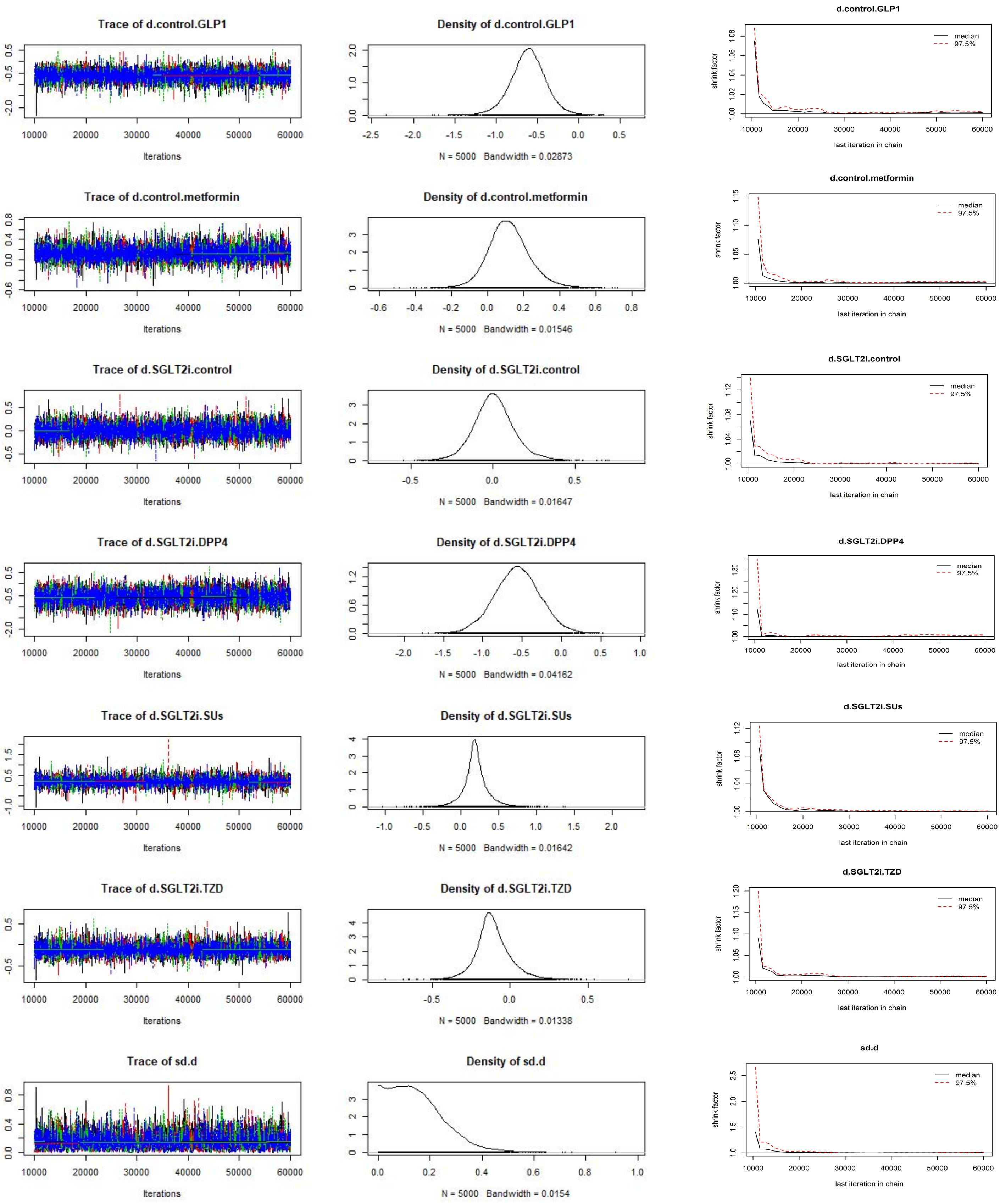

Trace of d.control.GLP1
Density of d.control.GLP1
N = 5000 Bandwidth = 0.02873
d.control.GLP1
Trace of d.control.metformin
Density of d.control.metformin
N = 5000 Bandwidth = 0.01546
d.control.metformin
Trace of d.SGLT2i.control
Density of d.SGLT2i.control
N = 5000 Bandwidth = 0.01647
d.SGLT2i.control
Trace of d.SGLT2i.DPP4
Density of d.SGLT2i.DPP4
N = 5000 Bandwidth = 0.04162
d.SGLT2i.DPP4
Trace of d.SGLT2i.SUs
Density of d.SGLT2i.SUs
N = 5000 Bandwidth = 0.01642
d.SGLT2i.SUs
Trace of d.SGLT2i.TZD
Density of d.SGLT2i.TZD
N = 5000 Bandwidth = 0.01338
d.SGLT2i.TZD
Trace of sd.d
Density of sd.d
N = 5000 Bandwidth = 0.0154
sd.d
Iterations
shrink factor
last iteration in chain
median
97.5%

Fig.s10 TC

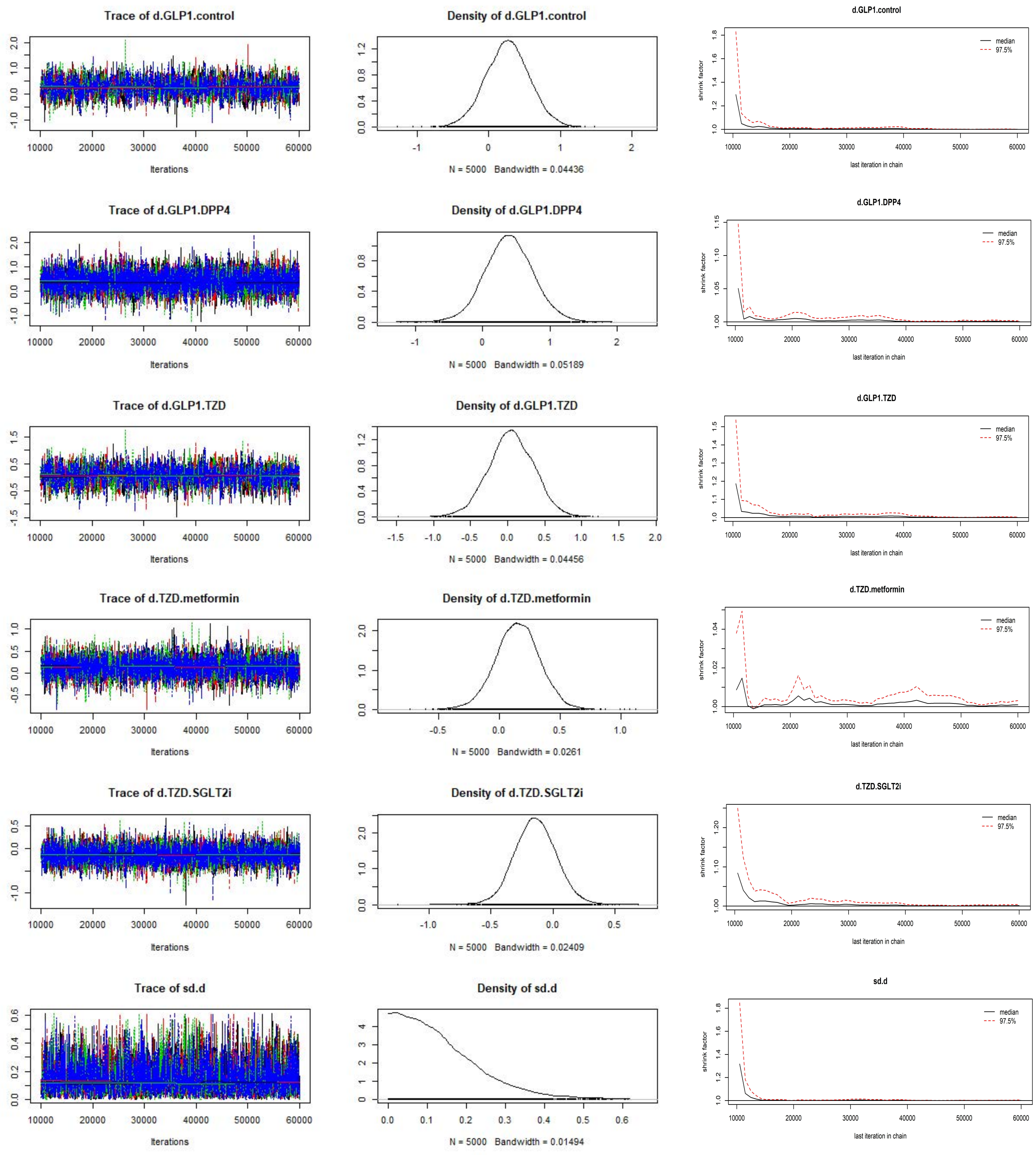

Trace of d.GLP1.control
Density of d.GLP1.control
d.GLP1.control
Trace of d.GLP1.DPP4
Density of d.GLP1.DPP4
d.GLP1.DPP4
Trace of d.GLP1.TZD
Density of d.GLP1.TZD
d.GLP1.TZD
Trace of d.TZD.metformin
Density of d.TZD.metformin
d.TZD.metformin
Trace of d.TZD.SGLT2i
Density of d.TZD.SGLT2i
d.TZD.SGLT2i
Trace of sd.d
Density of sd.d
sd.d
Iterations
N = 5000 Bandwidth = 0.04436
N = 5000 Bandwidth = 0.05189
N = 5000 Bandwidth = 0.04456
N = 5000 Bandwidth = 0.0261
N = 5000 Bandwidth = 0.02409
N = 5000 Bandwidth = 0.01494
shrink factor
last iteration in chain
median
97.5%

Fig.s11 HDL

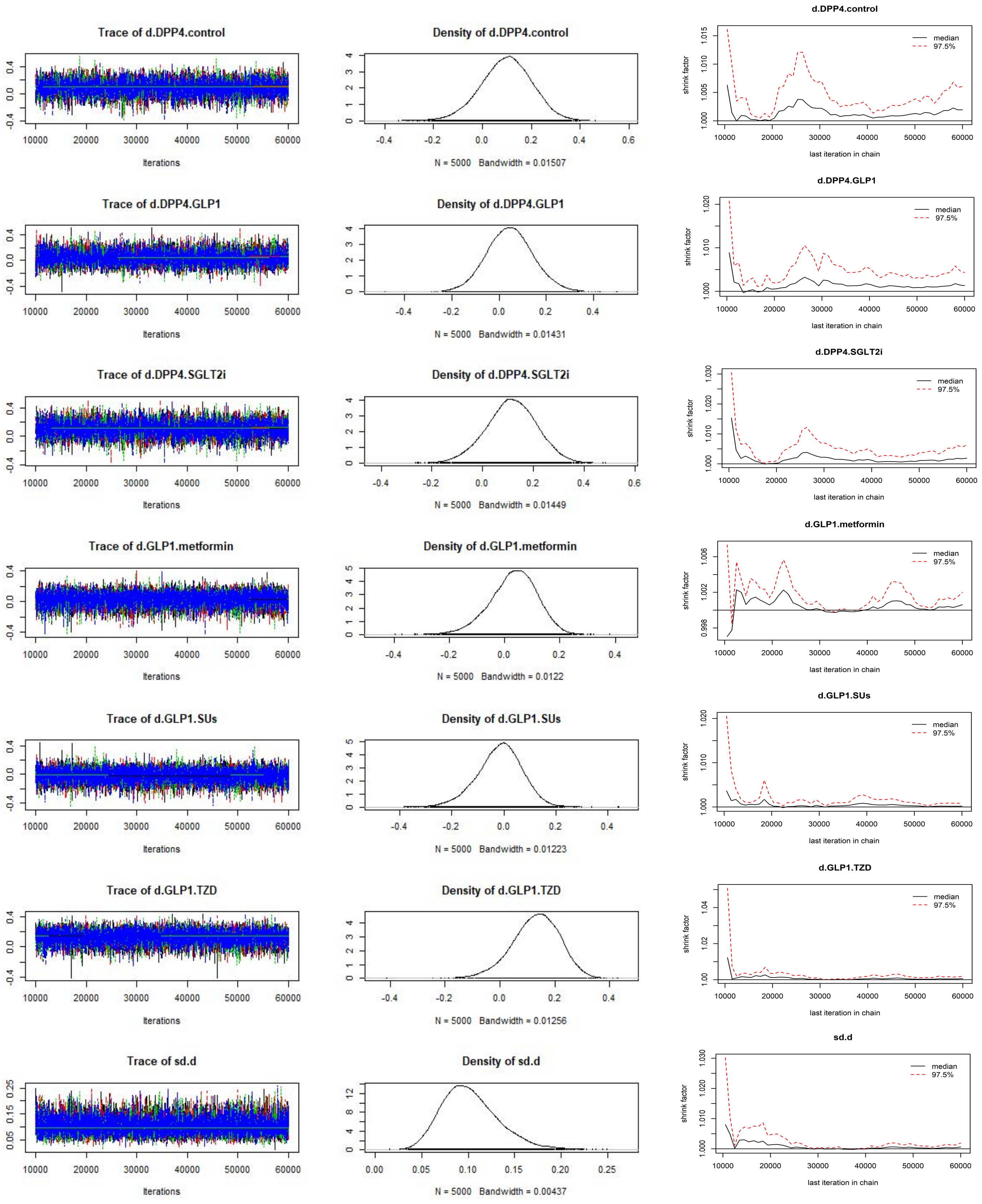

Fig.s12 LDL

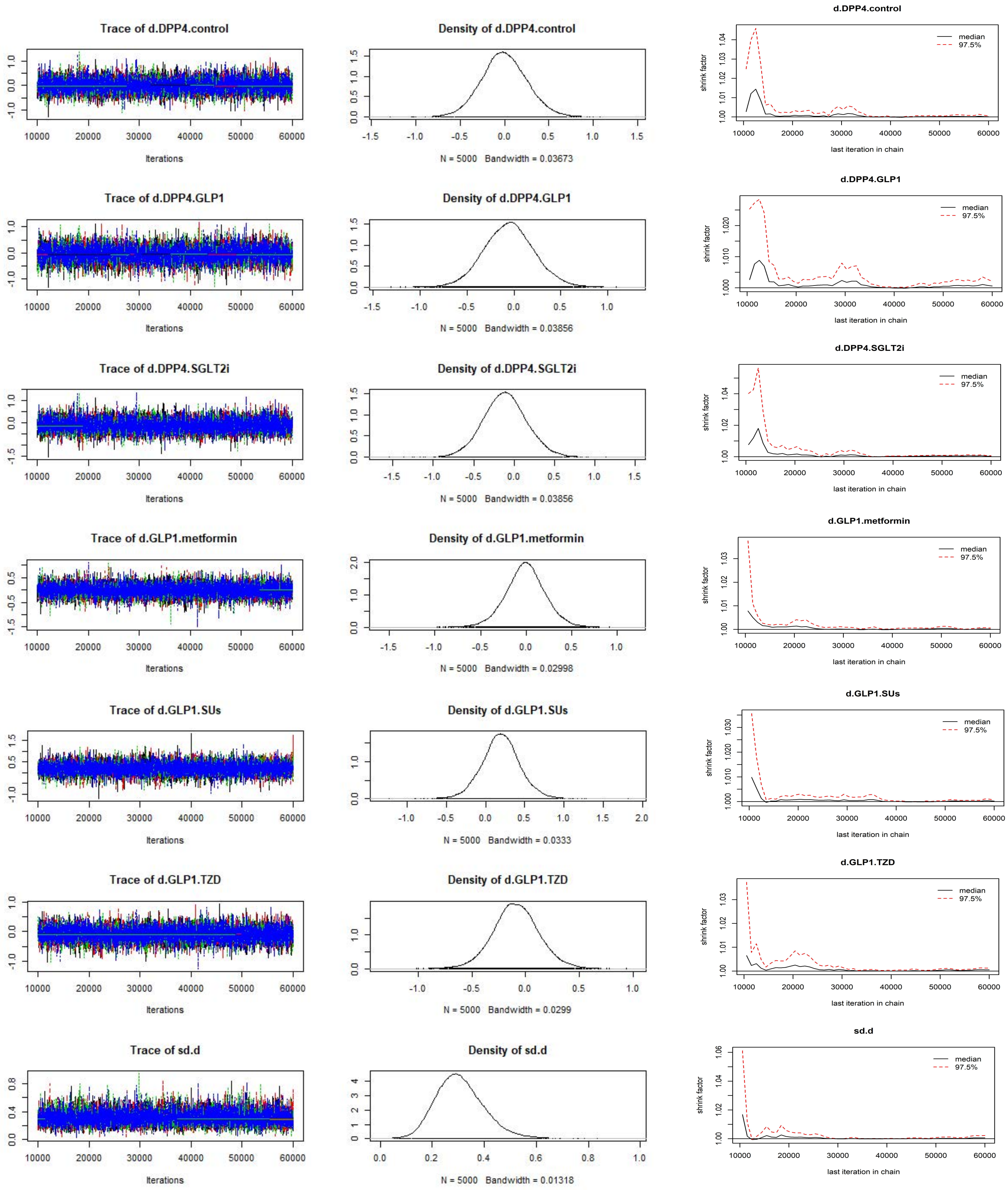
Trace of d.DPP4.control
Iterations
Density of d.DPP4.control
N = 5000 Bandwidth = 0.03673
d.DPP4.control
median
97.5%
shrink factor
last iteration in chain
Trace of d.DPP4.GLP1
Iterations
Density of d.DPP4.GLP1
N = 5000 Bandwidth = 0.03856
d.DPP4.GLP1
median
97.5%
shrink factor
last iteration in chain
Trace of d.DPP4.SGLT2i
Iterations
Density of d.DPP4.SGLT2i
N = 5000 Bandwidth = 0.03856
d.DPP4.SGLT2i
median
97.5%
shrink factor
last iteration in chain
Trace of d.GLP1.metformin
Iterations
Density of d.GLP1.metformin
N = 5000 Bandwidth = 0.02998
d.GLP1.metformin
median
97.5%
shrink factor
last iteration in chain
Trace of d.GLP1.SUs
Iterations
Density of d.GLP1.SUs
N = 5000 Bandwidth = 0.0333
d.GLP1.SUs
median
97.5%
shrink factor
last iteration in chain
Trace of d.GLP1.TZD
Iterations
Density of d.GLP1.TZD
N = 5000 Bandwidth = 0.0299
d.GLP1.TZD
median
97.5%
shrink factor
last iteration in chain
Trace of sd.d
Iterations
Density of sd.d
N = 5000 Bandwidth = 0.01318
sd.d
median
97.5%
shrink factor
last iteration in chain

Fig.s13 SBP

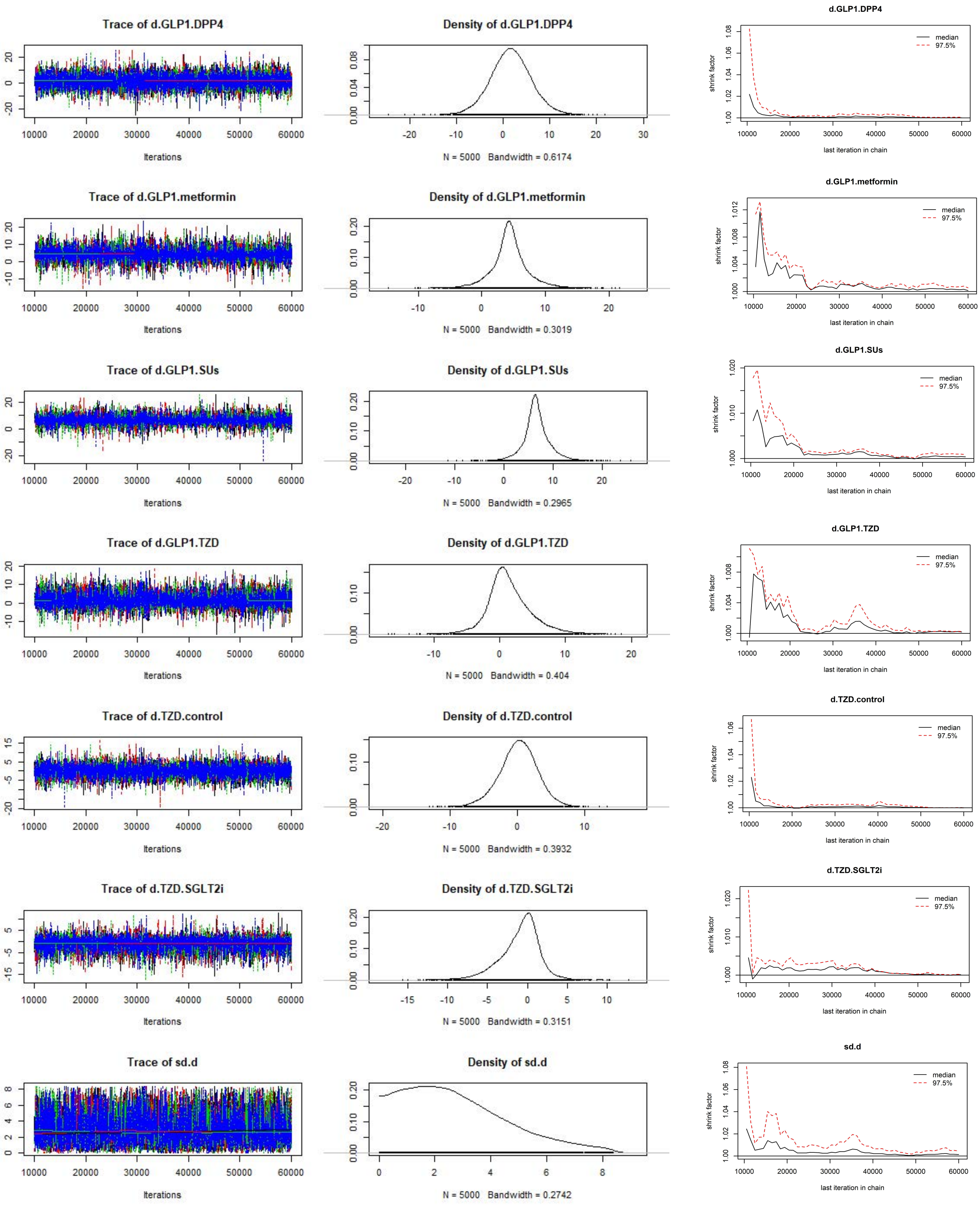

d.GLP1.DPP4
Trace of d.GLP1.DPP4
Density of d.GLP1.DPP4
Iterations
N = 5000 Bandwidth = 0.6174
shrink factor
last iteration in chain
median
97.5%
d.GLP1.metformin
Trace of d.GLP1.metformin
Density of d.GLP1.metformin
N = 5000 Bandwidth = 0.3019
d.GLP1.SUs
Trace of d.GLP1.SUs
Density of d.GLP1.SUs
N = 5000 Bandwidth = 0.2965
d.GLP1.TZD
Trace of d.GLP1.TZD
Density of d.GLP1.TZD
N = 5000 Bandwidth = 0.404
d.TZD.control
Trace of d.TZD.control
Density of d.TZD.control
N = 5000 Bandwidth = 0.3932
d.TZD.SGLT2i
Trace of d.TZD.SGLT2i
Density of d.TZD.SGLT2i
N = 5000 Bandwidth = 0.3151
sd.d
Trace of sd.d
Density of sd.d
N = 5000 Bandwidth = 0.2742

Fig.s14 RBP

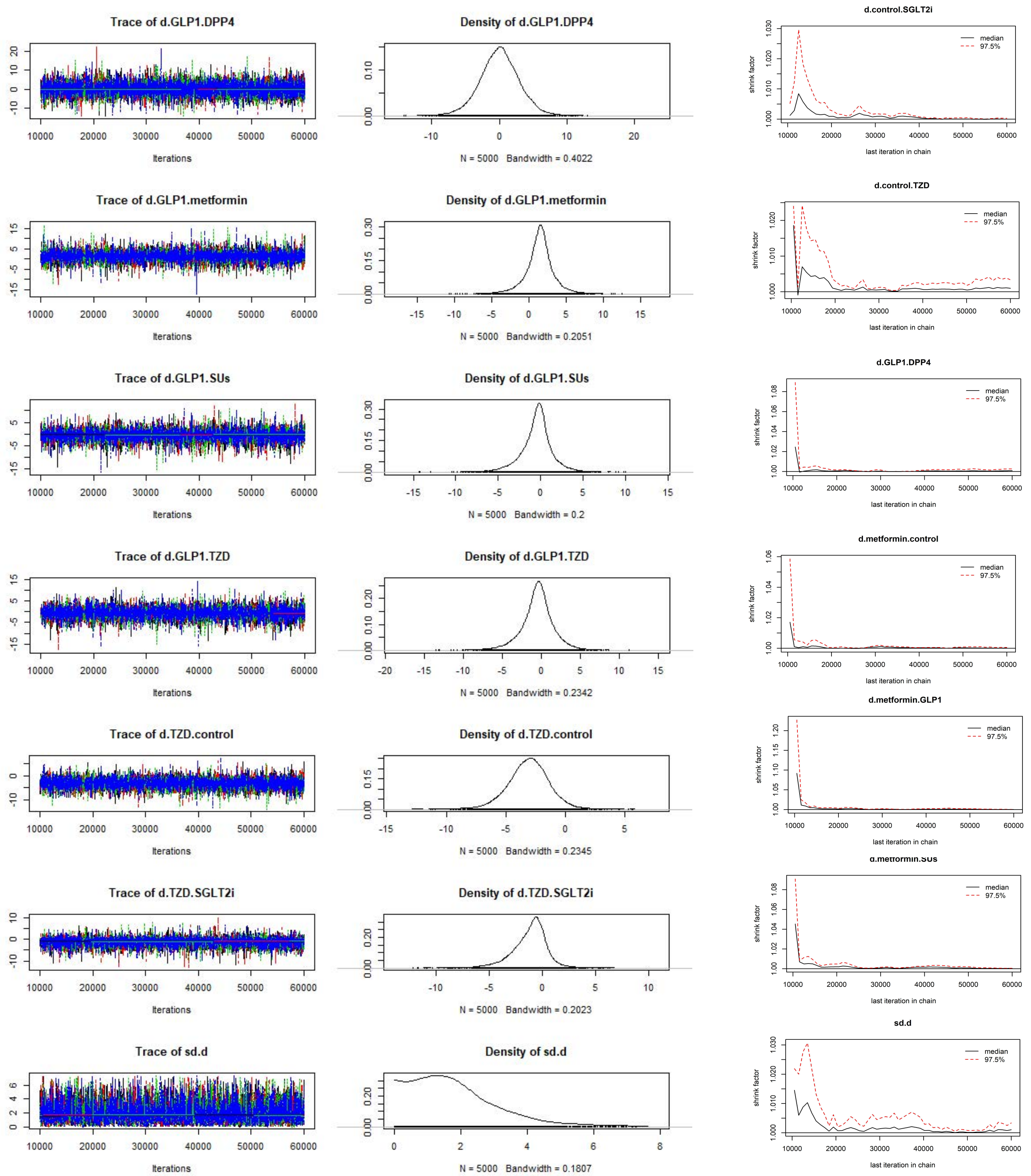

Trace of d.GLP1.DPP4
Iterations
Density of d.GLP1.DPP4
N = 5000 Bandwidth = 0.4022
d.control.SGLT2i
shrink factor
last iteration in chain
median
97.5%
Trace of d.GLP1.metformin
Iterations
Density of d.GLP1.metformin
N = 5000 Bandwidth = 0.2051
d.control.TZD
Trace of d.GLP1.SUs
Density of d.GLP1.SUs
N = 5000 Bandwidth = 0.2
d.GLP1.DPP4
Trace of d.GLP1.TZD
Density of d.GLP1.TZD
N = 5000 Bandwidth = 0.2342
d.metformin.control
d.metformin.GLP1
Trace of d.TZD.control
Density of d.TZD.control
N = 5000 Bandwidth = 0.2345
Trace of d.TZD.SGLT2i
Density of d.TZD.SGLT2i
N = 5000 Bandwidth = 0.2023
Trace of sd.d
Density of sd.d
N = 5000 Bandwidth = 0.1807
sd.d

Fig.s15 AST

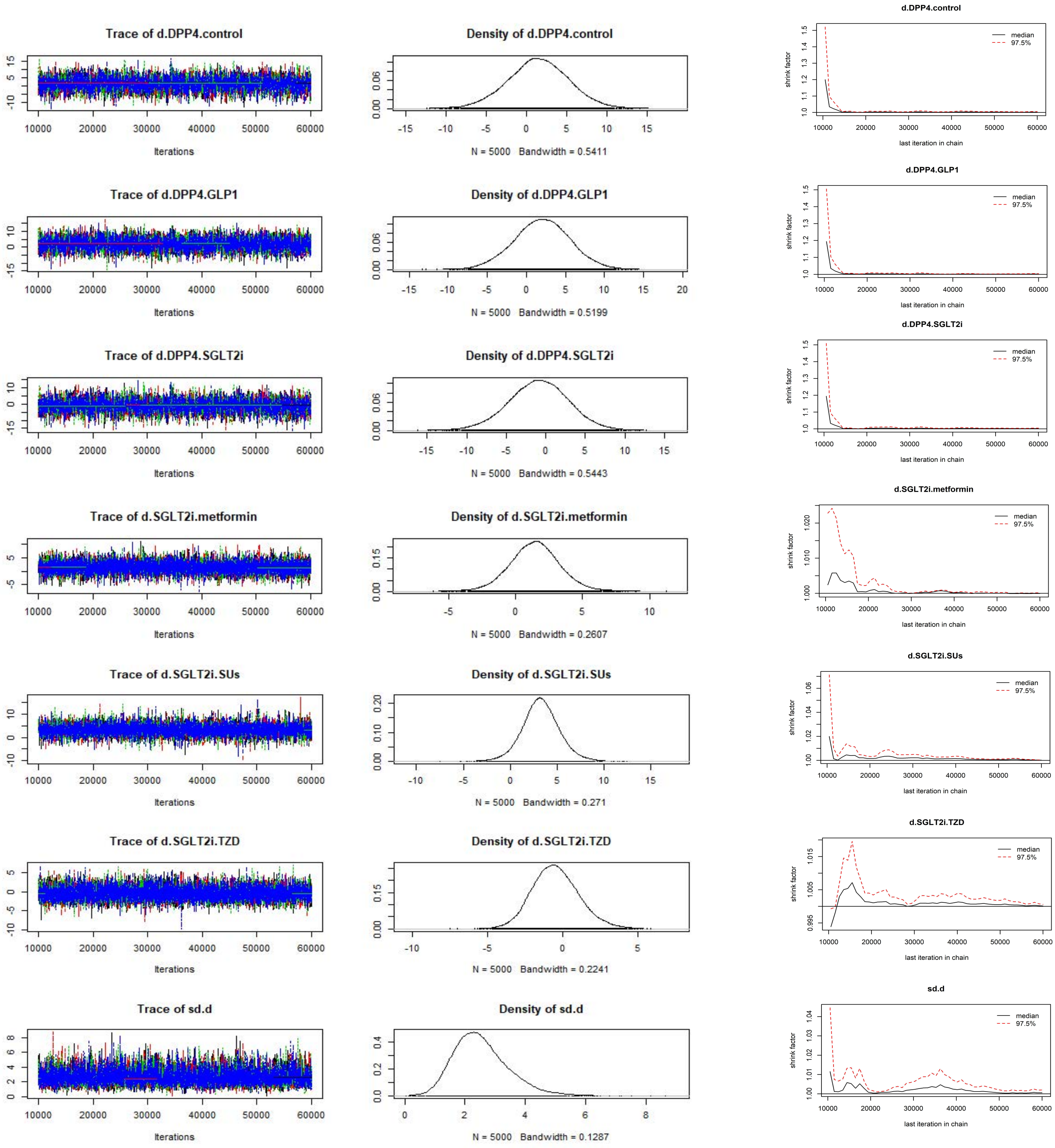

Trace of d.DPP4.control
Iterations
Density of d.DPP4.control
N = 5000 Bandwidth = 0.5411
d.DPP4.control
shrink factor
median
97.5%
last iteration in chain
Trace of d.DPP4.GLP1
Iterations
Density of d.DPP4.GLP1
N = 5000 Bandwidth = 0.5199
d.DPP4.GLP1
Trace of d.DPP4.SGLT2i
Iterations
Density of d.DPP4.SGLT2i
N = 5000 Bandwidth = 0.5443
d.DPP4.SGLT2i
Trace of d.SGLT2i.metformin
Iterations
Density of d.SGLT2i.metformin
N = 5000 Bandwidth = 0.2607
d.SGLT2i.metformin
Trace of d.SGLT2i.SUs
Iterations
Density of d.SGLT2i.SUs
N = 5000 Bandwidth = 0.271
d.SGLT2i.SUs
Trace of d.SGLT2i.TZD
Iterations
Density of d.SGLT2i.TZD
N = 5000 Bandwidth = 0.2241
d.SGLT2i.TZD
Trace of sd.d
Iterations
Density of sd.d
N = 5000 Bandwidth = 0.1287
sd.d

Fig.s16 ALT

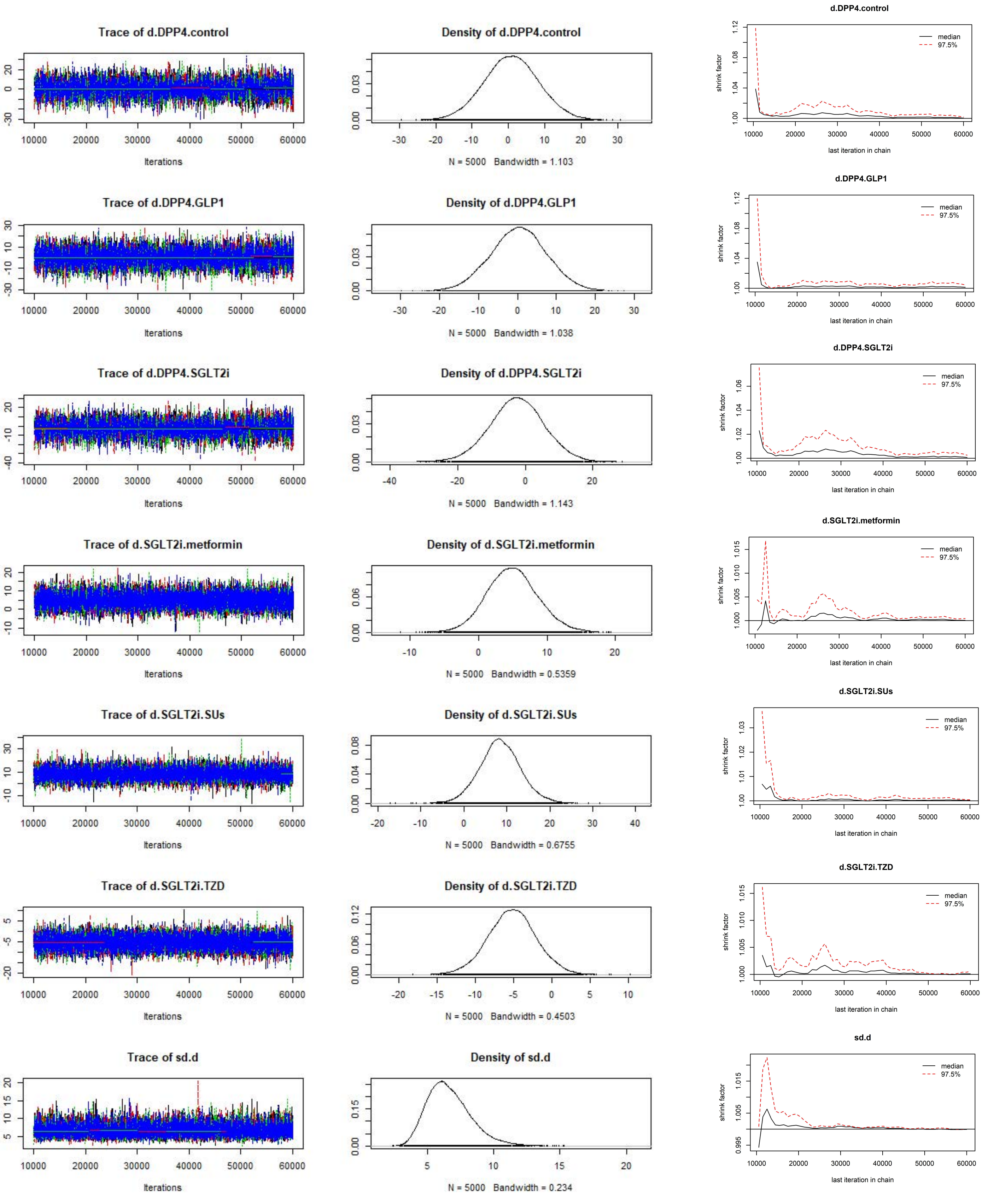
Trace of d.DPP4.control
Iterations
Density of d.DPP4.control
N = 5000 Bandwidth = 1.103
d.DPP4.control
shrink factor
median
97.5%
last iteration in chain
Trace of d.DPP4.GLP1
Iterations
Density of d.DPP4.GLP1
N = 5000 Bandwidth = 1.038
d.DPP4.GLP1
shrink factor
median
97.5%
last iteration in chain
Trace of d.DPP4.SGLT2i
Iterations
Density of d.DPP4.SGLT2i
N = 5000 Bandwidth = 1.143
d.DPP4.SGLT2i
shrink factor
median
97.5%
last iteration in chain
Trace of d.SGLT2i.metformin
Iterations
Density of d.SGLT2i.metformin
N = 5000 Bandwidth = 0.5359
d.SGLT2i.metformin
shrink factor
median
97.5%
last iteration in chain
Trace of d.SGLT2i.SUs
Iterations
Density of d.SGLT2i.SUs
N = 5000 Bandwidth = 0.6755
d.SGLT2i.SUs
shrink factor
median
97.5%
last iteration in chain
Trace of d.SGLT2i.TZD
Iterations
Density of d.SGLT2i.TZD
N = 5000 Bandwidth = 0.4503
d.SGLT2i.TZD
shrink factor
median
97.5%
last iteration in chain
Trace of sd.d
Iterations
Density of sd.d
N = 5000 Bandwidth = 0.234
sd.d
shrink factor
median
97.5%
last iteration in chain

Appendix 8 Network meta-analysis treatment estimates

The columns present the row drug class compared to the column drug class. The rows present the row drug class compared to the column drug class. The effect estimates are expressed as Mean difference and 95% confidence intervals. For example, the Mean Different for HOMA-IR with SGLT-2 inhibitors compared to GLP-1 receptor agonists is 1.217 (95% confidence interval -0.08694 to 2.21). For the reverse, the effect of a GLP-1 receptor compared to SGLT-2 inhibitor on the Mean difference of HOMA-IR is -1.217 (95% confidence interval -2.21 to 0.08694).

1. HOMA-IR

DPP4	-1.763 (-3.346, -0.1302)	-0.5655 (-2.243, 1.049)	-0.6917 (-2.586, 1.1)	-1.299 (-2.952, 0.3708)	-0.2021 (-1.802, 1.379)	0.1973 (-1.75, 2.127)
1.763 (0.1302, 3.346)	GLP1	1.217 (-0.08694, 2.21)	1.091 (-0.4524, 2.336)	0.4713 (-0.6968, 1.503)	1.573 (0.495, 2.523)	1.953 (0.4605, 3.417)
0.5655 (-1.049, 2.243)	-1.217 (-2.21, 0.08694)	SGLT2i	-0.1469 (-1.095, 0.9078)	-0.7547 (-1.367, 0.09404)	0.342 (-0.218, 1.156)	0.7418 (-0.3898, 2.08)
0.6917 (-1.1, 2.586)	-1.091 (-2.336, 0.4524)	0.1469 (-0.9078, 1.095)	SUs	-0.6185 (-1.537, 0.4586)	0.4743 (-0.5714, 1.696)	0.873 (-0.5201, 2.447)
1.299 (-0.3708, 2.952)	-0.4713 (-1.503, 0.6968)	0.7547 (-0.09404, 1.367)	0.6185 (-0.4586, 1.537)	TZD	1.094 (0.3171, 1.911)	1.484 (0.2217, 2.809)
0.2021 (-1.379, 1.802)	-1.573 (-2.523, -0.495)	-0.342 (-1.156, 0.218)	-0.4743 (-1.696, 0.5714)	-1.094 (-1.911, -0.3171)	control	0.3842 (-0.7482, 1.543)
-0.1973 (-2.127, 1.75)	-1.953 (-3.417, -0.4605)	-0.7418 (-2.08, 0.3898)	-0.873 (-2.447, 0.5201)	-1.484 (-2.809, -0.2217)	-0.3842 (-1.543, 0.7482)	metformin

2.1 VAT

DPP4	0.1052 (-0.2597, 0.4755)	0.667 (0.09296, 1.207)	0.7239 (0.1003, 1.366)	0.7592 (0.2121, 1.376)	0.7433 (0.2351, 1.256)	0.8659 (0.09067, 1.617)
-0.1052 (-0.4755, 0.2597)	GLP1	0.5599 (0.1312, 0.9613)	0.6189 (0.1122, 1.143)	0.6541 (0.2396, 1.137)	0.6365 (0.2838, 0.9921)	0.7602 (0.08004, 1.423)
-0.667 (-1.207, -0.09296)	-0.5599 (-0.9613, -0.1312)	SGLT2i	0.05377 (-0.2325, 0.399)	0.09172 (-0.0719, 0.3568)	0.07798 (-0.1199, 0.3076)	0.2019 (-0.3369, 0.7445)
-0.7239 (-1.366, -0.1003)	-0.6189 (-1.143, -0.1122)	-0.05377 (-0.399, 0.2325)	SUs	0.0315 (-0.2537, 0.379)	0.01983 (-0.3592, 0.3817)	0.1401 (-0.4904, 0.7446)
-0.7592 (-1.376, -0.2121)	-0.6541 (-1.137, -0.2396)	-0.09172 (-0.3568, 0.0719)	-0.0315 (-0.379, 0.2537)	TZD	-0.01682 (-0.324, 0.2263)	0.1007 (-0.5022, 0.6573)
-0.7433 (-1.256, -0.2351)	-0.6365 (-0.9921, -0.2838)	-0.07798 (-0.3076, 0.1199)	-0.01983 (-0.3817, 0.3592)	0.01682 (-0.2263, 0.324)	control	0.1225 (-0.4624, 0.6931)
-0.8659 (-1.617, -0.09067)	-0.7602 (-1.423, -0.08004)	-0.2019 (-0.7445, 0.3369)	-0.1401 (-0.7446, 0.4904)	-0.1007 (-0.6573, 0.5022)	-0.1225 (-0.6931, 0.4624)	metformin

2.2 SAT

DPP4	-0.187 (-0.7722, 0.4037)	-0.3708 (-1.401, 0.672)	-0.162 (-1.433, 1.123)	-0.009651 (-0.8366, 0.8209)
0.187 (-0.4037, 0.7722)	GLP1	-0.1841 (-1.03, 0.6687)	0.02507 (-1.097, 1.169)	0.1761 (-0.4034, 0.7581)
0.3708 (-0.672, 1.401)	0.1841 (-0.6687, 1.03)	SGLT2i	0.2102 (-0.5351, 0.9574)	0.3603 (-0.2604, 0.9788)
0.162 (-1.123, 1.433)	-0.02507 (-1.169, 1.097)	-0.2102 (-0.9574, 0.5351)	TZD	0.1493 (-0.8213, 1.118)
0.009651 (-0.8209, 0.8366)	-0.1761 (-0.7581, 0.4034)	-0.3603 (-0.9788, 0.2604)	-0.1493 (-1.118, 0.8213)	control

2.3 BMI

DPP4	-0.5892 (-2.907, 1.574)	-0.295 (-2.805, 2.198)	1.271 (-1.712, 4.158)	0.9481 (-1.557, 3.545)	0.6676 (-1.739, 3.096)	-0.467 (-2.948, 2.067)
0.5892 (-1.574, 2.907)	GLP1	0.3021 (-1.544, 2.305)	1.836 (-0.1381, 4.009)	1.557 (-0.03525, 3.335)	1.262 (-0.2183, 2.933)	0.1171 (-1.329, 1.845)
0.295 (-2.198, 2.805)	-0.3021 (-2.305, 1.544)	SGLT2i	1.557 (-0.9426, 3.971)	1.242 (-0.4719, 2.998)	0.9637 (-0.4232, 2.385)	-0.1823 (-1.812, 1.523)
-1.271 (-4.158, 1.712)	-1.836 (-4.009, 0.1381)	-1.557 (-3.971, 0.9426)	SUs	-0.3178 (-2.614, 2.087)	-0.6037 (-2.783, 1.693)	-1.756 (-3.737, 0.4048)
-0.9481 (-3.545, 1.557)	-1.557 (-3.335, 0.03525)	-1.242 (-2.998, 0.4719)	0.3178 (-2.087, 2.614)	TZD	-0.2825 (-1.415, 0.8711)	-1.431 (-2.919, 0.11)
-0.6676 (-3.096, 1.739)	-1.262 (-2.933, 0.2183)	-0.9637 (-2.385, 0.4232)	0.6037 (-1.693, 2.783)	0.2825 (-0.8711, 1.415)	control	-1.154 (-2.378, 0.1326)
0.467 (-2.067, 2.948)	-0.1171 (-1.845, 1.329)	0.1823 (-1.523, 1.812)	1.756 (-0.4048, 3.737)	1.431 (-0.11, 2.919)	1.154 (-0.1326, 2.378)	metformin

2.4 Weight

DPP4	-3.401 (-9.386, 2.615)	-2.051 (-8.199, 4.12)	0.6672 (-5.347, 6.746)	1.372 (-4.761, 7.528)	-1.006 (-7.241, 5.326)	-7.488 (-13.65, -1.429)
3.401 (-2.615, 9.386)	GLP1	1.327 (-0.4301, 3.062)	4.065 (2.868, 5.263)	4.783 (3.037, 6.469)	2.394 (0.1637, 4.625)	-4.094 (-5.274, -2.933)
2.051 (-4.12, 8.199)	-1.327 (-3.062, 0.4301)	SGLT2i	2.735 (1.37, 4.079)	3.439 (2.051, 4.851)	1.059 (-0.9306, 3.056)	-5.425 (-7.299, -3.579)
-0.6672 (-6.746, 5.347)	-4.065 (-5.263, -2.868)	-2.735 (-4.079, -1.37)	SUs	0.7122 (-0.6773, 2.093)	-1.67 (-3.715, 0.4013)	-8.162 (-9.539, -6.819)
-1.372 (-7.528, 4.761)	-4.783 (-6.469, -3.037)	-3.439 (-4.851, -2.051)	-0.7122 (-2.093, 0.6773)	TZD	-2.39 (-4.206, -0.5505)	-8.874 (-10.74, -7.011)
1.006 (-5.326, 7.241)	-2.394 (-4.625, -0.1637)	-1.059 (-3.056, 0.9306)	1.67 (-0.4013, 3.715)	2.39 (0.5505, 4.206)	control	-6.499 (-8.822, -4.129)
7.488 (1.429, 13.65)	4.094 (2.933, 5.274)	5.425 (3.579, 7.299)	8.162 (6.819, 9.539)	8.874 (7.011, 10.74)	6.499 (4.129, 8.822)	metformin

2.5 Leptin

DPP4	-1.81 (-15.36, 11.16)	5.735 (-11.1, 22.37)	4.768 (-8.161, 17.64)	1.048 (-15.73, 17.94)
1.81 (-11.16, 15.36)	SGLT2i	7.304 (-6.874, 22.9)	6.479 (-3.127, 17.4)	2.754 (-11.77, 18.65)
-5.735 (-22.37, 11.1)	-7.304 (-22.9, 6.874)	TZD	-0.9193 (-11.89, 9.823)	-4.608 (-20.03, 11.24)
-4.768 (-17.64, 8.161)	-6.479 (-17.4, 3.127)	0.9193 (-9.823, 11.89)	control	-3.679 (-14.9, 7.772)
-1.048 (-17.94, 15.73)	-2.754 (-18.65, 11.77)	4.608 (-11.24, 20.03)	3.679 (-7.772, 14.9)	metformin

2.6 Adiponectin

DPP4	4.38 (-4.214, 13.13)	-4.81 (-14.82, 5.14)	0.8245 (-7.204, 8.701)	1.697 (-8.955, 12.57)	7.635 (-1.298, 17.37)	-2.62 (-10.94, 5.638)
-4.38 (-13.13, 4.214)	GLP1	-9.2 (-22.58, 3.954)	-3.575 (-15.34, 8.045)	-2.669 (-16.26, 10.86)	3.283 (-9.084, 16.23)	-7.007 (-18.85, 5.033)
4.81 (-5.14, 14.82)	9.2 (-3.954, 22.58)	Metformin	5.639 (-6.919, 18.36)	6.512 (-7.702, 21.28)	12.44 (-0.676, 26.24)	2.192 (-3.573, 7.773)
-0.8245 (-8.701, 7.204)	3.575 (-8.045, 15.34)	-5.639 (-18.36, 6.919)	SGLT2i	0.8563 (-5.998, 8.19)	6.813 (2.403, 11.78)	-3.402 (-14.67, 7.91)
-1.697 (-12.57, 8.955)	2.669 (-10.86, 16.26)	-6.512 (-21.28, 7.702)	-0.8563 (-8.19, 5.998)	SUs	5.927 (-1.114, 13.14)	-4.311 (-18.1, 8.959)
-7.635 (-17.37, 1.298)	-3.283 (-16.23, 9.084)	-12.44 (-26.24, 0.676)	-6.813 (-11.78, -2.403)	-5.927 (-13.14, 1.114)	TZD	-10.23 (-22.94, 1.637)
2.62 (-5.638, 10.94)	7.007 (-5.033, 18.85)	-2.192 (-7.773, 3.573)	3.402 (-7.91, 14.67)	4.311 (-8.959, 18.1)	10.23 (-1.637, 22.94)	control

3.1 FBS

DPP4	-0.1763 (-1.174, 0.7811)	0.1568 (-0.6753, 1.042)	0.7721 (-0.2653, 1.851)	0.01933 (-0.9075, 0.9964)	0.4836 (-0.4524, 1.455)	0.3421 (-0.5862, 1.378)
0.1763 (-0.7811, 1.174)	GLP1	0.3333 (-0.371, 1.106)	0.9475 (0.2163, 1.743)	0.1969 (-0.4768, 0.934)	0.6627 (0.02059, 1.377)	0.5138 (-0.1253, 1.285)
-0.1568 (-1.042, 0.6753)	-0.3333 (-1.106, 0.371)	SGLT2i	0.6151 (-0.08719, 1.302)	-0.1353 (-0.6372, 0.3551)	0.33 (-0.1701, 0.8322)	0.1836 (-0.3608, 0.7644)
-0.7721 (-1.851, 0.2653)	-0.9475 (-1.743, -0.2163)	-0.6151 (-1.302, 0.08719)	SUs	-0.7511 (-1.433, -0.07856)	-0.2872 (-0.9688, 0.4084)	-0.435 (-1.084, 0.2972)
-0.01933 (-0.9964, 0.9075)	-0.1969 (-0.934, 0.4768)	0.1353 (-0.3551, 0.6372)	0.7511 (0.07856, 1.433)	TZD	0.4634 (0.04479, 0.9085)	0.3161 (-0.1644, 0.8742)
-0.4836 (-1.455, 0.4524)	-0.6627 (-1.377, -0.02059)	-0.33 (-0.8322, 0.1701)	0.2872 (-0.4084, 0.9688)	-0.4634 (-0.9085, -0.04479)	control	-0.1463 (-0.551, 0.3032)
-0.3421 (-1.378, 0.5862)	-0.5138 (-1.285, 0.1253)	-0.1836 (-0.7644, 0.3608)	0.435 (-0.2972, 1.084)	-0.3161 (-0.8742, 0.1644)	0.1463 (-0.3032, 0.551)	metformin

3.2 TG

DPP4	-0.04466 (-0.6562, 0.5777)	0.5624 (-0.00302, 1.14)	0.7538 (0.112, 1.423)	0.4482 (-0.1348, 1.054)	0.5682 (0.005976, 1.149)	0.6897 (0.08952, 1.312)
0.04466 (-0.5777, 0.6562)	GLP1	0.6065 (0.1166, 1.095)	0.8007 (0.2218, 1.389)	0.4903 (0.01954, 1.003)	0.6097 (0.1879, 1.056)	0.7261 (0.2626, 1.245)
-0.5624 (-1.14, 0.00302)	-0.6065 (-1.095, -0.1166)	SGLT2i	0.1886 (-0.1544, 0.5722)	-0.1201 (-0.3266, 0.1461)	0.002603 (-0.2344, 0.2785)	0.117 (-0.1777, 0.4962)
-0.7538 (-1.423, -0.112)	-0.8007 (-1.389, -0.2218)	-0.1886 (-0.5722, 0.1544)	SUs	-0.3111 (-0.6563, 0.07642)	-0.1902 (-0.5851, 0.2227)	-0.07766 (-0.4987, 0.4179)
-0.4482 (-1.054, 0.1348)	-0.4903 (-1.003, -0.01954)	0.1201 (-0.1461, 0.3266)	0.3111 (-0.07642, 0.6563)	TZD	0.118 (-0.1306, 0.3521)	0.2335 (-0.05576, 0.5534)
-0.5682 (-1.149, -0.005976)	-0.6097 (-1.056, -0.1879)	-0.002603 (-0.2785, 0.2344)	0.1902 (-0.2227, 0.5851)	-0.118 (-0.3521, 0.1306)	control	0.1146 (-0.09777, 0.3733)
-0.6897 (-1.312, -0.08952)	-0.7261 (-1.245, -0.2626)	-0.117 (-0.4962, 0.1777)	0.07766 (-0.4179, 0.4987)	-0.2335 (-0.5534, 0.05576)	-0.1146 (-0.3733, 0.09777)	metformin

3.3 TC

DPP4	-0.3912 (-1.116, 0.3134)	-0.484 (-1.47, 0.4763)	-0.3431 (-1.298, 0.5691)	-0.1282 (-1.077, 0.779)	-0.1924 (-1.166, 0.7277)
0.3912 (-0.3134, 1.116)	GLP1	-0.08991 (-0.7504, 0.5684)	0.04941 (-0.5642, 0.6399)	0.2627 (-0.3442, 0.8718)	0.2002 (-0.45, 0.8274)
0.484 (-0.4763, 1.47)	0.08991 (-0.5684, 0.7504)	SGLT2i	0.143 (-0.1926, 0.4692)	0.3536 (-0.03457, 0.7543)	0.2944 (-0.1616, 0.7157)
0.3431 (-0.5691, 1.298)	-0.04941 (-0.6399, 0.5642)	-0.143 (-0.4692, 0.1926)	TZD	0.2129 (-0.1043, 0.5528)	0.1495 (-0.2266, 0.5057)
0.1282 (-0.779, 1.077)	-0.2627 (-0.8718, 0.3442)	-0.3536 (-0.7543, 0.03457)	-0.2129 (-0.5528, 0.1043)	control	-0.05701 (-0.3202, 0.1351)
0.1924 (-0.7277, 1.166)	-0.2002 (-0.8274, 0.45)	-0.2944 (-0.7157, 0.1616)	-0.1495 (-0.5057, 0.2266)	0.05701 (-0.1351, 0.3202)	metformin

3.4 HDL

DPP4	0.04482 (-0.1457, 0.2488)	0.1151 (-0.09226, 0.3096)	0.03138 (-0.2034, 0.2525)	0.1793 (-0.04081, 0.3779)	0.1008 (-0.1131, 0.2949)	0.08238 (-0.1464, 0.2874)
-0.04482 (-0.2488, 0.1457)	GLP1	0.07226 (-0.1192, 0.2283)	-0.01163 (-0.203, 0.1449)	0.1351 (-0.05672, 0.2865)	0.05646 (-0.1292, 0.2042)	0.03753 (-0.1516, 0.185)
-0.1151 (-0.3096, 0.09226)	-0.07226 (-0.2283, 0.1192)	SGLT2i	-0.08456 (-0.2424, 0.07509)	0.06453 (-0.05244, 0.1656)	-0.01472 (-0.1334, 0.09208)	-0.0339 (-0.1828, 0.1051)
-0.03138 (-0.2525, 0.2034)	0.01163 (-0.1449, 0.203)	0.08456 (-0.07509, 0.2424)	SUs	0.1503 (-0.01477, 0.2926)	0.07127 (-0.09585, 0.2186)	0.05087 (-0.1151, 0.2028)
-0.1793 (-0.3779, 0.04081)	-0.1351 (-0.2865, 0.05672)	-0.06453 (-0.1656, 0.05244)	-0.1503 (-0.2926, 0.01477)	TZD	-0.07995 (-0.1728, 0.01963)	-0.09939 (-0.2244, 0.03338)
-0.1008 (-0.2949, 0.1131)	-0.05646 (-0.2042, 0.1292)	0.01472 (-0.09208, 0.1334)	-0.07127 (-0.2186, 0.09585)	0.07995 (-0.01963, 0.1728)	control	-0.01946 (-0.1279, 0.09052)
-0.08238 (-0.2874, 0.1464)	-0.03753 (-0.185, 0.1516)	0.0339 (-0.1051, 0.1828)	-0.05087 (-0.2028, 0.1151)	0.09939 (-0.03338, 0.2244)	0.01946 (-0.09052, 0.1279)	metformin

3.5 LDL

DPP4	-0.0507 (-0.58, 0.4943)	-0.1132 (-0.642, 0.4422)	0.1429 (-0.4803, 0.7895)	-0.1405 (-0.6782, 0.4111)	-0.006923 (-0.5067, 0.5297)	-0.05569 (-0.6345, 0.527)
0.0507 (-0.4943, 0.58)	GLP1	-0.06108 (-0.5116, 0.4039)	0.1941 (-0.2946, 0.6866)	-0.09074 (-0.5205, 0.3499)	0.04546 (-0.3552, 0.4657)	-0.003342 (-0.4563, 0.4371)
0.1132 (-0.4422, 0.642)	0.06108 (-0.4039, 0.5116)	SGLT2i	0.2552 (-0.219, 0.7076)	-0.02801 (-0.3419, 0.272)	0.1069 (-0.2049, 0.4214)	0.05784 (-0.3705, 0.4588)
-0.1429 (-0.7895, 0.4803)	-0.1941 (-0.6866, 0.2946)	-0.2552 (-0.7076, 0.219)	SUs	-0.2844 (-0.7336, 0.1792)	-0.1504 (-0.5965, 0.3305)	-0.1977 (-0.6768, 0.2813)
0.1405 (-0.4111, 0.6782)	0.09074 (-0.3499, 0.5205)	0.02801 (-0.272, 0.3419)	0.2844 (-0.1792, 0.7336)	TZD	0.1369 (-0.1408, 0.4292)	0.08749 (-0.3031, 0.462)
0.006923 (-0.5297, 0.5067)	-0.04546 (-0.4657, 0.3552)	-0.1069 (-0.4214, 0.2049)	0.1504 (-0.3305, 0.5965)	-0.1369 (-0.4292, 0.1408)	control	-0.04876 (-0.3874, 0.2553)
0.05569 (-0.527, 0.6345)	0.003342 (-0.4371, 0.4563)	-0.05784 (-0.4588, 0.3705)	0.1977 (-0.2813, 0.6768)	-0.08749 (-0.462, 0.3031)	0.04876 (-0.2553, 0.3874)	metformin

4.1 SBP

DPP4	-1.599 (-10.73, 7.437)	-1.155 (-12.83, 10.68)	4.963 (-5.828, 16.28)	-0.4282 (-10.82, 12.03)	-0.07337 (-11.43, 12.25)	2.725 (-8.298, 14.05)
1.599 (-7.437, 10.73)	GLP1	0.4598 (-7.142, 8.146)	6.502 (0.4226, 13.23)	1.03 (-4.787, 9.146)	1.486 (-5.709, 9.753)	4.323 (-2.135, 11.03)
1.155 (-10.68, 12.83)	-0.4598 (-8.146, 7.142)	SGLT2i	6.019 (0.2878, 12.45)	0.549 (-3.473, 6.993)	1.029 (-4.853, 7.83)	3.861 (-3.332, 11.46)
-4.963 (-16.28, 5.828)	-6.502 (-13.23, -0.4226)	-6.019 (-12.45, -0.2878)	SUs	-5.538 (-10.27, 1.237)	-5.063 (-11.57, 1.993)	-2.196 (-8.437, 3.984)
0.4282 (-12.03, 10.82)	-1.03 (-9.146, 4.787)	-0.549 (-6.993, 3.473)	5.538 (-1.237, 10.27)	TZD	0.2637 (-5.908, 5.795)	3.268 (-4.417, 8.977)
0.07337 (-12.25, 11.43)	-1.486 (-9.753, 5.709)	-1.029 (-7.83, 4.853)	5.063 (-1.993, 11.57)	-0.2637 (-5.795, 5.908)	control	2.811 (-3.965, 8.927)
-2.725 (-14.05, 8.298)	-4.323 (-11.03, 2.135)	-3.861 (-11.46, 3.332)	2.196 (-3.984, 8.437)	-3.268 (-8.977, 4.417)	-2.811 (-8.927, 3.965)	metformin

4.2 DBP

DPP4	-0.05192 (-5.977, 6.035)	-1.499 (-10.19, 5.598)	-0.4318 (-8.099, 6.594)	-0.4508 (-8.021, 6.808)	-3.539 (-11.5, 3.816)	1.436 (-6.108, 8.706)
0.05192 (-6.035, 5.977)	GLP1	-1.311 (-7.328, 2.683)	-0.324 (-4.833, 3.435)	-0.3831 (-5.203, 3.839)	-3.457 (-8.709, 0.8769)	1.505 (-3.067, 5.585)
1.499 (-5.598, 10.19)	1.311 (-2.683, 7.328)	SGLT2i	0.9578 (-2.416, 5.593)	0.955 (-1.713, 5.157)	-1.99 (-5.526, 2.272)	2.841 (-0.8969, 8.17)
0.4318 (-6.594, 8.099)	0.324 (-3.435, 4.833)	-0.9578 (-5.593, 2.416)	SUs	-0.04769 (-3.613, 3.879)	-3.059 (-7.497, 1.12)	1.838 (-1.94, 6.017)
0.4508 (-6.808, 8.021)	0.3831 (-3.839, 5.203)	-0.955 (-5.157, 1.713)	0.04769 (-3.879, 3.613)	TZD	-3.059 (-6.78, 0.3173)	1.896 (-2.366, 6.196)
3.539 (-3.816, 11.5)	3.457 (-0.8769, 8.709)	1.99 (-2.272, 5.526)	3.059 (-1.12, 7.497)	3.059 (-0.3173, 6.78)	control	4.944 (1.389, 8.998)
-1.436 (-8.706, 6.108)	-1.505 (-5.585, 3.067)	-2.841 (-8.17, 0.8969)	-1.838 (-6.017, 1.94)	-1.896 (-6.196, 2.366)	-4.944 (-8.998, -1.389)	metformin

5.1 AST						
DPP4	2.037 (-5.129, 9.062)	-0.8484 (-8.586, 6.503)	2.423 (-5.523, 10.02)	-1.335 (-9.141, 6.31)	1.456 (-6.152, 8.91)	0.5946 (-7.288, 8.002)
-2.037 (-9.062, 5.129)	GLP1	-2.892 (-7.797, 1.816)	0.3425 (-4.342, 5.04)	-3.453 (-8.061, 1.71)	-0.6425 (-4.777, 4.097)	-1.46 (-5.838, 2.93)
0.8484 (-6.503, 8.586)	2.892 (-1.816, 7.797)	SGLT2i	3.224 (-0.8646, 7.554)	-0.5248 (-3.355, 2.908)	2.274 (-0.5881, 5.712)	1.441 (-2.363, 5.282)
-2.423 (-10.02, 5.523)	-0.3425 (-5.04, 4.342)	-3.224 (-7.554, 0.8646)	SUs	-3.806 (-7.673, 0.6829)	-0.9849 (-4.875, 3.485)	-1.791 (-5.976, 2.246)
1.335 (-6.31, 9.141)	3.453 (-1.71, 8.061)	0.5248 (-2.908, 3.355)	3.806 (-0.6829, 7.673)	TZD	2.795 (-0.2527, 5.829)	1.94 (-2.086, 5.427)
-1.456 (-8.91, 6.152)	0.6425 (-4.097, 4.777)	-2.274 (-5.712, 0.5881)	0.9849 (-3.485, 4.875)	-2.795 (-5.829, 0.2527)	control	-0.8377 (-4.096, 1.889)
-0.5946 (-8.002, 7.288)	1.46 (-2.93, 5.838)	-1.441 (-5.282, 2.363)	1.791 (-2.246, 5.976)	-1.94 (-5.427, 2.086)	0.8377 (-1.889, 4.096)	metformin

5.2 ALT						
DPP4	0.3774 (-14.01, 14.52)	-2.343 (-18.23, 13.2)	6.099 (-10.81, 22.72)	-7.49 (-23.83, 7.97)	0.8563 (-14.2, 16.04)	2.642 (-12.89, 18.04)
-0.3774 (-14.52, 14.01)	GLP1	-2.59 (-13.47, 7.701)	5.829 (-5.395, 16.84)	-7.816 (-18.89, 2.389)	0.5344 (-9.163, 10.18)	2.274 (-7.667, 12.04)
2.343 (-13.2, 18.23)	2.59 (-7.701, 13.47)	SGLT2i	8.392 (-1.064, 18.31)	-5.228 (-11.57, 1.011)	3.136 (-2.86, 9.704)	4.895 (-2.186, 12.69)
-6.099 (-22.72, 10.81)	-5.829 (-16.84, 5.395)	-8.392 (-18.31, 1.064)	SUs	-13.62 (-23.54, -4.163)	-5.317 (-14.89, 4.658)	-3.541 (-13.13, 6.486)
7.49 (-7.97, 23.83)	7.816 (-2.389, 18.89)	5.228 (-1.011, 11.57)	13.62 (4.163, 23.54)	TZD	8.347 (2.538, 14.87)	10.12 (2.851, 18.06)
-0.8563 (-16.04, 14.2)	-0.5344 (-10.18, 9.163)	-3.136 (-9.704, 2.86)	5.317 (-4.658, 14.89)	-8.347 (-14.87, -2.538)	control	1.768 (-4.408, 8.035)
-2.642 (-18.04, 12.89)	-2.274 (-12.04, 7.667)	-4.895 (-12.69, 2.186)	3.541 (-6.486, 13.13)	-10.12 (-18.06, -2.851)	-1.768 (-8.035, 4.408)	metformin
